# Prebiotically Plausible RNA Activation Compatible with Ribozyme‐Catalyzed Ligation

**DOI:** 10.1002/anie.202010918

**Published:** 2020-12-10

**Authors:** Emilie Yeonwha Song, Eddy Ivanhoe Jiménez, Huacan Lin, Kristian Le Vay, Ramanarayanan Krishnamurthy, Hannes Mutschler

**Affiliations:** ^1^ Max Planck Institute of Biochemistry Am Klopferspitz 18 82152 Martinsried Germany; ^2^ Department of Chemistry The Scripps Research Institute 10550 North Torrey Pines Road La Jolla CA 92037 USA; ^3^ Technical University Dortmund Otto-Hahn-Strasse 4a 44227 Dortmund Germany

**Keywords:** diamidophosphate, early Earth, prebiotic chemistry, ribozymes, RNA

## Abstract

RNA‐catalyzed RNA ligation is widely believed to be a key reaction for primordial biology. However, since typical chemical routes towards activating RNA substrates are incompatible with ribozyme catalysis, it remains unclear how prebiotic systems generated and sustained pools of activated building blocks needed to form increasingly larger and complex RNA. Herein, we demonstrate in situ activation of RNA substrates under reaction conditions amenable to catalysis by the hairpin ribozyme. We found that diamidophosphate (DAP) and imidazole drive the formation of 2′,3′‐cyclic phosphate RNA mono‐ and oligonucleotides from monophosphorylated precursors in frozen water‐ice. This long‐lived activation enables iterative enzymatic assembly of long RNAs. Our results provide a plausible scenario for the generation of higher‐energy substrates required to fuel ribozyme‐catalyzed RNA synthesis in the absence of a highly evolved metabolism.

## Graphical abstract

A plausible scenario for the prebiotic generation of activated RNA substrates under mild aqueous conditions is presented. Using water‐soluble diamidophosphate, in situ production of 2′,3′‐cyclic phosphate‐activated oligoribonucleotides and their subsequent ligation by a ribozyme can be achieved.

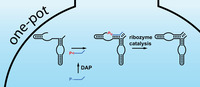

Modern cells have evolved elaborate metabolic networks in mild aqueous conditions to ensure their self‐preservation and to sustain their pool of activated building blocks. In contrast, it remains unclear how analogous (re‐)activation of building blocks in primitive precursor systems could have been possible without access to sophisticated protein enzymes. Given a likely central role of RNA catalysts (ribozymes) during the Origin of Life,^[1]^ robust prebiotic processes must have provided pools of activated mono‐ and oligonucleotides for activities such as RNA self‐replication.

In the laboratory, RNA synthesis by ribozymes is achieved through the ligation of pre‐activated RNA substrates. For example, some artificial ribozymes obtained by in vitro selection utilize 5′‐triphosphate activation chemistry for phosphodiester formation.[^2^, ^3^, ^4^] Their substrates are typically obtained from in vitro transcription reactions involving RNA polymerase proteins and retain the 5′‐triphosphate of the first nucleotide. In vitro selection experiments also yielded ribozymes that can triphosphorylate specific RNA substrates using trimetaphosphate.^[5]^ However, credible non‐enzymatic pathways for high‐yielding 5′‐triphosphate activation of RNA are currently missing.[^6^, ^7^] Other ligase ribozymes utilize short‐lived 5′‐phosphoramidate‐activated RNA. These substrates are obtained through dedicated pre‐activation of 5′‐phosphorylated RNA with reagents such as imidazole derivatives,[^8^, ^9^, ^10^] and either labile and nonspecific carbodiimides or prebiotically implausible 2,2‐dipyridyl disulfide and triphenyl phosphine.^[11]^ Thus, robust chemical pathways that yield activated RNA under conditions that also enable ribozyme activity remain elusive.

Diamidophosphate (DAP) was recently identified as a promising water‐stable and prebiotically plausible reagent to phosphorylate biological building blocks such as nucleic acids, amino acids, and lipid precursors.[^12^, ^13^] Of particular interest for the activation of RNA is the ability of DAP to produce nucleoside 2′,3′‐cyclic monophosphates (>P), which can subsequently polymerize into short RNA oligonucleotides without the need for additional activating reagents. Enthalpically, >P‐dependent formation of RNA phosphodiester bonds is favored due to the small amount of energy stored in the strained 2′,3′‐cyclic phosphate, but is disfavored entropically.[^14^, ^15^] However, stable substrate binding and/or low temperatures can compensate for the entropic costs and lead to a strong shift in reaction equilibrium towards ligation.[^16^, ^17^] Consequently, >P‐activated mono‐ and oligoribonucleotides may act as potent building blocks for primitive ribozyme‐catalyzed[^17^, ^18^, ^19^, ^20^] and even spontaneous RNA ligation reactions that increase the diversity and length distribution of RNA polymer chains.[^21^, ^22^] The mild conditions required for DAP‐dependent phosphorylation provide an attractive approach to activate free 3′‐termini of RNA for ligation reactions in the context of primitive metabolism. Under solution conditions and high millimolar concentrations of DAP, nucleoside, and metal chloride, quantitative >P formation is observed within days to weeks.^[12]^

However, high levels of metal ions such as Mg^2+^, which are required for efficient >P formation under aqueous conditions, are incompatible with half‐lives of ribozymes in water.^[23]^

Here, we demonstrate RNA activation by DAP under conditions that are amenable to the catalytic activity of >P‐dependent ribozymes. In particular, we found that the concentrating and preserving environment of water‐ice enables efficient formation of >P‐activated RNA. The activated RNA can be used in situ for RNA ligation by derivatives of the naturally occurring hairpin ribozyme (HPz), which serves as a versatile model system for prebiotic RNA ligation.[^17^, ^18^, ^19^]

To explore the potential of water‐ice in combination with DAP to enable >P RNA formation, we incubated individual canonical ribonucleoside 3′‐monophosphates (3′‐NMPs) with DAP and imidazole (or its derivatives 2‐amino‐ and 2‐methylimidazole) at −20 °C and monitored the reactions using ion‐exchange liquid chromatography (Figures S25–S72). In solution, DAP reacts with phosphates to form the corresponding amidopyrophosphate. This intermediate forms the 2′,3′‐cyclophosphate in the presence of a 2′‐OH group, with the amidophosphate as the leaving group (Scheme 1).^[12]^ Intriguingly, we observed moderate to good yields for the U, C, A, and G>Ps after 28 days (Figures 1 a, S1–S12) confirmed by ^31^P NMR (Figures S73,74). Among the three activators tested in this study, imidazole‐containing reactions were faster (Figures S1–S12) and yielded the highest amounts of >Ps (Figures 1 a, S13–S16) under the four pH conditions tested. We also note remarkable differences in the efficiency of >P formation depending on the nucleobase (Figure 1 b). After 7 days at pH 6 in the presence of imidazole, approximately 50 % of both pyrimidine nucleotides 3′‐UMP and 3′‐CMP were converted into their respective >Ps. In contrast, conversion of the purine nucleotides under the same conditions was much slower with circa 33 % for 3′‐AMP and only <3 % for 3′‐GMP. After 28 days, the conversion yield for 3′‐UMP, 3′‐CMP, and 3′‐AMP reached 72–88 % while it approached 30 % for 3′‐GMP. See below for further mechanistic explanations for the observed influence of the nucleobase moieties.

**Figure 1:**
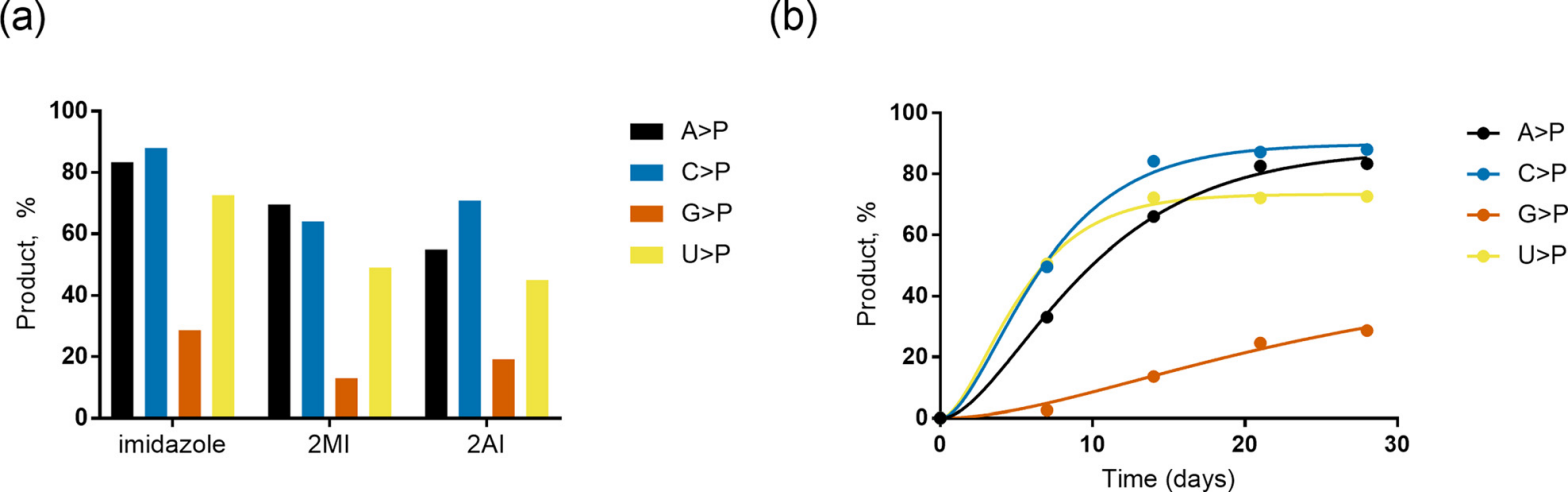
a) Comparison of activator performance in the formation of N>Ps from their respective 3′‐NMPs (1 mm) after 28 days at pH 6 and −20 °C in the presence of 1 mm DAP, 5 mm MgCl_2_, and 5 mm of either imidazole, 2‐methylimidazole (2MI), or 2‐aminoimidazole (2AI); similar trends were observed at pH 5, 7, and 8 (Figures S13–S16). b) Percentage conversion to >P as a function of time with imidazole as activator at pH 6.

**Scheme 1:**
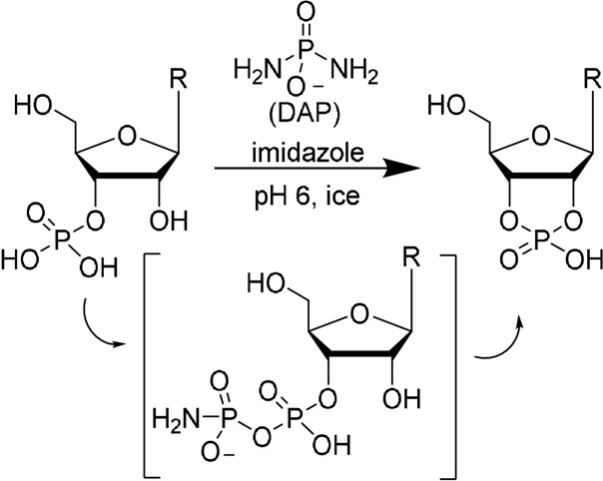
Diamidophosphate‐mediated formation of 2′,3′‐cyclic phosphate ribonucleotides under conditions compatible with ribozyme catalysis.

Having shown that DAP can be used as an efficient activation agent for N>P formation in frozen water‐ice systems, we sought to explore whether DAP can also activate oligonucleotides to fuel ribozyme‐catalyzed ligation reactions. Previous studies have shown that HPzs are capable of efficient >P‐dependent in‐ice ligation of RNA substrates.^[18]^ Thus, we probed whether in‐ice activation of RNA substrates by DAP could enable direct downstream HPz‐dependent ligation reactions.

To monitor the coupled activation–ligation reactions, we developed a reporter electrophoretic mobility shift assay (EMSA) based on a *cis*‐ligating version of the HPz (**cHPz**, Figures 2, S17). We confirmed the self‐ligation activity of **cHPz** using >P RNA (**sub>P**) generated with ethyl‐3‐(3′‐dimethylaminopropyl)‐carbodiimide (EDC), with which circa 88 % self‐ligation was observed via urea‐PAGE in reaction conditions used in previous HPz in‐ice studies (Figure S18).^[18]^ No band shift was observed in the presence of **sub‐P**, the non‐activated 3′‐monophosphate RNA.

**Figure 2:**
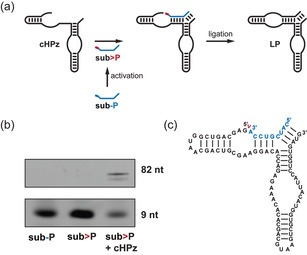
A reporter electrophoretic band shift assay based on a modified hairpin ribozyme (**cHPz**) which accepts a 2′,3′‐cyclic phosphate substrate (**sub>P**) for its catalytic activity. a) A simplified schematic of the reaction during which the substrate (**sub‐P**) is activated to yield the 2′,3′‐cyclic phosphate RNA oligomer. LP=ligation product. b) Denaturing EMSA of the species shown in (a). **sub‐P** is 5′‐tagged with a fluorescein derivative to aid in analysis. c) A detailed secondary structure of the substrate–ribozyme complex prior to ligation. The location of the 2′,3′‐cyclic phosphate and site of ligation are indicated in red.

Following the implementation of the reporter EMSA, we sought to test the efficacy of the coupled DAP activation–ligation reactions under various conditions. Specifically, we tested a range of concentrations of DAP, Mg^2+^, and imidazole for their potential to enable coupled activation–ligation reactions. We identified the inclusion of 5 mm DAP, 5 mm Mg^2+^, and 5 mm imidazole as optimal conditions thus far for the combined activation and ligation of the RNA substrate **sub‐P** (Figure S19), and used these conditions in all further experiments. We also noted an increased ligation yield at 50 mm DAP in the absence of imidazole. We presumed that the increased concentration of DAP, especially in eutectic ice, compensates for the absence of imidazole. However, increasing both DAP and imidazole concentrations to 50 mm resulted in a lower ligation yield compared to 5 mm (Figure S20). This result may be due to the high DAP and imidazole concentrations that reduce the concentration‐by‐freezing efficiency for the RNA components. The higher initial concentration of the two components reduces the amount of water‐ice necessary to reach the molal equilibrium concentrations of the unfrozen phase.^[24]^ Thus, the resulting cHPz and substrate concentrations in the aqueous phase may be lower than with decreased DAP and imidazole concentrations, leading to less efficient activation and/or ligation.

We then explored the pH dependency of the activation–ligation reaction. We speculated that a highly acidic environment may decrease the availability of the DAP‐activating agent imidazole (p*K*_a_=6.95^[25]^), whose nucleophilicity decreases upon protonation. Moreover, the activity of HPz decreases below pH 5^[26]^ while the stability of RNA is optimal at pH 4–5. Indeed, we did not detect significant RNA degradation during the experiment (Figure S21). Consistent with these considerations and with our results with monomeric ribonucleotides, we observed the highest ligation yields and reaction rates at pH 5–6 (Figures 3 and S22). We postulate that a nucleophilic pool of unprotonated imidazole is maintained at this mildly acidic pH while preserving the activity of cHPz. The decrease in yields at pH above 6 likely reflects that >P formation requires protonation of the ‐NH_2_ group of DAP (p*K*_a_≈5.5).^[27]^ This assumption is supported by the pH dependence of the activation of 3′‐NMPs (Figures S1–S12). While we note that it is typically difficult to estimate the pH of the interstitial brine in water‐ice accurately, we expect that the pH in our buffered samples is only increased by about 0.5 pH units at −9 °C.^[28]^

**Figure 3:**
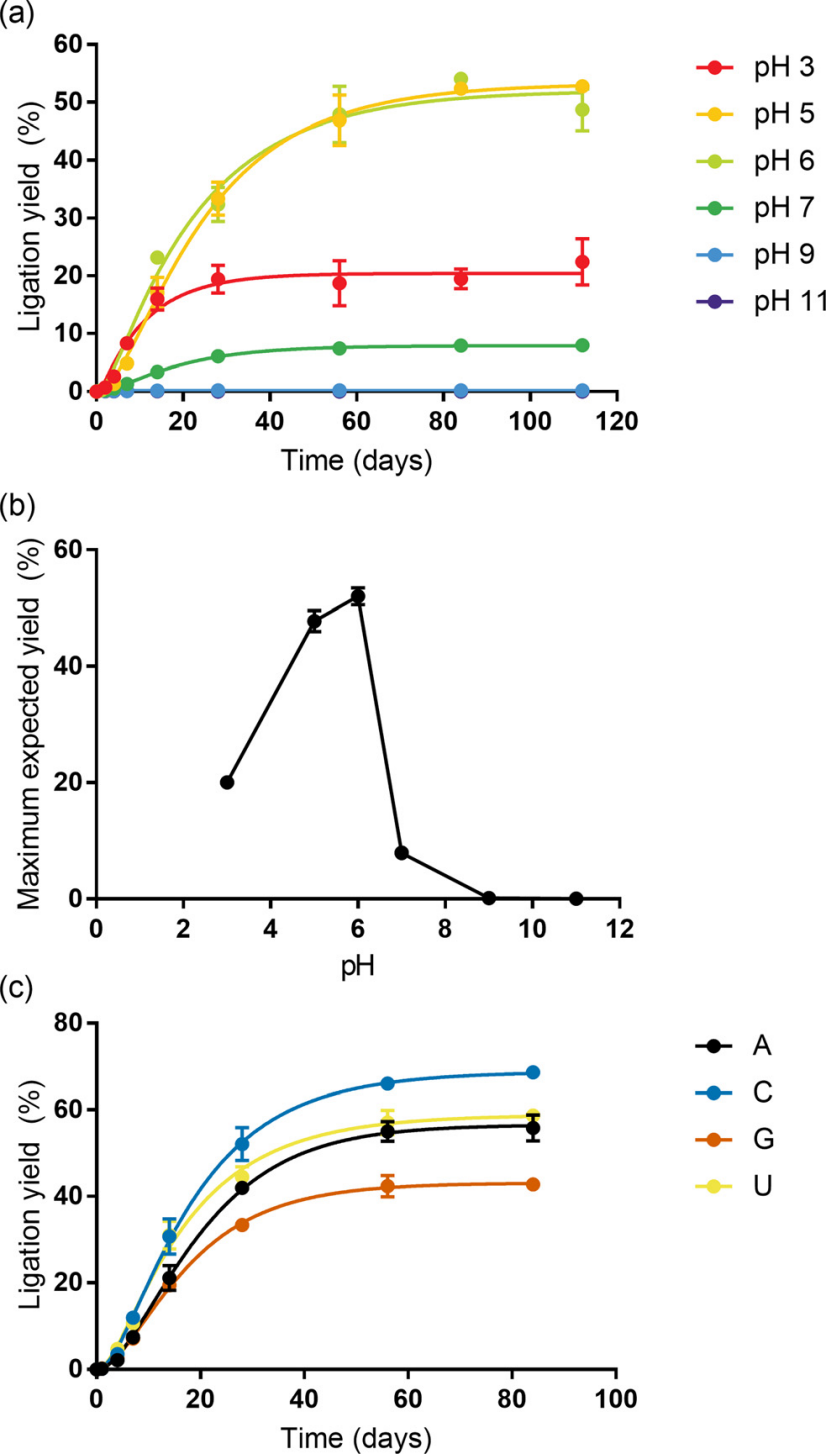
DAP‐mediated formation of 2′,3′‐cyclic phosphate RNA and its subsequent ligation in the presence of imidazole occur under various conditions compatible with ribozyme catalysis and are optimal at pH 5–6. a) Time course analysis of in situ activation–ligation reactions at different pH values. Reaction mixtures containing 10 mm of glycine⋅HCl buffer (pH 3), MES buffer (pH 5–7), Tris⋅HCl buffer (pH 9), or carbonate/bicarbonate buffers (pH 11) were analyzed via TBE‐urea PAGE. Data points were fitted assuming a simplified 2‐step reaction mechanism to extrapolate maximum ligation yields. b) pH dependency of the maximum ligation yield using the best‐fit parameters from (a). Error bars indicate the 95 % confidence intervals. c) Dependency of coupled activation–ligation reactions on the 3′‐terminal base of **sub‐P**. Reactions were performed using four 2′/3′‐monophosphorylated variants and analyzed via TBE‐urea PAGE.

We initially observed a considerable influence of the base moiety on DAP‐mediated phosphate activation for the four mononucleotides (Figure 1 b). Therefore, we wondered whether a similar underlying nucleobase dependency might govern the DAP activation of RNA oligonucleotides in the EMSA analysis. Indeed, we observed increased ligation yields for 3′‐terminal pyrimidines over purines (Figure 3 c). The difference was most obvious between substrates with a 3′‐terminal C and G: After 84 days, the 3′‐terminal pyrimidine resulted in 68 % ligation yield compared to the purine at 43 %. The yield differences are unlikely to be the result of a preference of the cHPz for different 3′ ends, because the influence of the 3′ base identity was only marginal in control experiments with pre‐activated substrates (Figure S23). While the solubilities of monophosphates (CMP, 16.3 g L^−1^; UMP, 12 g L^−1^; AMP, 8 g L^−1^; GMP, 8 g L^−1^)^[29]^ may be a factor for the different activation yields at the monomer level, this is unlikely with oligonucleotides. p*K*_a_ values of the 2′‐OH group, between 13.22 and 13.47,^[30]^ would predict minimal differences in the nucleophilicity of the 2′‐OH groups of the four nucleotides for the formation of >Ps. However, it is possible that a larger fraction of the nucleobases A and C is protonated at pH 5–6 and becomes more electron‐withdrawing, thereby increasing the acidity of the 2′‐OH group.^[31]^ For U, the 2‐keto‐oxygen may coordinate with the 2′‐OH group, thus facilitating its deprotonation.^[32]^

To further test the generality of the reaction, we investigated the influence of the co‐activator imidazole on substrate activation. 2‐methylimidazole (2MI) and 2‐aminoimidazole (2AI) are close analogues of imidazole (IMI) that are considered as superior 5′‐leaving groups for non‐enzymatic copying of RNA templates.[^33^, ^34^] We repeated our EMSA assay with 5 mm 2MI or 2AI instead of IMI (Figure S25) and found that ligation yields were considerably lower (approximately 25 % after 84 days) in the presence of 2AI or 2MI compared to IMI (approximately 55 %). The lower p*K*_a_ of IMI (6.95 compared to 8.46 for 2AI^[35]^ and 7.86 for 2MI^[36]^)—and therefore its larger unprotonated nucleophilic pool—coupled with less steric hindrance may amplify the formation of the activated imidazole‐amidophosphate that is responsible for phosphorylation. Nevertheless, our results show that different imidazole derivatives can serve as activators for the DAP‐dependent generation of cyclic phosphates.

Next, we examined the performance of DAP compared to the prebiotically implausible carbodiimide EDC, which is typically used to generate >Ps. Intriguingly, the long‐term kinetics of RNA activation under in situ ribozyme catalysis‐compatible conditions showed higher ligation yields with DAP compared to EDC (Figure 4 a). While one‐pot in situ activation with EDC caused higher initial yields during the first 10 days of incubation, likely due to higher reactivity of the carbodiimide moiety, the yields in the DAP‐based reactions significantly exceeded those incubated with EDC during long‐term incubation, reaching >50 % more RNA ligation after 28 days. A plausible explanation for this observation is the overall low stability of EDC due to hydrolysis, which hampers reactivation of hydrolyzed cyclic phosphates. In contrast, the high stability of DAP may allow repeated reactivation of hydrolyzed 3′‐terminal phosphates back to >Ps. Furthermore, EDC modifies nucleic acid base moieties at rates comparable to those of RNA‐catalyzed ligation.[^37^, ^38^, ^39^] In contrast, we observed no indication of similar undesirable side reactions with DAP.

**Figure 4:**
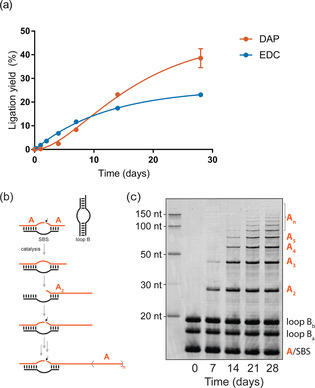
a) Comparison of DAP and EDC under in situ activation–ligation conditions. Reaction mixtures differing only in the inclusion of either EDC or DAP were analyzed via TBE‐urea PAGE. The data points were fitted assuming a simplified irreversible 2‐step reaction mechanism. b) Simplified schematic of 16 nt RNA oligomer concatenation. SBS=substrate‐binding strand. c) DAP‐mediated RNA concatenation. Time course analysis via SYBR Gold‐stained TBE‐urea PAGE of reaction mixtures containing a fragmented ribozyme and 16 nt 3′‐monophosphorylated substrates. We note that the RNA ladder displays lower electrophoretic mobility, presumably due to different salt concentrations in the loading buffer and/or conjugated fluorophores.

Finally, we investigated whether DAP activation also allows multiple ligations to produce long RNAs, which is a prerequisite for the formation of more complex RNA molecules. To this end, we probed our one‐pot activation/ligation scheme for concatenation reactions of RNA oligomers (Figures 4b,c and S26), including the assembly of an RNA polymerase ribozyme from seven ≤30 nt fragments (RPR7, Figure S26) catalyzed by fragmented hairpin ribozymes.^[19]^ Strikingly, for both reactions we observed six or more successive ligation events after 21 days of in‐ice incubation.

The data herein present DAP‐mediated formation of activated RNA substrates under conditions compatible with ribozyme catalysis. DAP is an attractive candidate reagent for the prebiotic activation of RNA due to its long half‐life and reactivity in aqueous environments.^[40]^ Moreover, its potential in the primordial activation of lipids, peptides, and 5′ ends of nucleic acids—and therefore as a key reagent in the origin of all life on Earth—has been documented.[^12^, ^13^] Furthermore, the compatibility of the reaction with frozen water‐ice matrices that upconcentrate solutes allows low initial DAP and imidazole concentrations to achieve high‐yielding activation of mono‐ and oligonucleotides. DAP is therefore a powerful reagent to mitigate quasi‐irreversible hydrolysis of 2′,3′‐cyclic phosphates that occurs over time in the presence of M^2+^ ions,^[41]^ and to sustain a pool of activated >P RNA molecules that can serve as starting material for both enzymatic and non‐enzymatic ligation reactions.

>P RNA can also be formed by enzymatic or non‐enzymatic RNA cleavage. In combination with strand dissociation, consecutive cleavage–ligation reactions may therefore lead to the formation of longer products.[^19^, ^22^, ^42^, ^43^, ^44^] While offering an intriguing alternative for RNA synthesis in the absence of direct chemical activation, such recombination reactions have several disadvantages. The extra cleavage and dissociation steps slow product formation and decrease final yields due to a higher number of reversible steps required for product formation.[^19^, ^22^] Moreover, ribozyme‐catalyzed recombination requires the presence of additional sequence elements on substrates which reduces the pool of compatible oligonucleotides in randomized libraries. Finally, recombination does not regenerate hydrolyzed >Ps. Thus, while both direct ligation and recombination are capable of exploring a large sequence space through repeated ligation reactions,^[22]^ both reactions can ultimately benefit from the mild re‐activating properties of DAP.

The resulting high‐diversity products could provide the starting material for evolutionary processes in the form of nascent ribozymes.^[22]^ Additionally, continuous activation of RNA substrates provides the possibility of maintaining cross‐catalytic reaction networks consisting of several ribozyme components^[19]^ and may enable new strategies for continuous RNA evolution under prebiotically plausible conditions. In conclusion, our work underlines the importance of identifying ribozyme catalysis‐compatible RNA activation reagents in enabling one‐pot processes, which has implications for abiotic molecular evolution of nucleic acids.

## Conflict of interest

The authors declare no conflict of interest.

## Supporting information

As a service to our authors and readers, this journal provides supporting information supplied by the authors. Such materials are peer reviewed and may be re‐organized for online delivery, but are not copy‐edited or typeset. Technical support issues arising from supporting information (other than missing files) should be addressed to the authors.

Supplementary
