# Supplementary material for: Prebiotically Plausible RNA Activation Compatible with Ribozyme‐Catalyzed Ligation
Source: Angew Chem Int Ed Engl. 2020 Dec 10;60(6):2952–7. doi: 10.1002/anie.202010918 (PMC7898671; doi:10.1002/anie.202010918)
Supplement: Supplementary file 1 — Supplementary [file ANIE-60-2952-s001.pdf]

Angewandte Chemie
Eine Zeitschrift der Gesellschaft Deutscher Chemiker

Supporting Information

Prebiotically Plausible RNA Activation Compatible with Ribozyme-Catalyzed Ligation

Emilie Yeonwha Song, Eddy Ivanhoe Jiménez+, Huacan Lin+, Kristian Le Vay, Ramanarayanan Krishnamurthy, and Hannes Mutschler**

anie_202010918_sm_miscellaneous_information.pdf

Table of contents

1. Experimental section 3
1.1 Synthesis of 2’,3’-cNMP from respective 3’-NMP 3
1.2 *In situ* DAP activation and cHPz ligation experiments 4
1.3 TBE-Urea PAGE EMSA 5
1.4 Curve fitting 6
1.5 Ion-exchange column chromatography 6
2. Conversion to 2’,3’-cNMP (%) vs. time 7
2.1 3’-UMP 7
2.2 3’-CMP 10
2.3 3’-AMP 13
2.4 3’-GMP 16
3. Comparison of activators at week 4 19
3.1 3’-UMP 19
3.2 3’-CMP 20
3.3 3’-AMP 21
3.4 3’-GMP 22
4. General scheme of electrophoretic band shift assay 23
5. Ligation kinetics of cHPz 24
6. DAP-mediated ligation 25
7. IE-LC chromatograms for comparisons of activators 33
7.1 Chromatograms 3’-UMP → 2’,3’-cUMP with different activators (weeks 1 to 4) 33
7.2 Chromatograms 3’-CMP → 2’,3’-cCMP with different activators (weeks 1 to 4) 46
7.3 Chromatograms 3’-AMP → 2’,3’-cAMP with different activators (weeks 1 to 4) 59
7.4 Chromatograms 3’-GMP → 2’,3’-cGMP with different activators (weeks 1 to 4) 72
8. ^{31}P NMR 85
9. Supplementary references 87
10. Author Contributions 88

1. Experimental section

All starting materials were used directly as obtained commercially from Sigma-Aldrich and Fisher Scientific. NMR recorded at 298 K on a Bruker DPX-400; chemical shifts (δ) reported in parts per million (ppm) and coupling constants given in Hertz. Anion Exchange Chromatography (AEC) was performed with AKTA purifier (900) with UNICORN system control using 260 nm on a DNApac^{TM}PA100 column with a flow rate of 1 mL/min using Buffer A: 5 mM Tris base, pH 8.2 and Buffer B: 5 mM Tris base, 330mM $NaClO_4$, pH 8.2. The gradient is 0 to 10% Buffer B in 10 min for the reactions with 3'-UMP, 3'-CMP and 3'-GMP whereas 0 to 2% Buffer B in 10 min for 3'-AMP. The samples were filtered through 0.22 µm syringe tip filter units (Milliex-GV; PVDF). Quantification of UV active species was performed with NanoDrop 2000c spectrometer. The pH was recorded using a pH electrode connected to pH meter (Hanna instrument from Spectrum Chemicals and Laboratory products).

1.1 Synthesis of 2',3'-cNMP from respective 3'-NMP

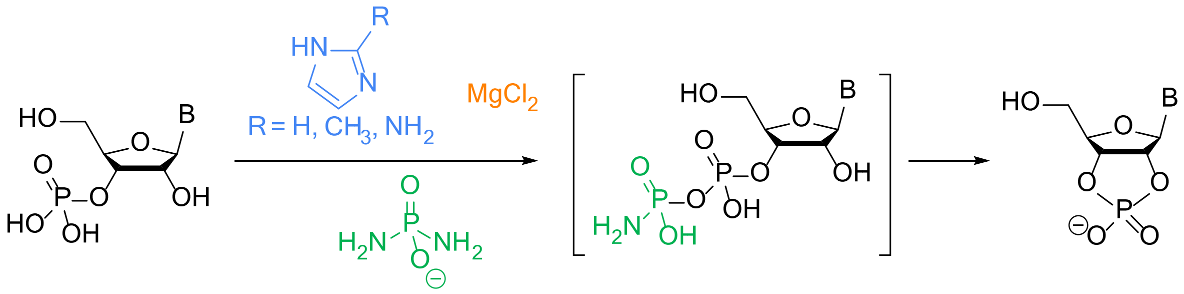

Fresh stock solutions of each substrate were made in water. Details of preparation of stock solutions are described as below.

1. 3'-NMP stock solution (10 mM). In the case of 3'-CMP the concentration was 5 mM.
2. $MgCl_2$ stock solution (50 mM)
3. DAP stock solution (50 mM)
4. Activator stock solution (imidazole, 2-methylimidazole, 2-aminoimidazole sulfate, 50 mM for each activator)

For each reaction, to an Eppendorf vial were added 100 µL of 3'-NMP stock solution (200 µL in the case of 3'-CMP), 100 µL $MgCl_2$ stock solution, 100 µL DAP stock solution and 100 µL activator stock solution, then 600 µL H_2O (500 µL in the case of 3'-CMP) was added for each reaction to make the final volume to 1 mL. pH was adjusted to 5, 6, 7, 8 with either 0.4 M HCl or 0.5 M NaOH. After mixing each reaction well, 30 µL reaction solution was aliquoted in four separate Eppendorf vials indicating week 1 to week 4 for monitoring. All Eppendorf vials were closed tightly and kept in a freezer (-20°C). The progress of the reactions was monitored by ion-exchange column chromatography.

1.2 *In situ* DAP activation and ribozyme ligation experiments

All RNA molecules were purchased from Integrated DNA Technologies. Unless otherwise noted, the reaction mixture (10 µM **cHPz**, 1 µM **sub-P**, 25 mM NaCl, 5 mM imidazole, 5 mM DAP, 5 mM $MgCl_2$, 10 mM pH 6 MES buffer) was prepared in biological triplicates at room temperature and aliquoted into separate PCR tubes containing 5 µL for each timepoint to be sampled. The tubes were frozen at -80 °C for 30 min then moved to a Grant Low temperature bath/circulator TXF-200 R5 set to incubate at -9 °C in 1:1 water/ethylene glycol. Reactions were stopped by the addition of 20 µL of RNA loading dye (0.5 mM EDTA in formamide, bromophenol blue) directly to frozen samples and allowing them to thaw while gently mixing. The samples were either stored at -80 °C or directly analyzed by urea-PAGE.

1.2.1 Additional ribozyme experiments – RNA concatenation

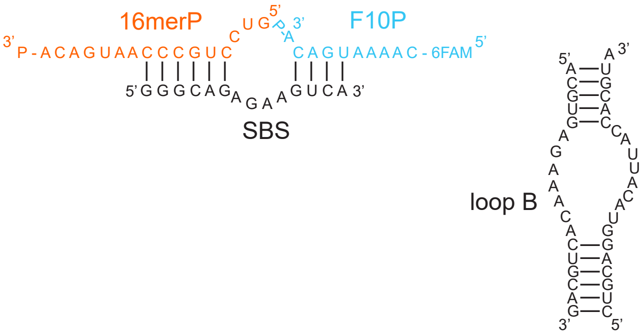

The reaction mixture (25 mM NaCl, 5 mM imidazole, 5 mM DAP, 5 mM $MgCl_2$, 10 mM pH 6 MES buffer) containing RNA oligomers[1] listed in Table 1 was prepared at room temperature and aliquoted into separate PCR tubes containing 10 µL for each timepoint to be sampled. Subsequent steps are identical to those described in **1.2**, with the exception of the volume of added RNA loading dye (40 µL).

RNAse R was used to test for circular concatemers in the reaction product. First, a sample of the final reaction mixture was purified using a Monarch RNA Cleanup Kit (NEB) and eluted in water. The sample was digested with RNAse R (20 U per 10 uL sample), 0.02 M Tris-HCl (pH 8.0), 0.1 M KCl, and 0.1 mM MgCl2 for 80 minutes at 37°C. The reaction was quenched by heating to 65°C for two minutes, then the addition of loading buffer (20 µL per 5 µL sample). Samples were directly analyzed by urea-PAGE.

Table 1. Sequences, concentrations, and descriptions of RNA oligomers used in RNA concatenation reactions.

RNA oligomer	Final concentration (µM)	Sequence (5' to 3')	Description
**F10P**	0.6	6FAM-CAAAAUGACA-P	6-carboxyfluorescein labelled reporter RNA used in concatenations.
**16merP**	6	GUCCUGCCCAAUGACA-P	16-mer repeat substrate used in concatenations.
**SBS16mer**	6	GGGCAGAGAAGUCA	Substrate-binding strand (SBS) used for concatenation of 16mer repeats
**loopB3**	6	CUGCAGGUACAUUACCACGUA	3' fragment of hairpin ribozyme loop B
**loopB5**	6	ACGUGAGAAACACUGCAG	5' fragment of hairpin ribozyme loop B

1.2.2 - Additional ribozyme experiments – RPR7 assembly

RNA polymerase ribozyme 7 (RPR7) assembly reactions were performed as previously described[1], with the use of 2'/3'-monophosphorylated RPR7 fragment oligomers in lieu of pre-EDC-activated >P RPR7 fragment oligomers. The frozen samples (25 mM NaCl, 5 mM imidazole, 5 mM DAP, 5 mM $MgCl_2$, 10 mM pH 6 MES buffer) were incubated at -9 °C for 14 days then subjected to freeze-thaw cycling conditions[1] thereafter.

1.3 TBE-Urea PAGE EMSA

Stock solutions of 10x TBE (108 g Trizma base, 55 g boric acid, 40 mL 0.5 M pH 8.0 EDTA, 1 L final volume, autoclaved) and 20 % TBE-urea (240 g urea, 250 mL 40 % acrylamide 19:1, 50 mL 10x TBE buffer, 500 mL final volume, sterile filtered) were prepared. 1 mm gels were cast 16 hours prior to use with 25 mL 20 % TBE-urea, 25 µL TEMED, and 250 µL 10 % ammonium persulfate in Atto gel electrophoresis kits. Samples were denatured at 95 °C for 2 minutes and immediately put on ice to prevent refolding. 1 µL of each sample was loaded onto the pre-cast gels then run at 5 W per gel for 10 minutes then 25 W per gel for 30 minutes in 1x TBE running buffer. The out-of-plate gels were either directly imaged on a Typhoon FLA 9000 Gel Imaging Scanner (GE Healthcare) or first stained for 10 min in 0.5X SYBR™ Gold in 1x TBE then imaged. Ligation yields were quantified using ImageQuant TL (GE Healthcare), corrected for fluorescence quenching in the ligation product (Figure S24), and analyzed using GraphPad Prism 7.

1.4 Curve fitting

A simplified quasi-irreversible 2-step reaction model based on the activation-ligation reaction (Figure 2a) was used during curve fitting of activation/ligation time traces:

$$\mathbf{sub-P} \overset{k_1}{\rightarrow} \mathbf{sub>P} \overset{k_2}{\rightarrow} \mathbf{LP}$$

The relative ligation yield of **LP** formed during the reaction was fit using equation 1:

$$Y = Y_{max}[1 + \left(\frac{1}{k_1 - k_2}\right)(k_2 e^{-k_1 t} - k_1 e^{-k_1 t})]$$

where Y_{max} is the maximum ligation yield and t the elapsed time in days.

In Figure 2, the curve for pH 3 was fitted using a single exponential (equation 2):

$$Y = Y_{max}(1 - e^{-kt})$$

1.5 Ion-exchange column chromatography

Anion Exchange Chromatography (AEC) was performed with AKTA purifier (900) with UNICORN system control using 260 nm on a DNApac^{TM}PA100 column with a flow rate of 1 mL/min using Buffer A: 5 mM Tris base, pH 8.2 and Buffer B: 5 mM Tris base, 330 mM $NaClO_4$, pH 8.2. The gradient is 0 to 10% Buffer B in 10 min for the reactions with 3'-UMP, 3'-CMP and 3'-GMP whereas 0 to 2% Buffer B in 10 min for 3'-AMP. The samples were filtered through 0.22 µm syringe tip filter units (Milliex-GV; PVDF).

2. Conversion to 2’,3’-cNMP (%) vs. time

as monitored by ion-exchange LC (see figures S24-S71)

2.1 3’-UMP

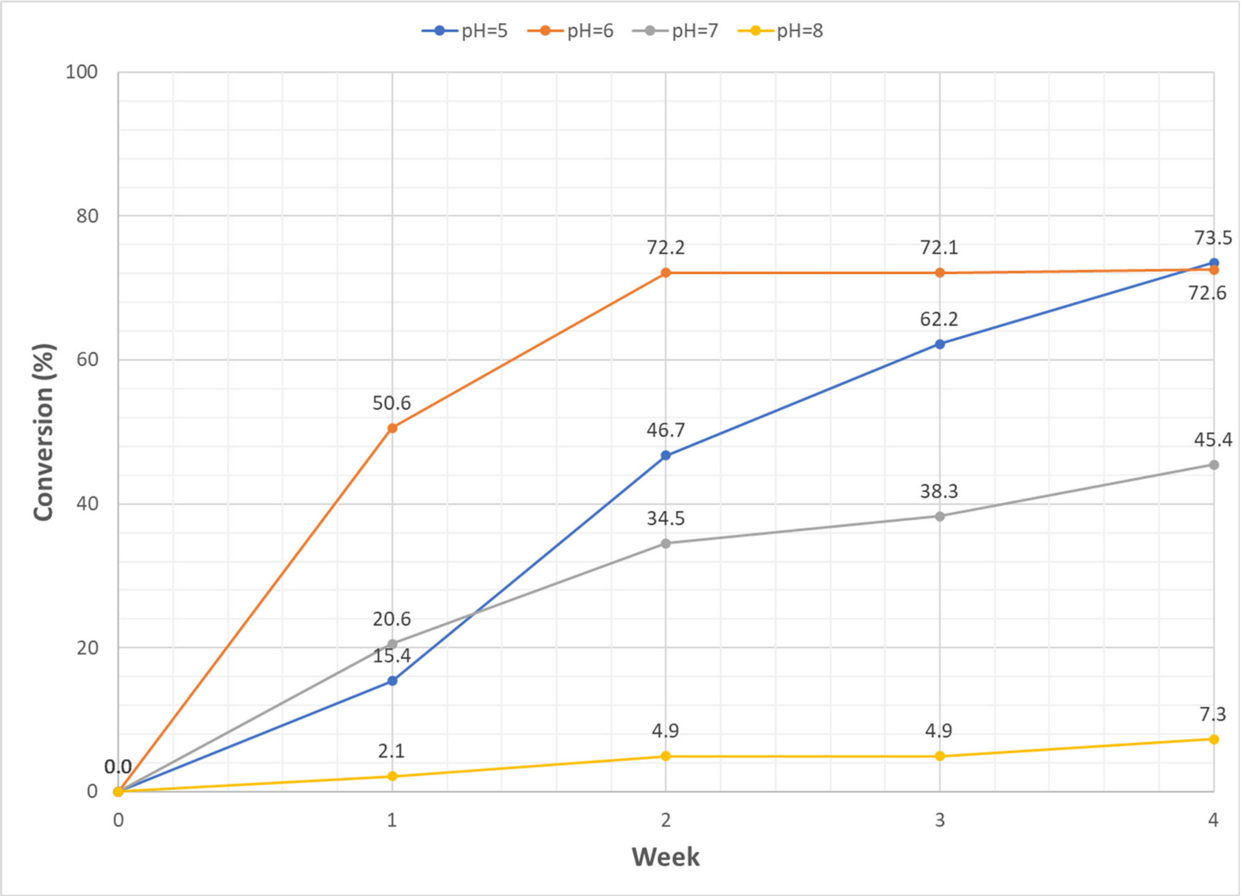

Figure S1. Percentage conversion to 2’,3’-cUMP as a function of time (weeks) at different pH with *imidazole* as activator.

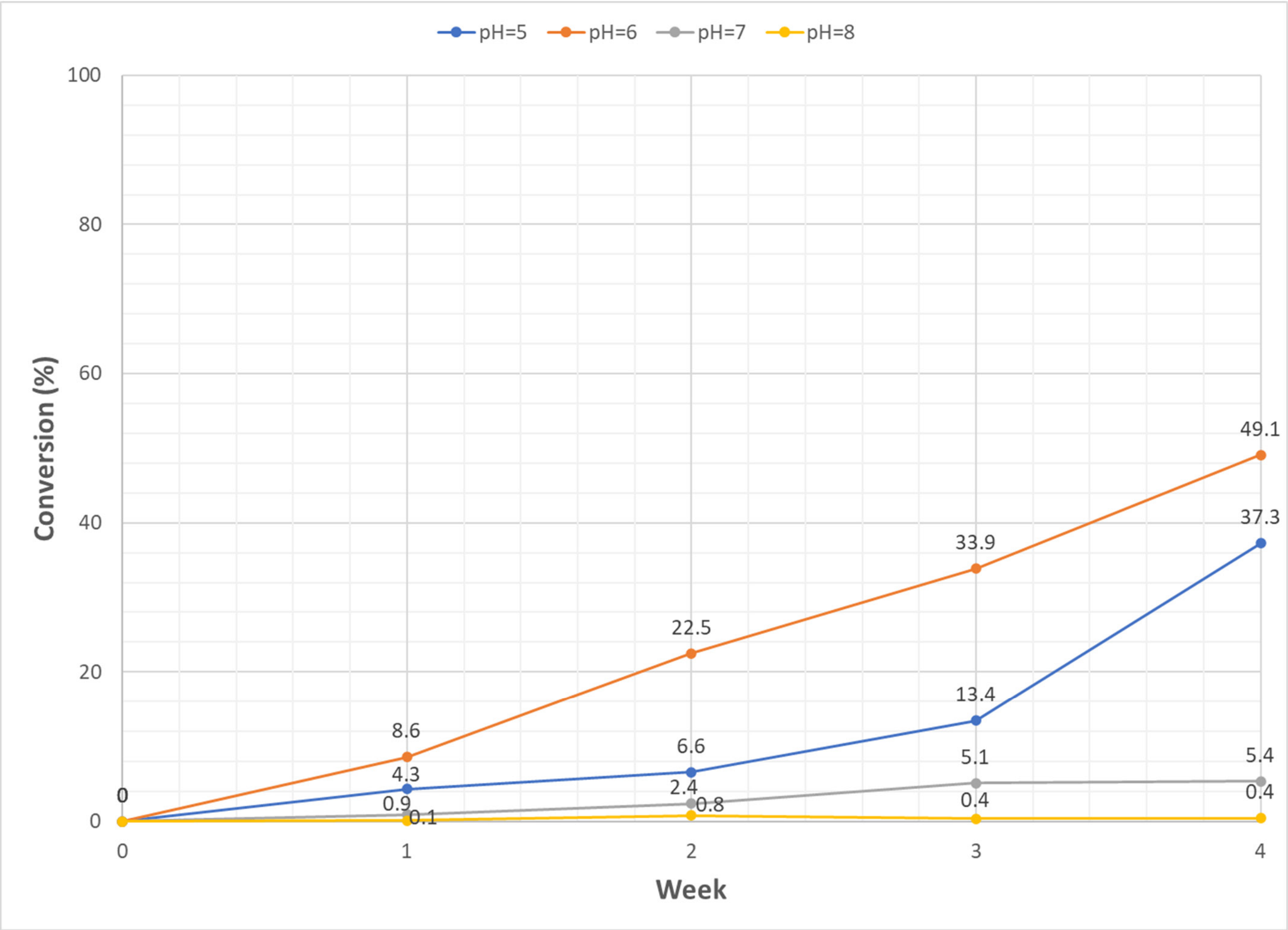

Figure S2. Percentage conversion to 2’,3’-cUMP as a function of time (weeks) at different pH with *2-methylimidazole* as activator.

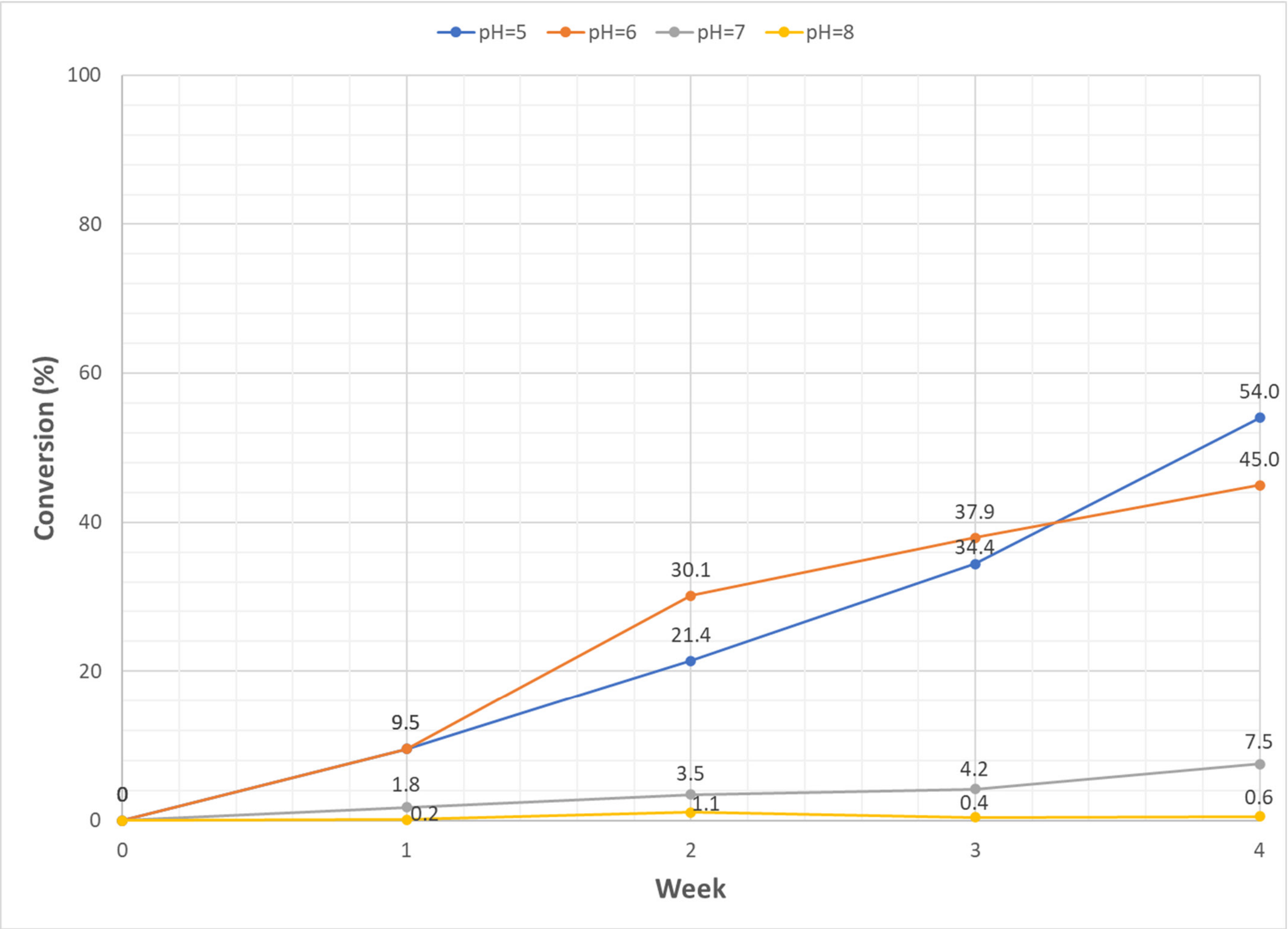

Figure S3. Percentage conversion to 2’,3’-cUMP as a function of time (weeks) at different pH with *2-aminoimidazole* as activator.

2.2 3’-CMP

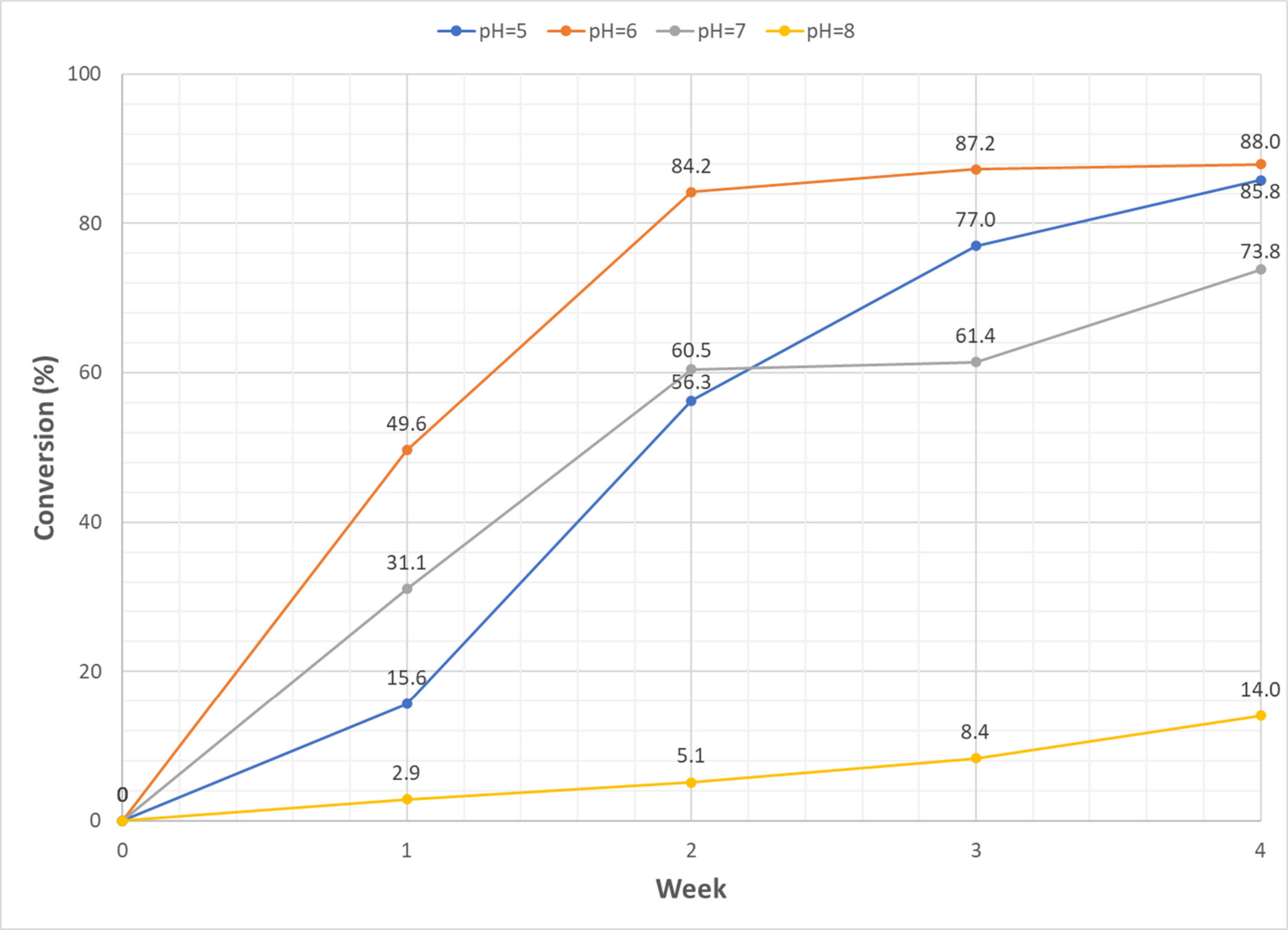

Figure S4. Percentage conversion to 2’,3’-cCMP as a function of time (weeks) at different pH with *imidazole* as activator.

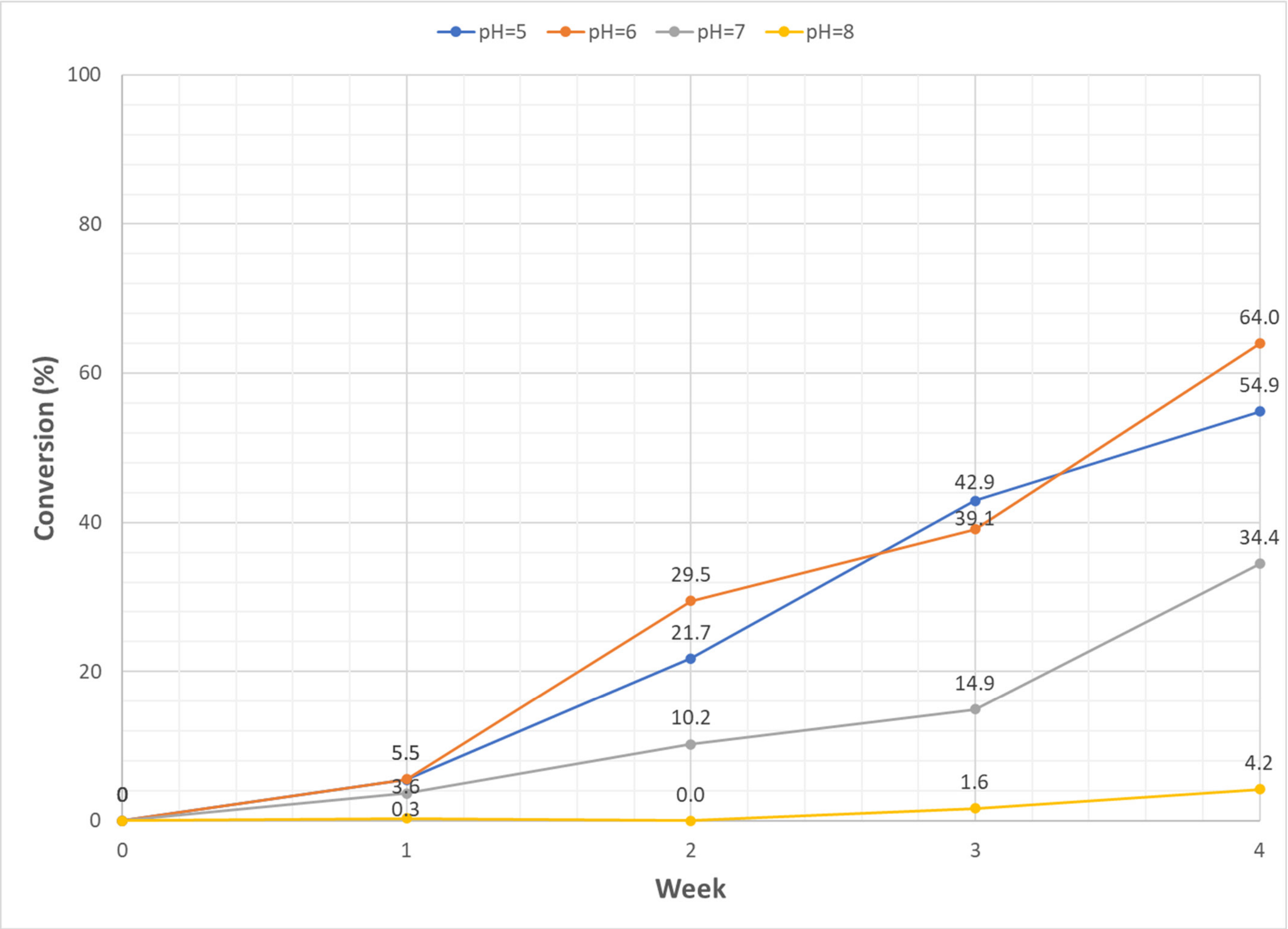

Figure S5. Percentage conversion to 2’,3’-cCMP as a function of time (weeks) at different pH with *2-methylimidazole* as activator.

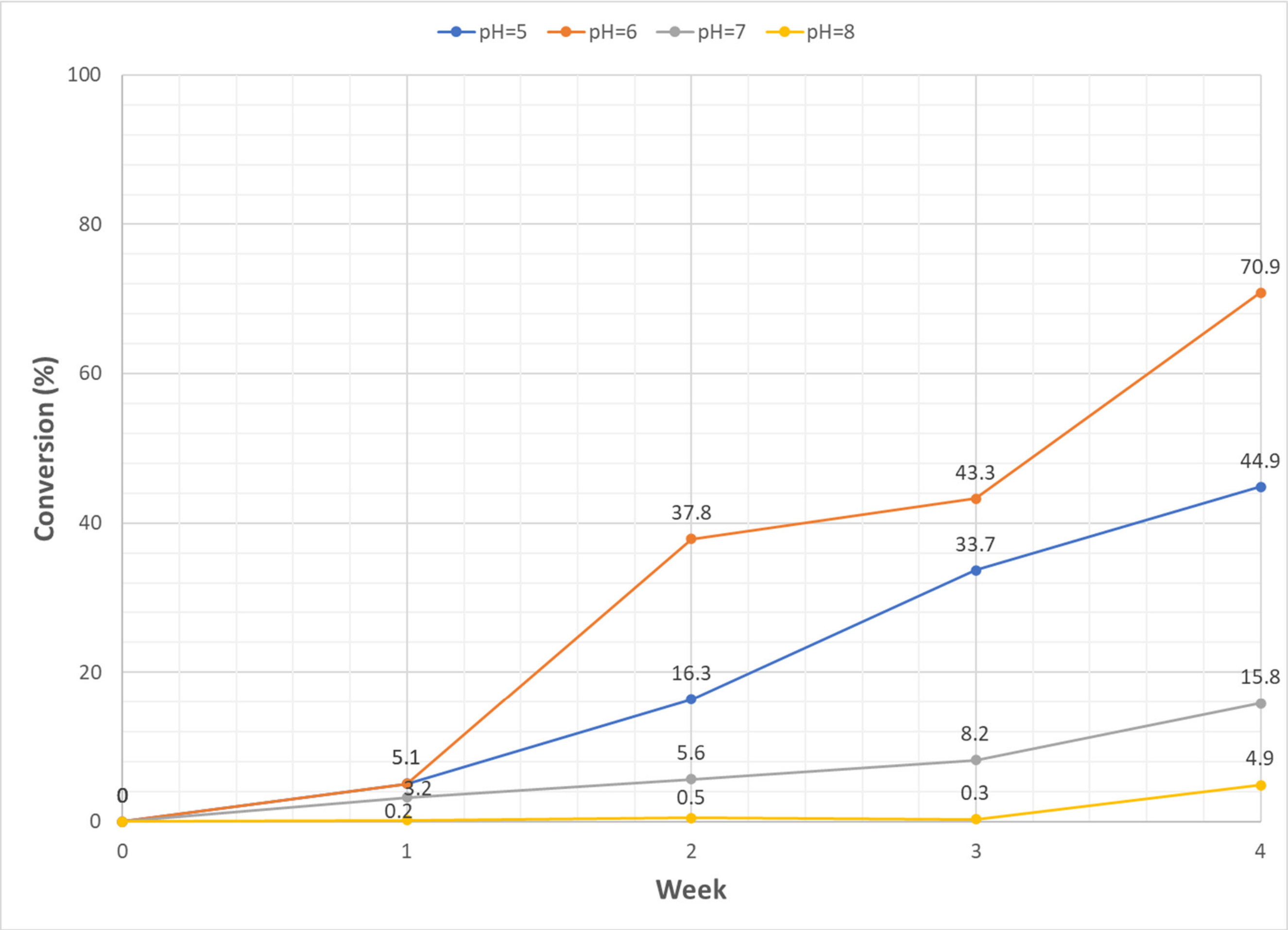

Figure S6. Percentage conversion to 2',3'-cCMP as a function of time (weeks) at different pH with *2-aminoimidazole* as activator.

2.3 3’-AMP

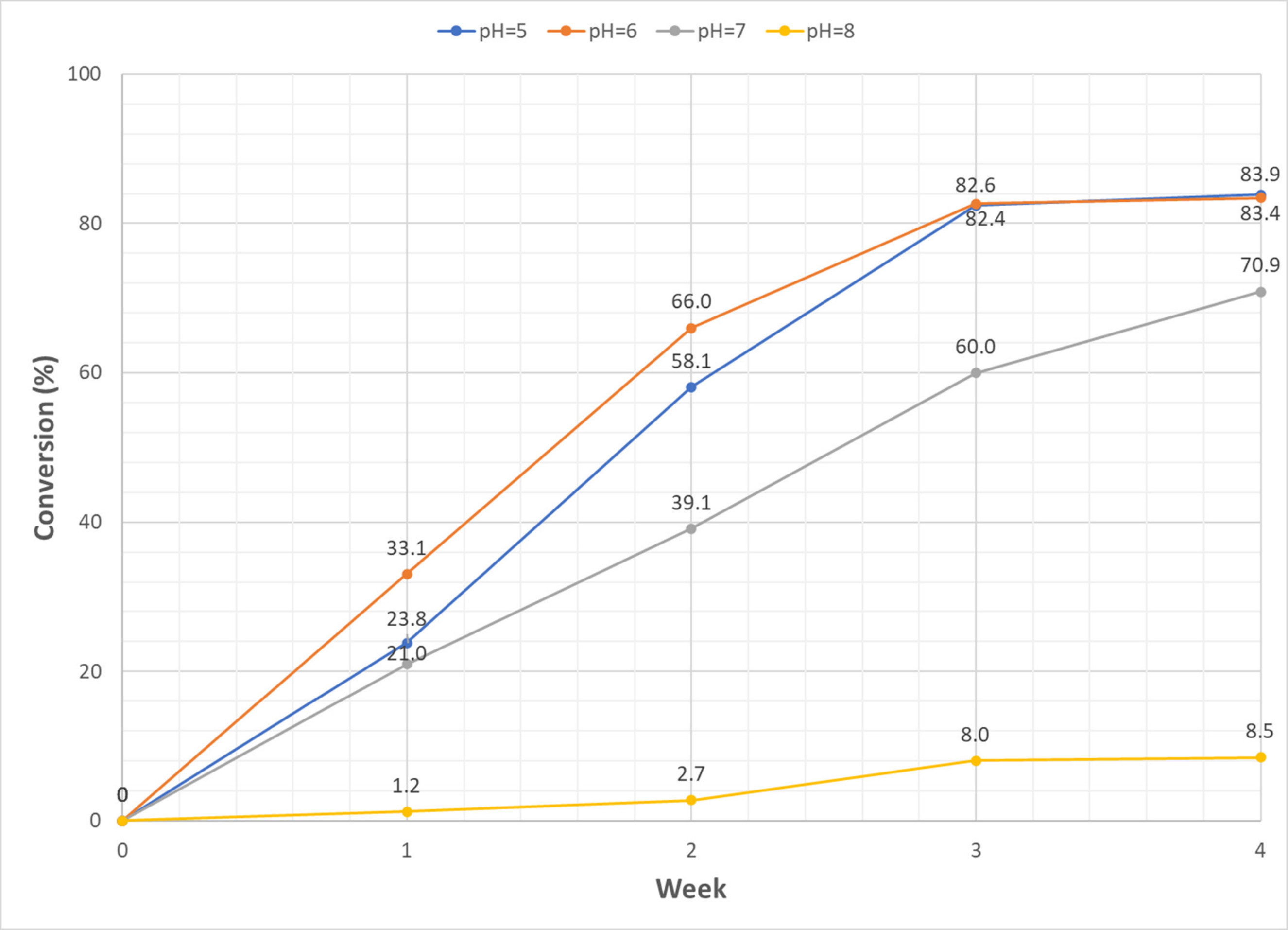

Figure S7. Percentage conversion to 2’,3’-cAMP as a function of time (weeks) at different pH with *imidazole* as activator.

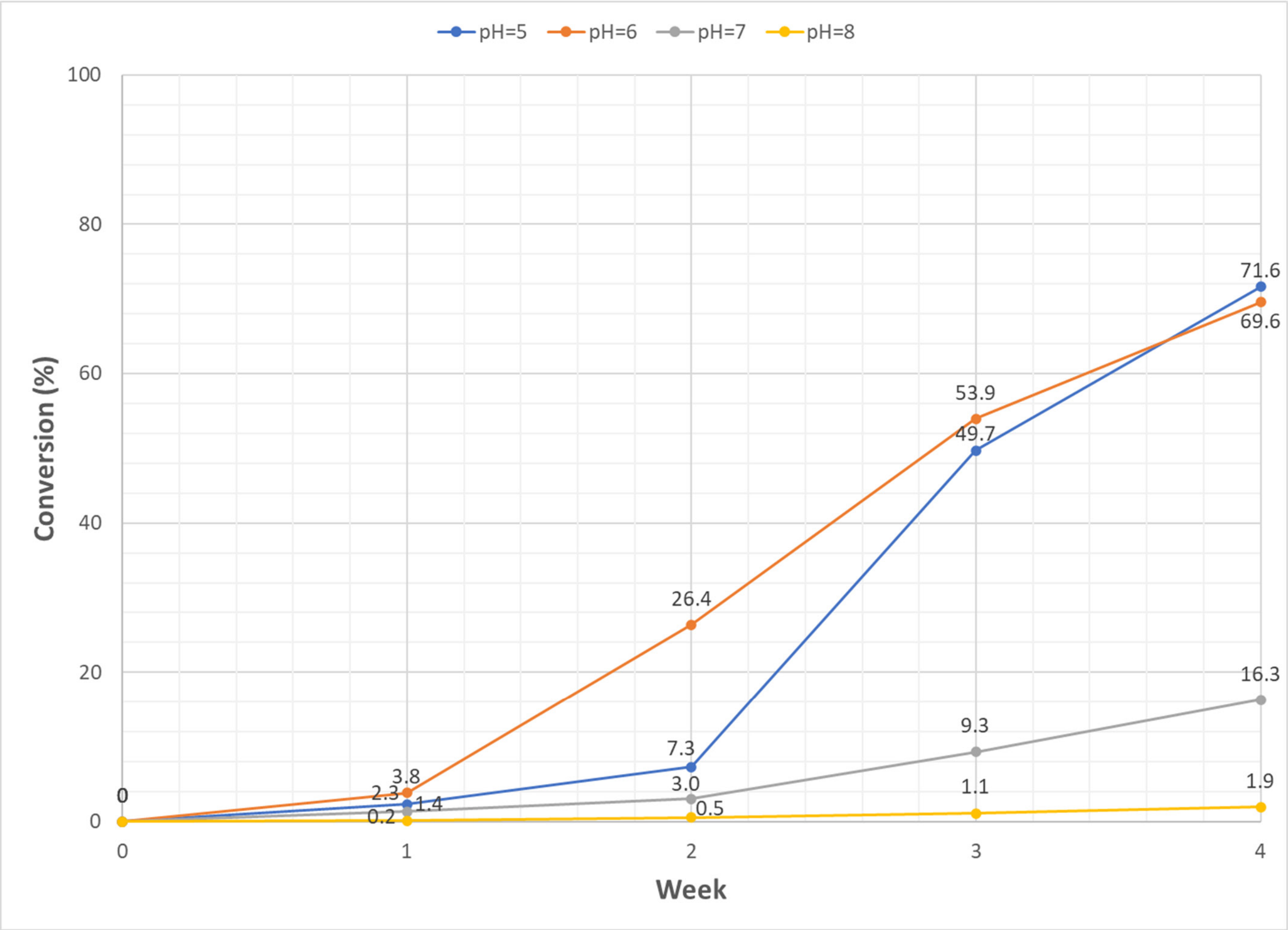

Figure S8. Percentage conversion to 2’,3’-cAMP as a function of time (weeks) at different pH with *2-methylimidazole* as activator.

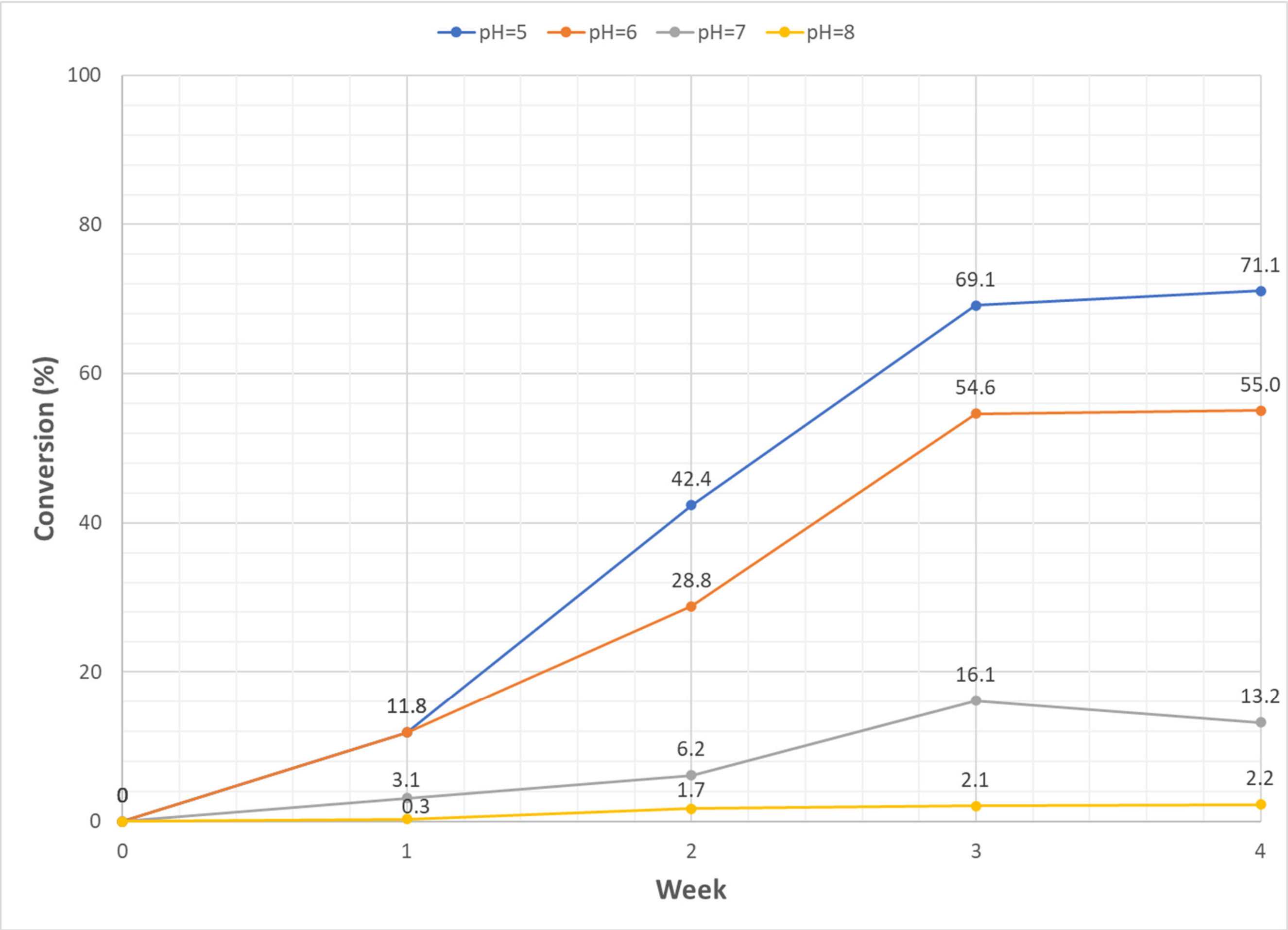

Figure S9. Percentage conversion to 2’,3’-cAMP as a function of time (weeks) at different pH with *2-aminoimidazole* as activator.

2.4 3'-GMP

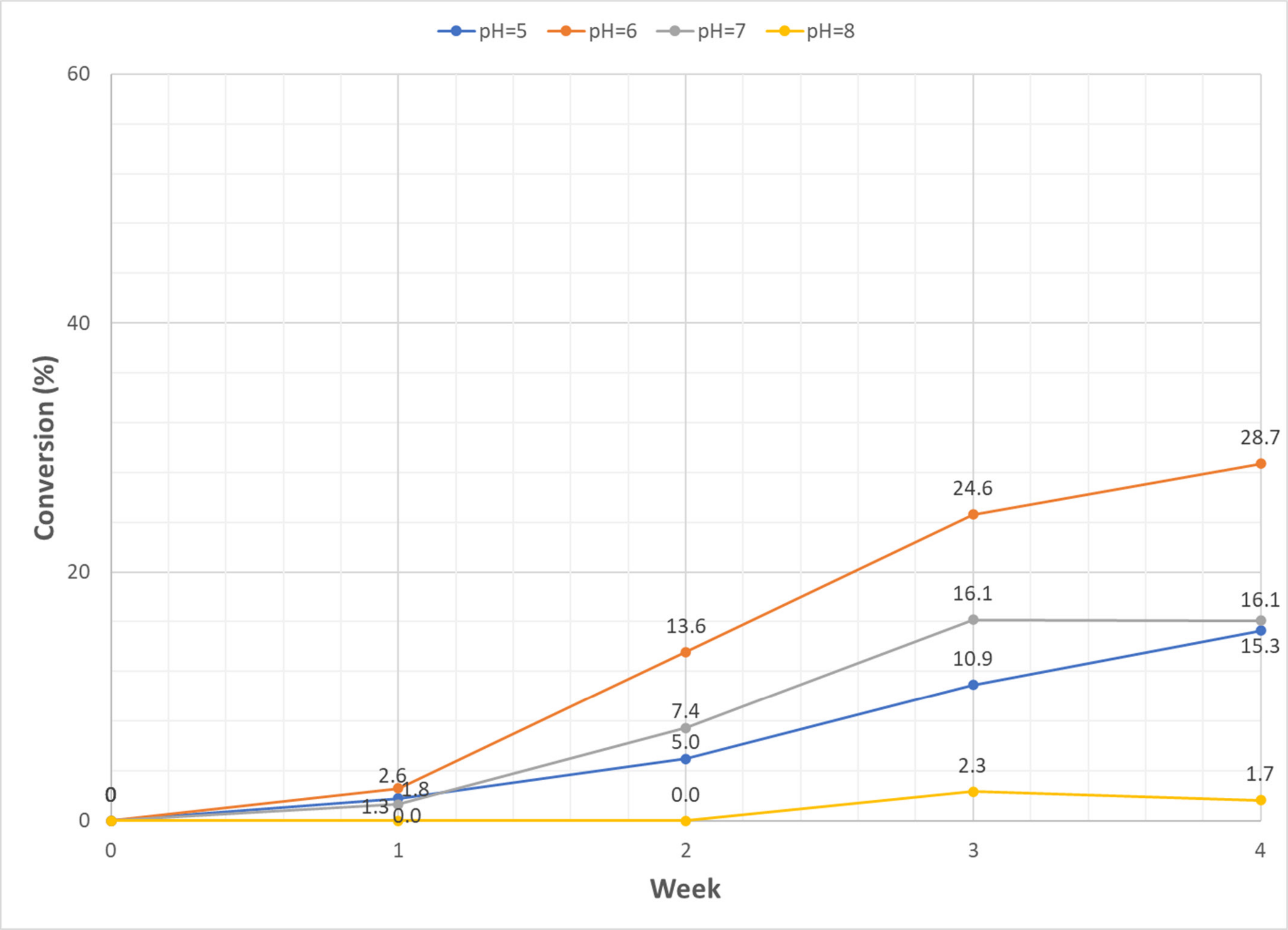

Figure S10. Percentage conversion to 2',3'-cGMP as a function of time (weeks) at different pH with *imidazole* as activator.

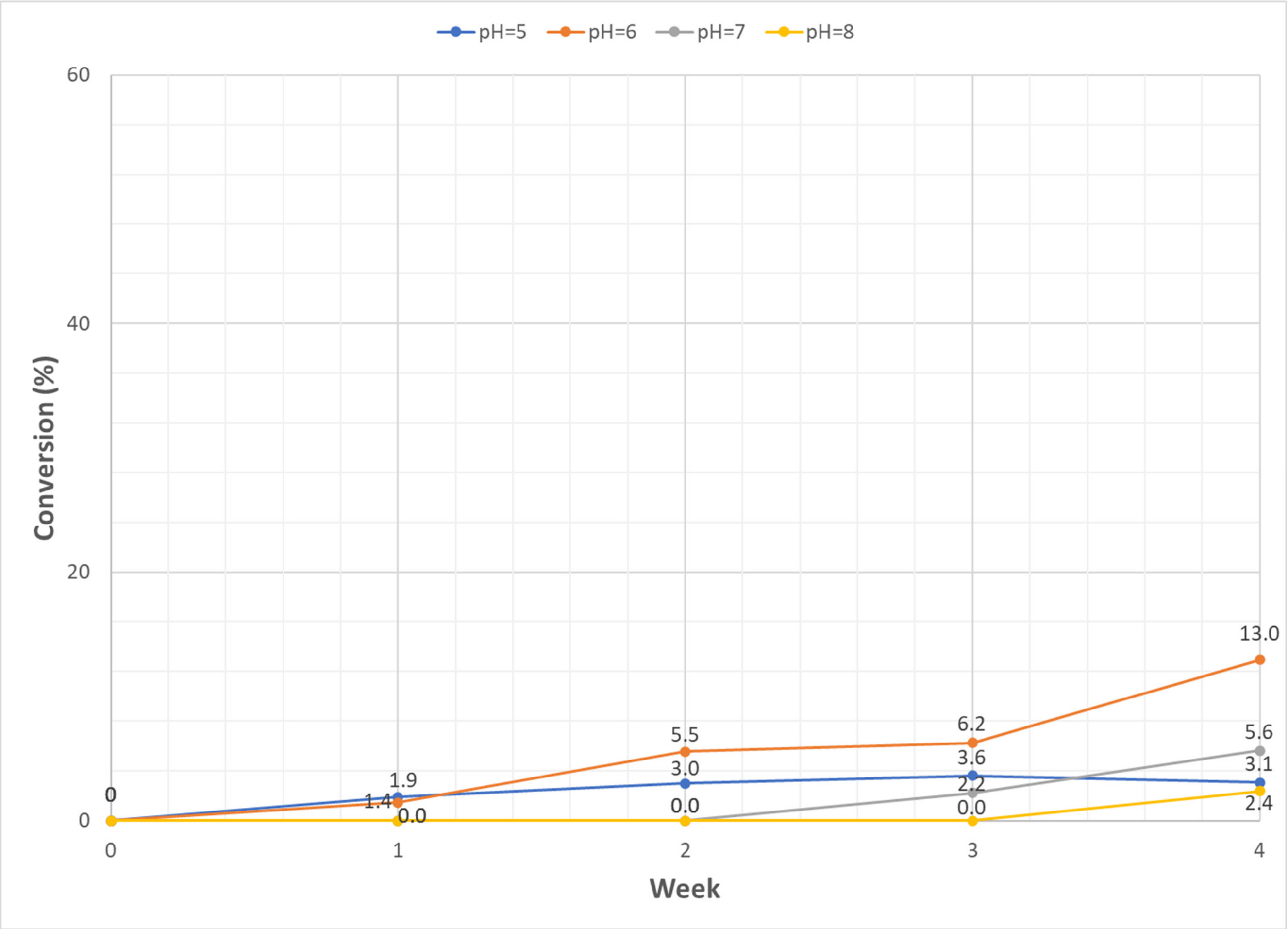

Figure S11. Percentage conversion to 2’,3’-cGMP as a function of time (weeks) at different pH with *2-methylimidazole* as activator.

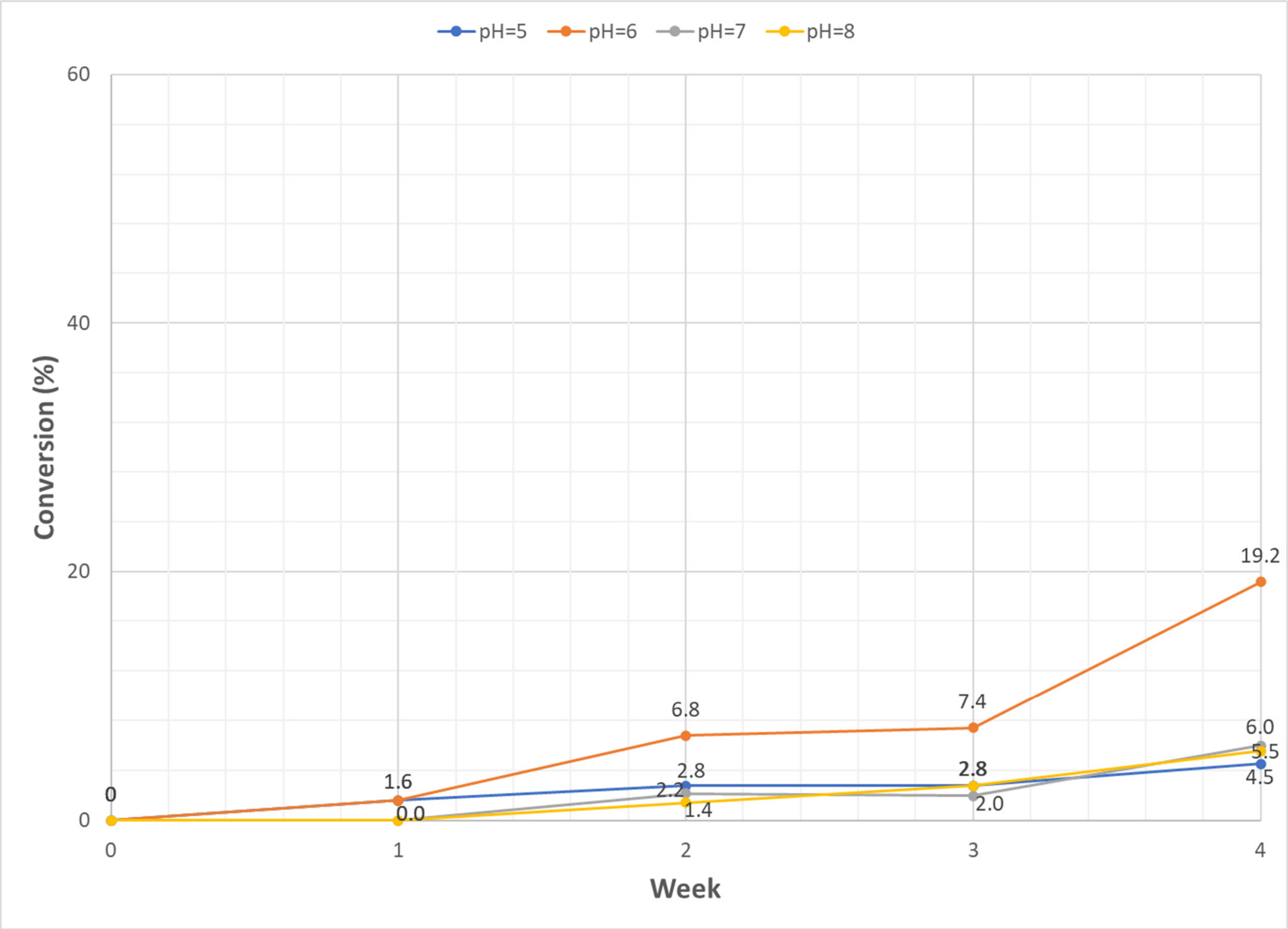

Figure S12. Percentage conversion to 2’,3’-cGMP as a function of time (weeks) at different pH with *2-aminoimidazole* as activator.

3. Comparison of activators at week 4

3.1 3’-UMP

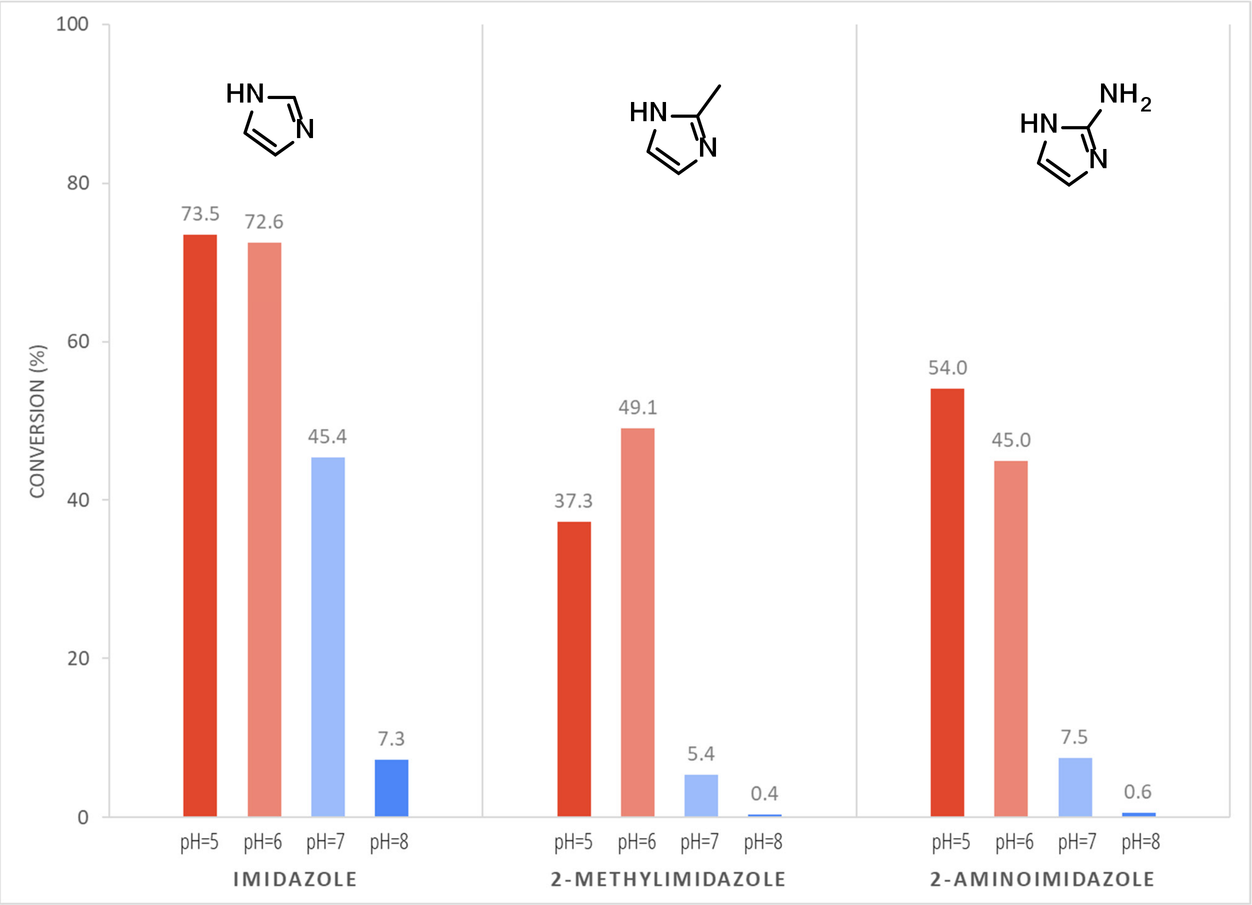

Figure S13. Effect of the activators on the % conversion to 2’,3’-cUMP at week 4.

3.2 3’-CMP

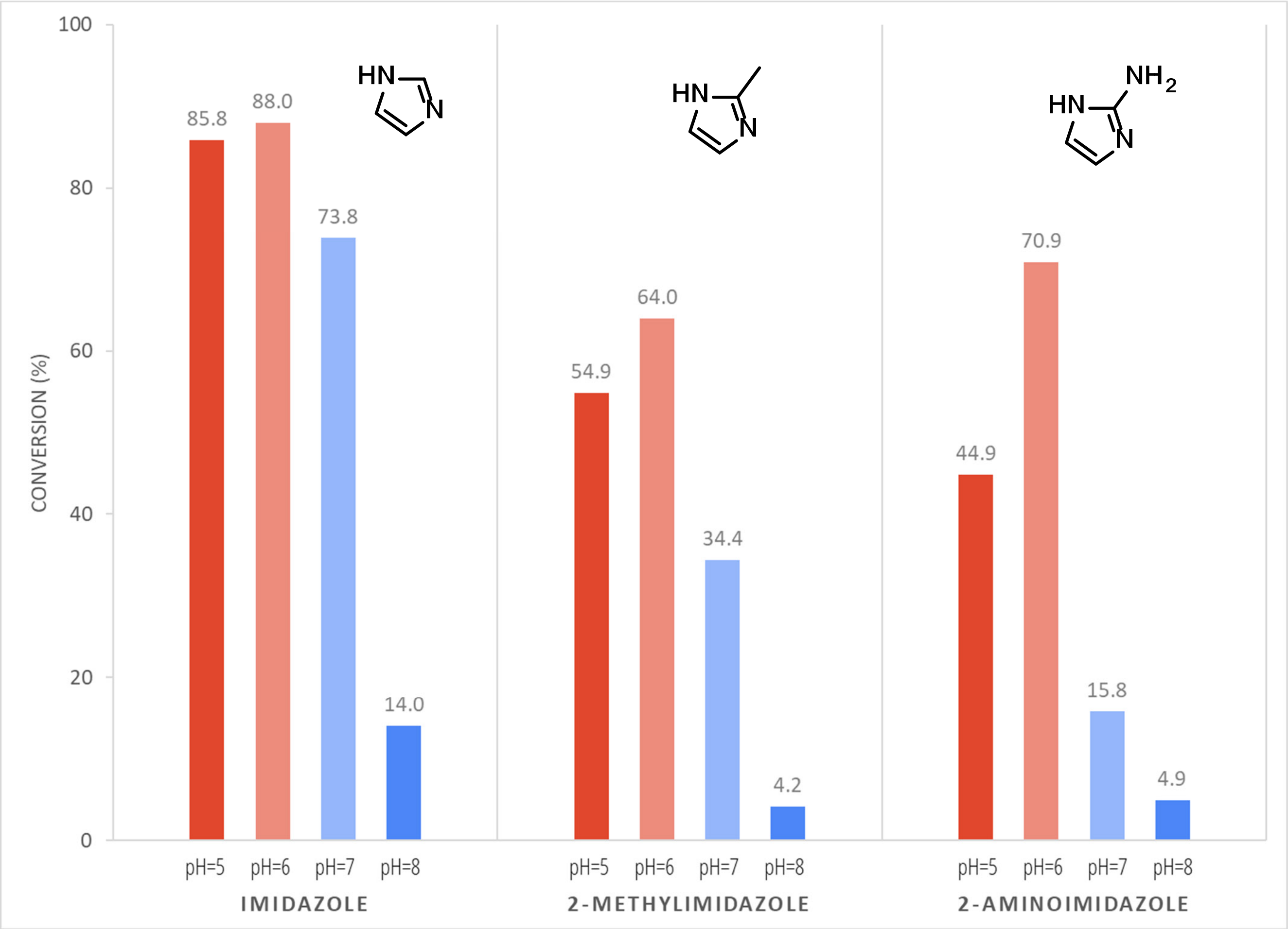

Figure S14. Effect of the activators on the % conversion to 2’,3’-cCMP at week 4.

3.3 3’-AMP

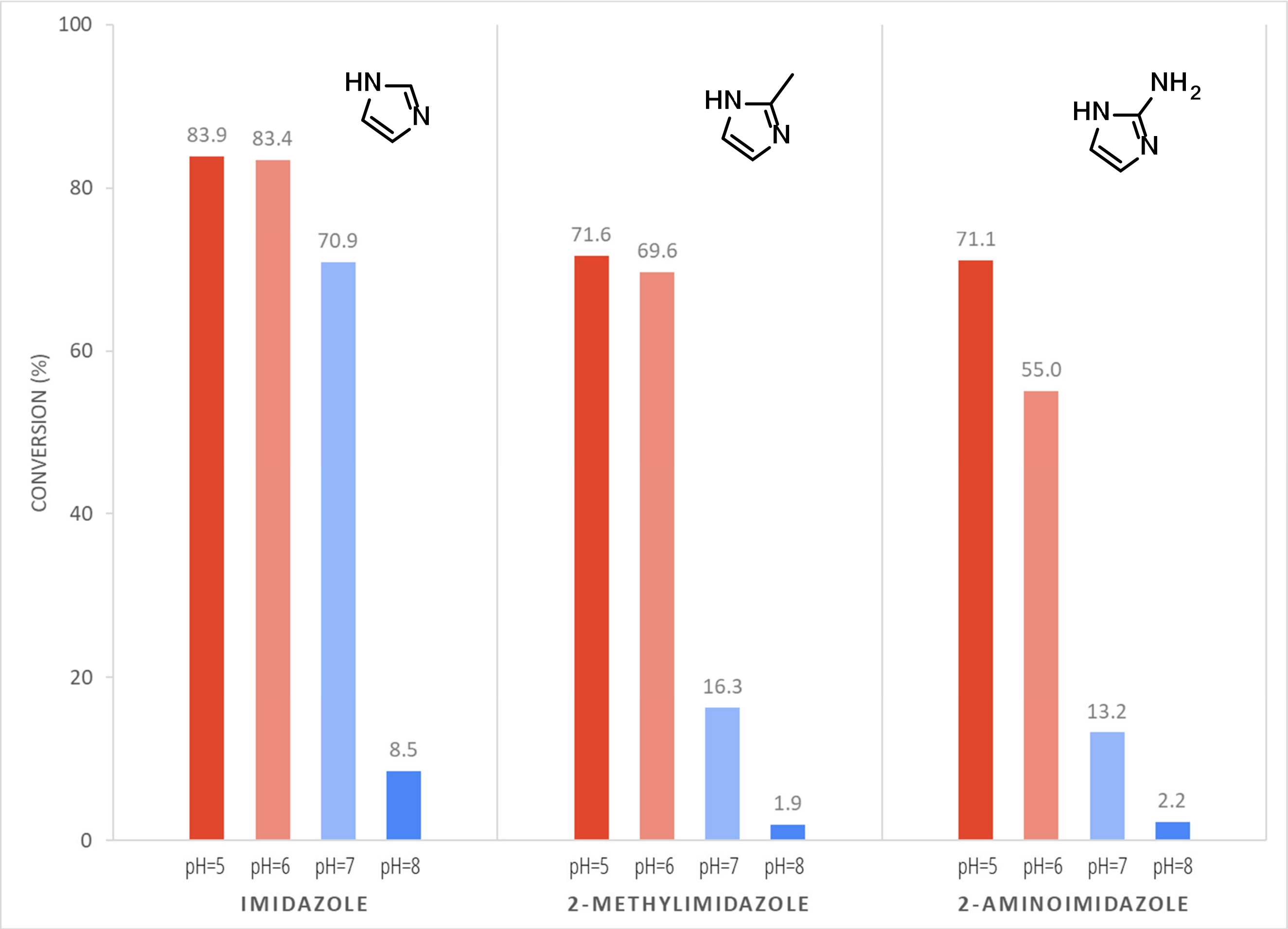

Figure S15. Effect of the activators on the % conversion to 2’,3’-cAMP at week 4.

3.4 3’-GMP

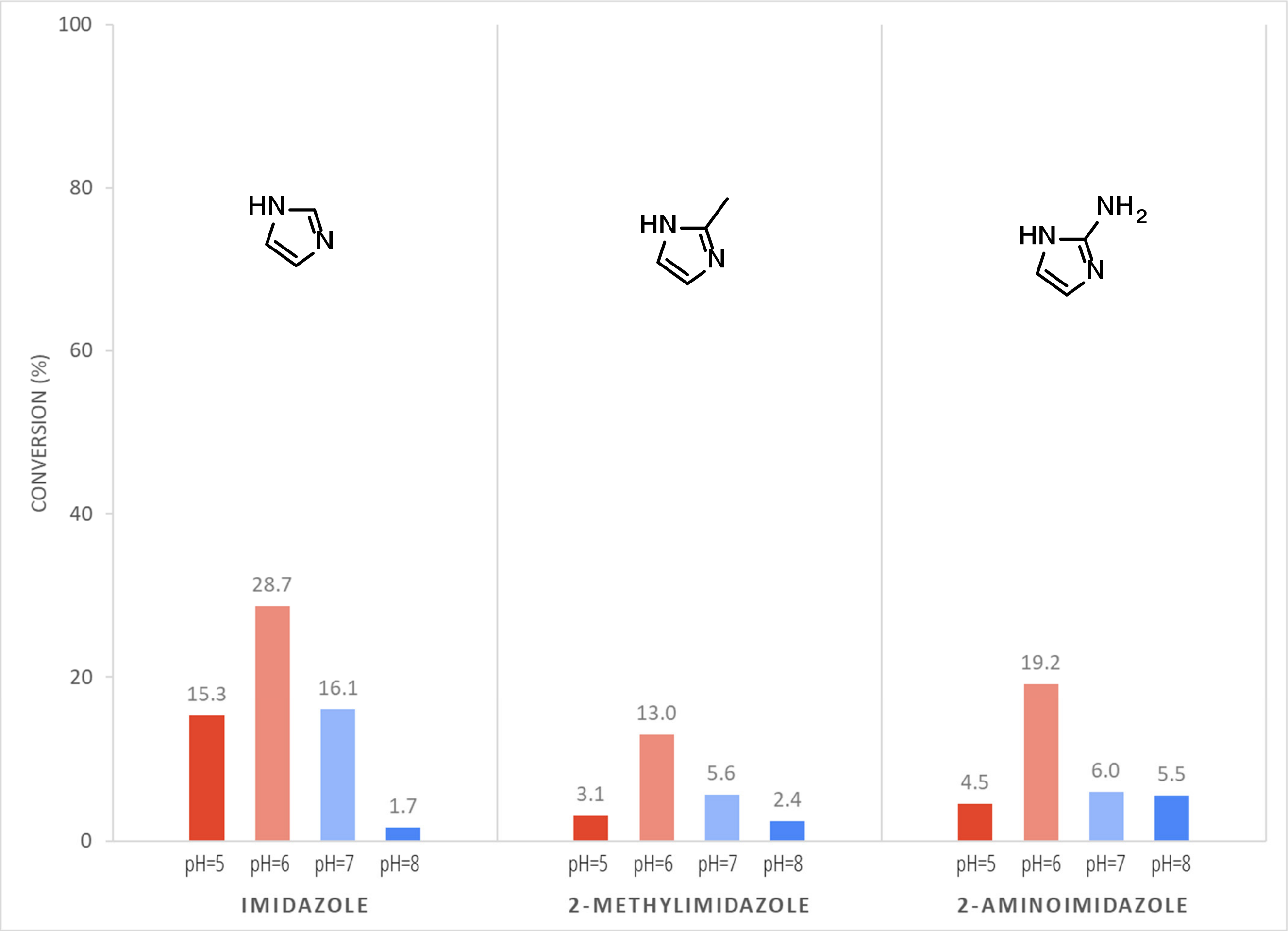

Figure S16. Effect of the activators on the % conversion to 2’,3’-cGMP at week 4.

4. General scheme of electrophoretic band shift assay

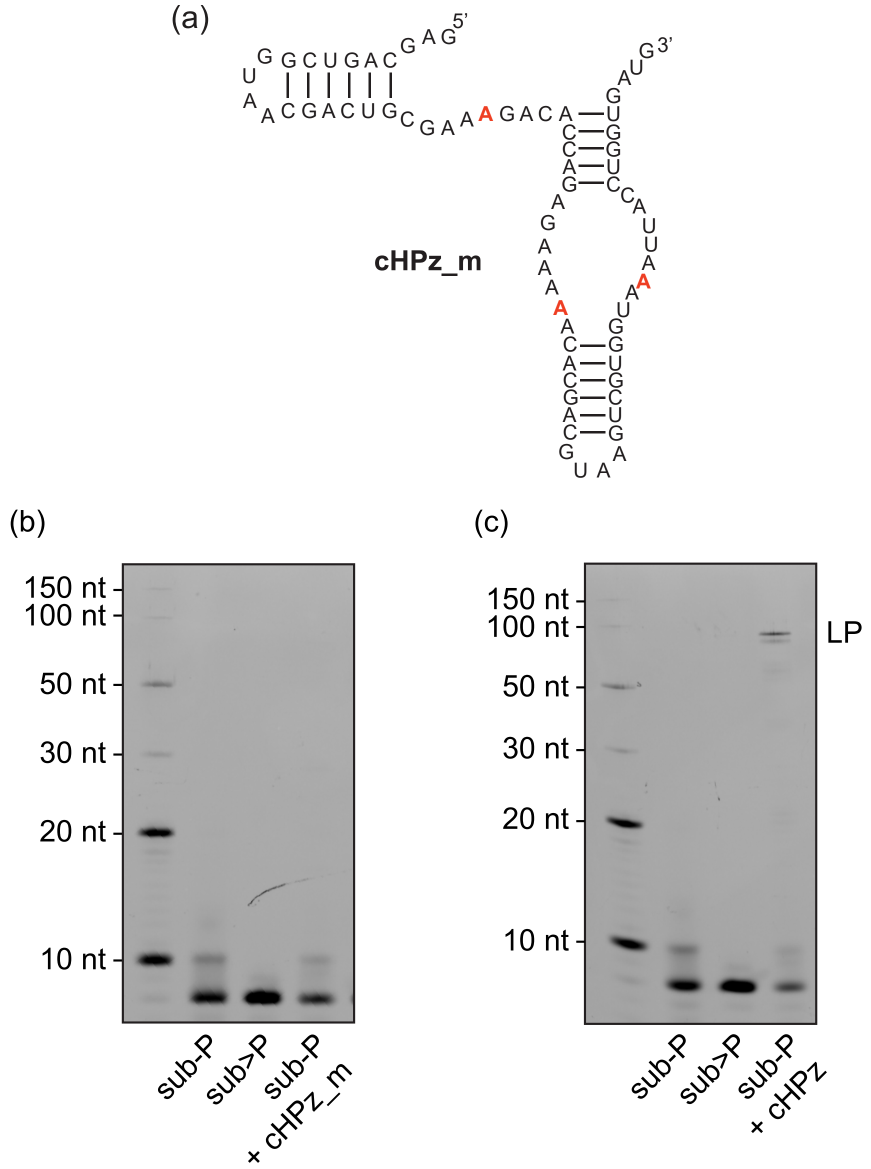

Figure S17. The modified hairpin ribozyme **cHPz** accepts only a cyclic 2',3'-phosphate substrate (**sub>P**) for its catalytic activity. (a) Secondary structure of **cHPz_m**, an inactive variant of **cHPz**. The mutated bases that abolish its catalytic activity are indicate in red. b) TBE-urea PA E analysis of ligation activity of **cHPz_m**. The reaction mixture (1 µM **sub-P** or **sub>P**, 10 µM **cHPz_m**, 25 mM NaCl, 10 mM pH 8.6 Tris· C buffer) were incubated under eutectic ice conditions for 4 days. The substrates **sub-P** and **sub>P** were 5' tagged with a fluorescein derivative to aid in the anal sis. (c) TBE-urea PAGE analysis ligation activity of cHPz. Uncropped version of gel shown in Figure 2b.

5. Ligation kinetics of cHPz

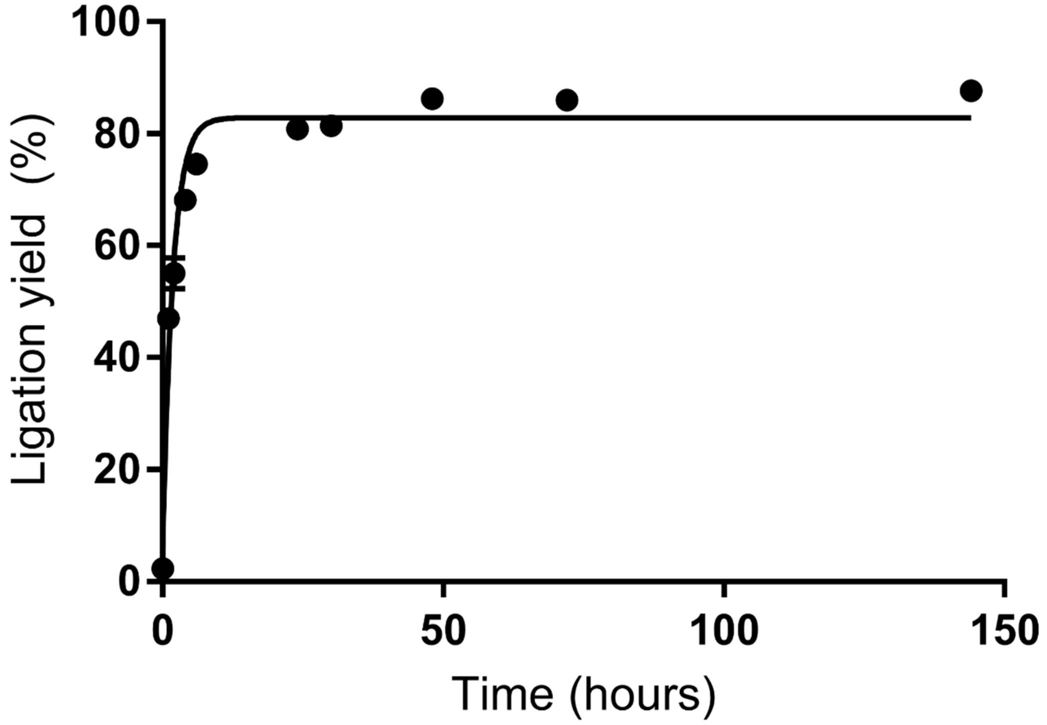

Figure S18. Reaction kinetics of the modified hairpin ribozyme **cHPz** under DAP activation conditions. 1 µM **sub>P** was incubated with 10 uM **cHPz** in activation buffer (5 mM DAP, 5 mM imidazole, 10 mM MES pH 6, 25 mM NaCl, 5 mM $MgCl_2$) in biological triplicates. The samples were analyzed via TBE-urea PAGE.

6. DAP-mediated ligation

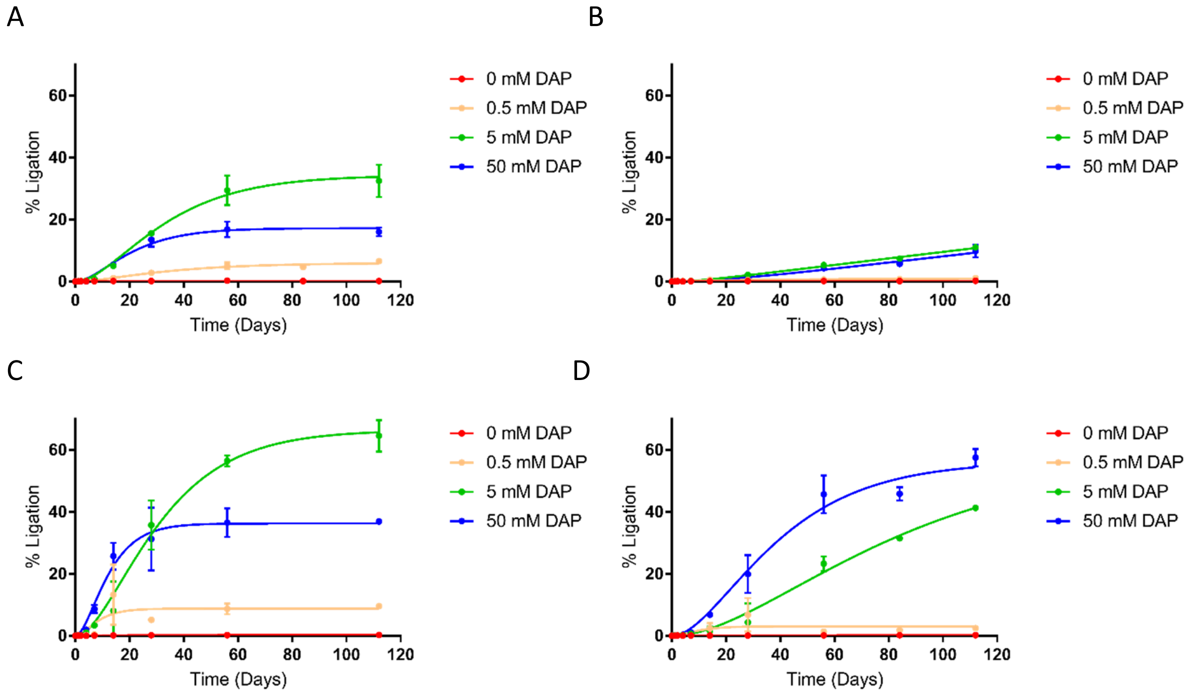

Figure S19. The DAP-mediated formation of 2’,3’-cyclic phosphate RNA and its subsequent ligation occur under various conditions compatible with ribozyme catalysis and is optimal at 5 mM DAP, 5 mM imidazole, and 5 mM $MgCl_2$. Time course analysis of *in situ* activation and ligation reactions performed under a range of conditions. Reaction mixtures (1 µM **sub-P**, 10 µM **cHPz**, 25 mM NaCl) were incubated under eutectic ice conditions in biological triplicates. A) 0.5 mM $MgCl_2$, 5 mM imidazole; B) 0.5 mM $MgCl_2$; C) 5 mM $MgCl_2$, 5 mM imidazole; D) 5 mM $MgCl_2$. The samples were analyzed via TBE-urea PAGE and the curves were fitted using a two-step reaction model.

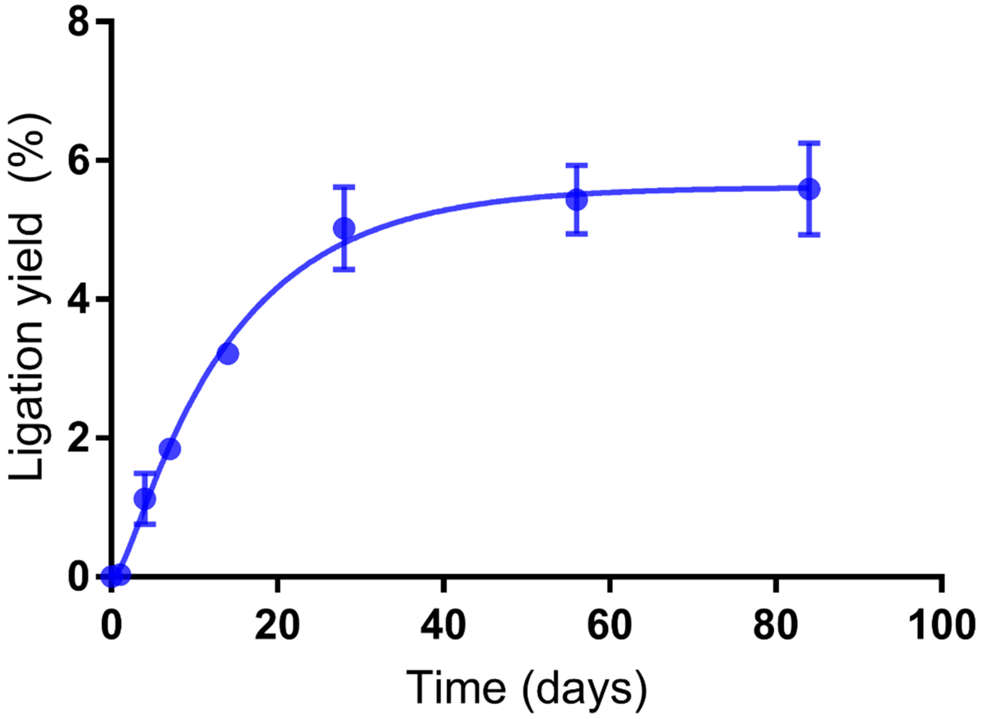

Figure S20. Increased concentrations of DAP and imidazole do not result in increased ligation yield under coupled DAP activation/ligation reaction conditions. Reaction mixtures (10 µM **sub-P**, 10 µM **cHPz**, 5 mM $MgCl_2$, 25 mM NaCl) containing 50 mM imidazole and 50 mM DAP were incubated under eutectic water-ice conditions in biological triplicates. The samples were analyzed via TBE-urea PAGE.

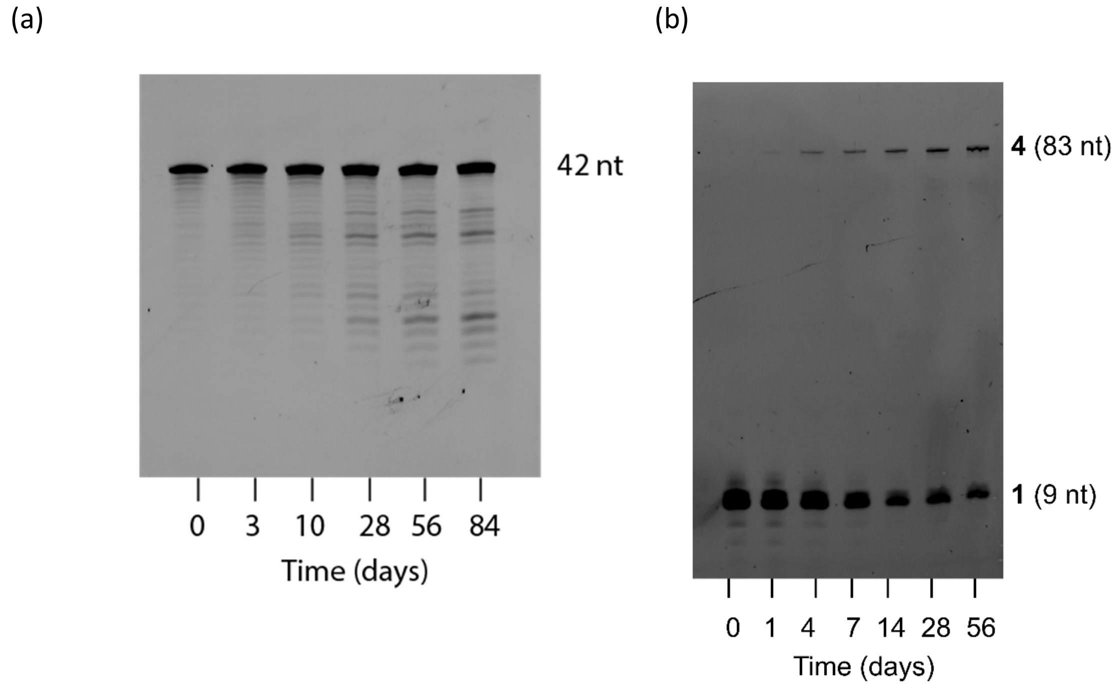

Figure S21. RNA stability during coupled activation-ligation reactions. (a) RNA degradation under standard reaction conditions is minimal even at ambient temperatures (1 µM RNA, 10 mM MES pH 6, 5 mM $MgCl_2$, 25 mM NaCl, 5 mM imidazole, ambient temperature). RNA degradation is expected to result in reverse laddering pattern of bands.[2] (b) Coupled DAP activation/cHPz ligation reaction time-course (Figure 3, pH = 6) TBE-urea PAGE analysis reveals no significant degradation of substrate RNA **sub-P** after 84 days. The apparent loss of overall fluorescence over time is attributed to the partially quenched fluorescence of **LP** and is further analyzed in Figure S24.

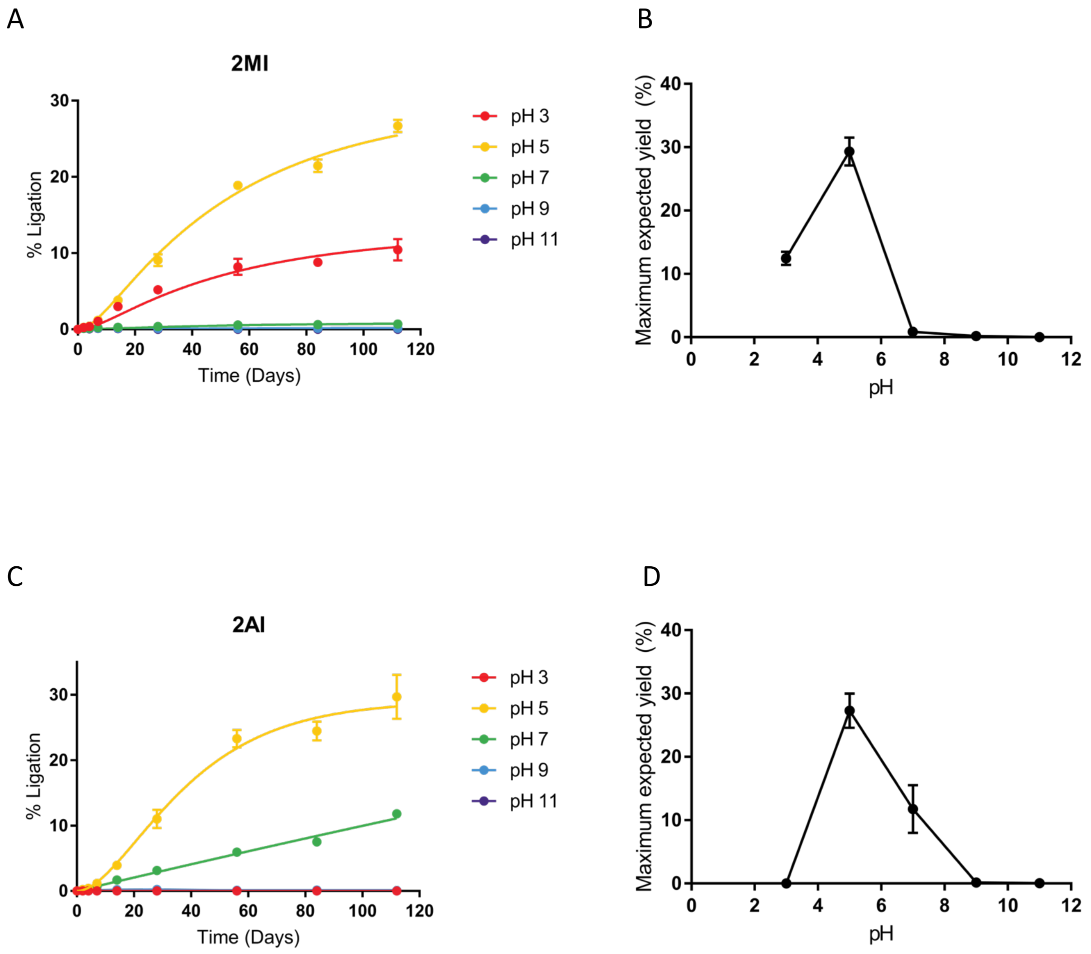

Figure S22. The DAP-mediated formation of 2′,3′-cyclic phosphate RNA and its subsequent ligation occur suboptimally with imidazole analogues. A) Time course analysis of *in situ* activation and ligation reactions performed at a range of different pH values and 5 mM 2-methylimidazole. B) pH dependency of the maximum ligation yield using the best-fit parameters from A. Error bars indicate the 95% confidence intervals of each fit parameter. C) Time-course analysis of *in situ* activation and ligation reactions performed under a range of pH and 5 mM 2-amimoimidazole. D) pH dependency of the maximum ligation yield using the best-fit parameters from C. Error bars indicate the 95% confidence intervals of each fit parameter. Reaction mixtures (1 µM **sub-P**, 10 µM **cHPz**, 5 mM DAP, 5 mM $MgCl_2$, 25 mM NaCl and 10 mM of either glycine·HCl (pH 3), MES (pH 5-7), Tris·HCl (pH 9), or carbonate/bicarbonate (pH 11) buffers were analyzed by TBE-urea PAGE. All time traces were fitted using equation 1.

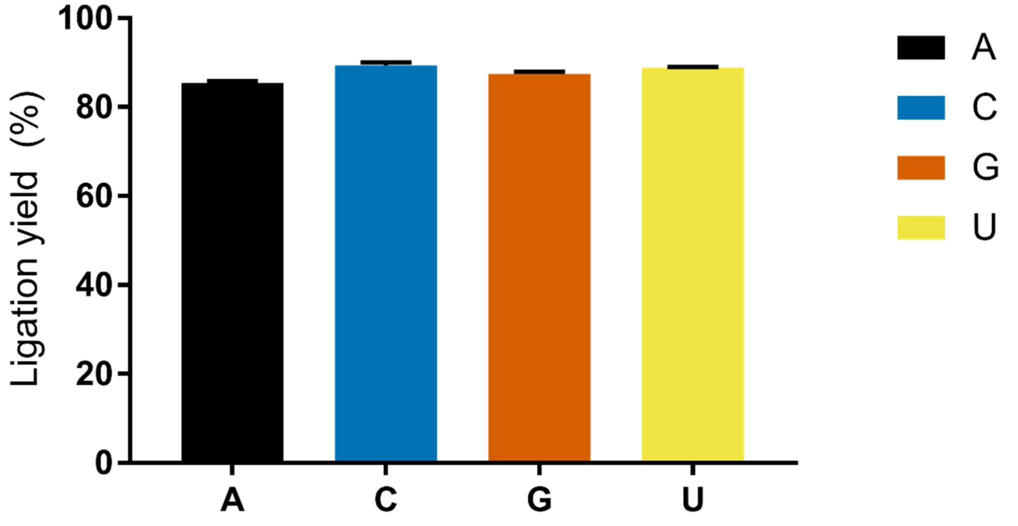

Figure S23. The 3’ base identity of the substrate RNA has a minimal effect on the ligation efficiency of cHPz under coupled DAP activation/ligation reaction conditions. Reaction mixtures (10 µM **cHPz,** 10 mM MES pH 6, 5 mM $MgCl_2$, 25 mM NaCl, 5 mM imidazole, 5 mM DAP) containing 1 µM of EDC pre-activated RNA substrates differing only at the 3’ end were incubated under eutectic water-ice conditions for 45 hr in biological triplicates. The samples were analyzed via TBE-urea PAGE.

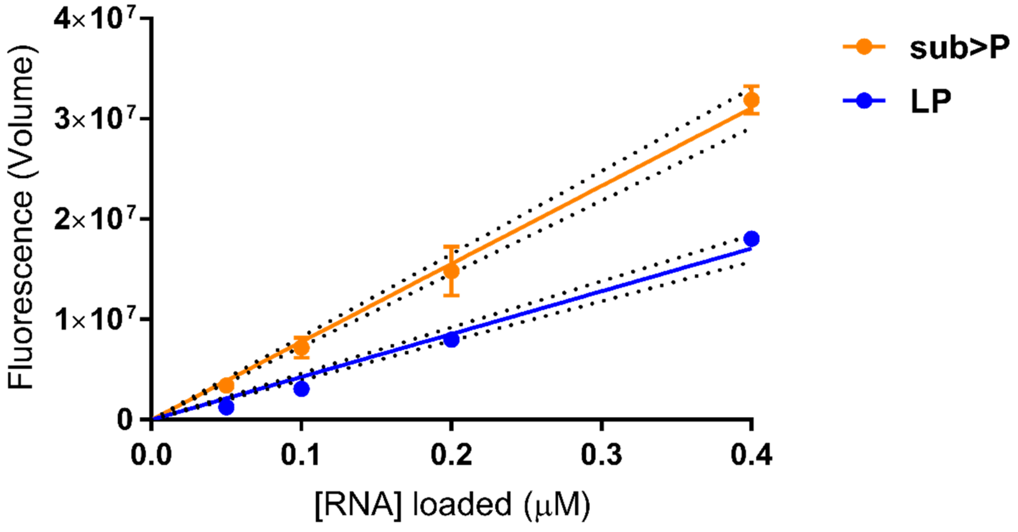

Figure S24. Calculating the fluorescence correction factor for **LP**. Fluorescence calibration curves for **sub>P** and **LP** were generated by quantifying various concentrations of triplicate samples analyzed on a single TBE-urea PAGE. The average difference between the slopes, 1.82, was applied as a correction factor to the fluorescence of **LP** in all quantification of activation/ligation yields presented herein. Dotted lines indicate the 95% confidence intervals.

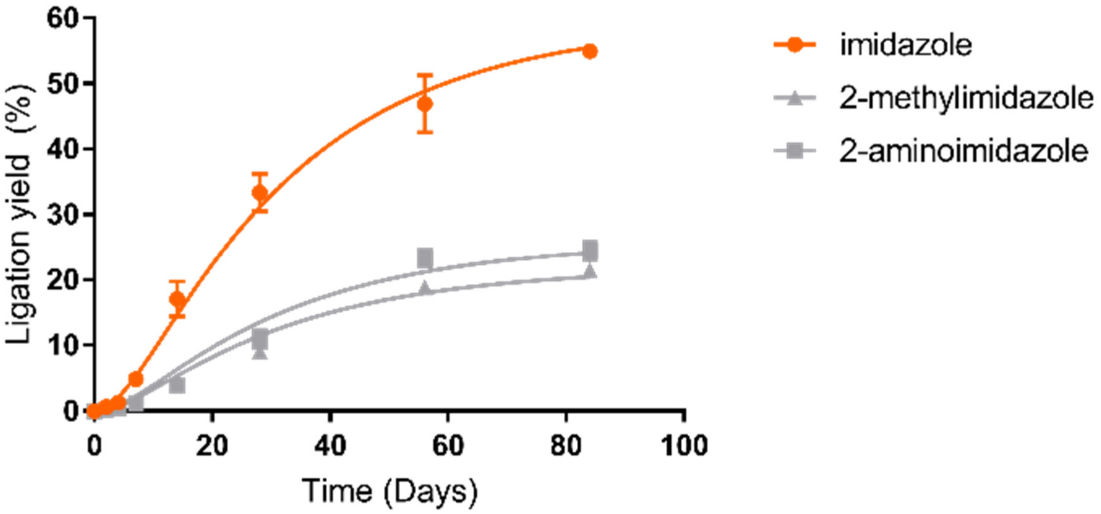

Figure S25. Comparison of different imidazole derivatives as DAP-activating agents at pH = 5. Coupled activation-ligation reactions were performed using imidazole and two of its analogues, 2-methylimidazole (2MI) and 2-aminoimidazole (2AI), and analysed via TBE-urea PAGE. Data points were fitted assuming a simplified 2-step reaction mechanism.

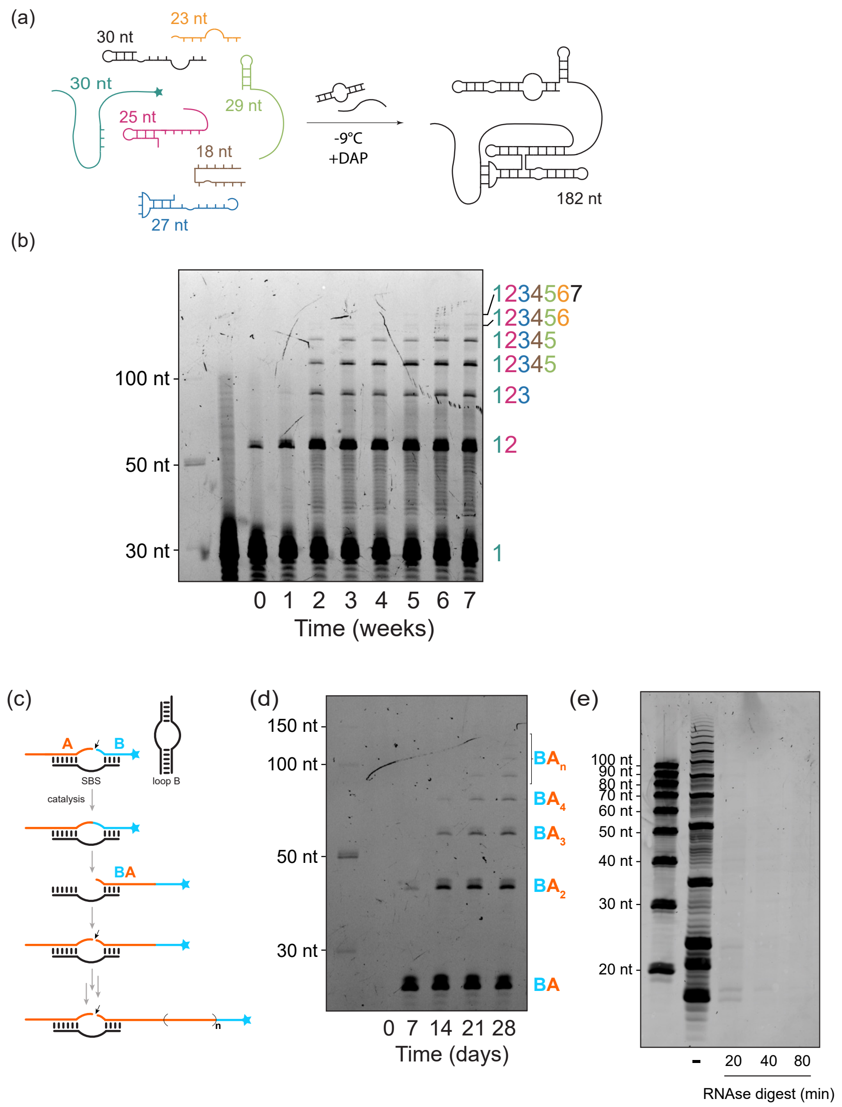

Figure S26. DAP-mediated assembly of RNA polymerase ribozyme 7 (RPR7) from <30 nt oligomers (a, b) and of concatemers (c, d). (a) Simplified schematic of RPR7 assembly. Adapted from Mutschler *et al.* (2015).[1] (b) Time course analysis via unstained TBE-urea PAGE of reaction mixtures containing seven 2'/3'-monophophorylated fragments of RPR7, 25 mM NaCl, 5 mM imidazole, 5 mM DAP, 5 mM MgCl2, and 10 mM pH 6 MES buffer. Fragment concentrations ranged from 0.5 µM (fragment 1, tagged with fluorophore) to 0.9 µM (fragment 7). The frozen reactions were subjected to incubation at -9 °C then to freeze-thaw cycling conditions after 14 days. (c,d) Alternative visualisation of data presented in Figure 4bc. B is tagged with a fluorophore. Time course analysis via unstained TBE-urea PAGE. We note that the RNA ladder displays lower electrophoretic mobility, presumably due to different salt concentrations in the loading buffer and / or conjugated fluorophores. (e) RNAse R digestion analysis via SYBR Gold-stained TBE-urea PAGE of concatenation samples reveals absence of circularized products.

7. IE-LC chromatograms for comparisons of activators

7.1 Chromatograms 3’-UMP → 2’,3’-cUMP with different activators (weeks 1 to 4)

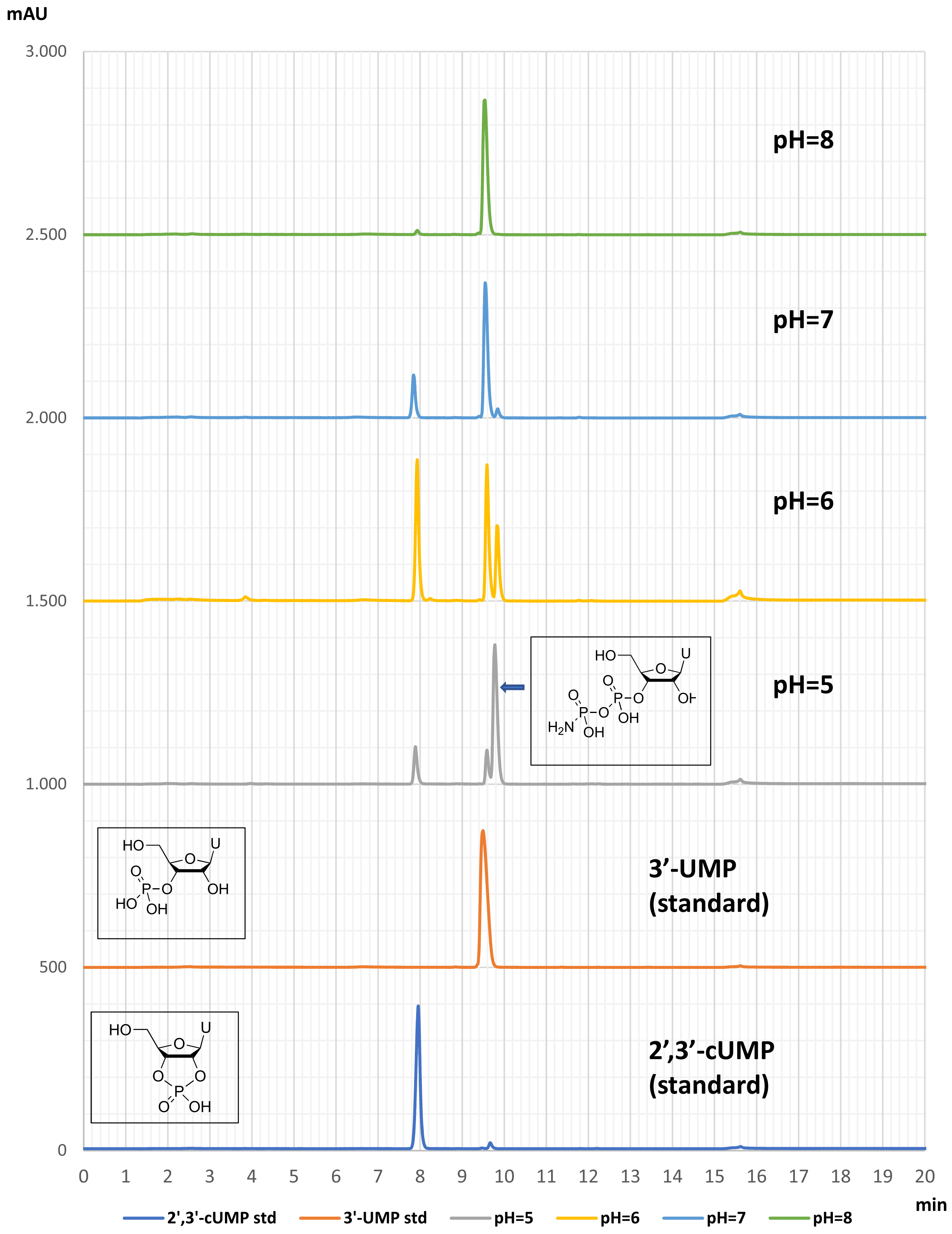

Figure S27. Ion exchange chromatograms of the effect of the pH of a reaction mixture of 3’-UMP (1 µmol, 1eq), DAP (5eq), $MgCl_2$ (5eq) and **imidazole** (5eq) in 1mL of water at -20°C (*week 1*).

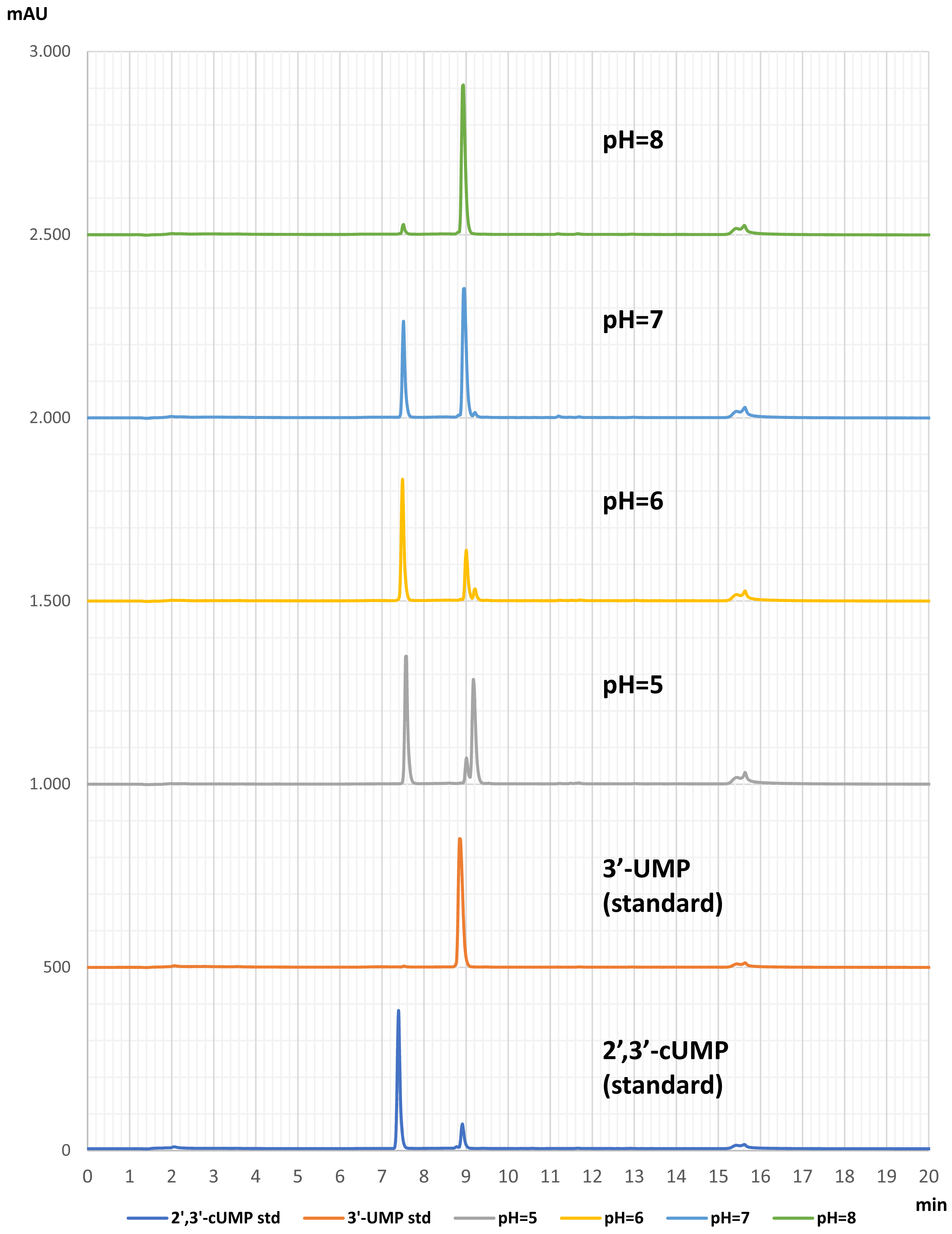

Figure S28. Ion exchange chromatograms of the effect of the pH of a reaction mixture of 3’-UMP (1 µmol, 1eq), DAP (5eq), $MgCl_2$ (5eq) and **imidazole** (5eq) in 1mL of water at -20°C (*week 2*).

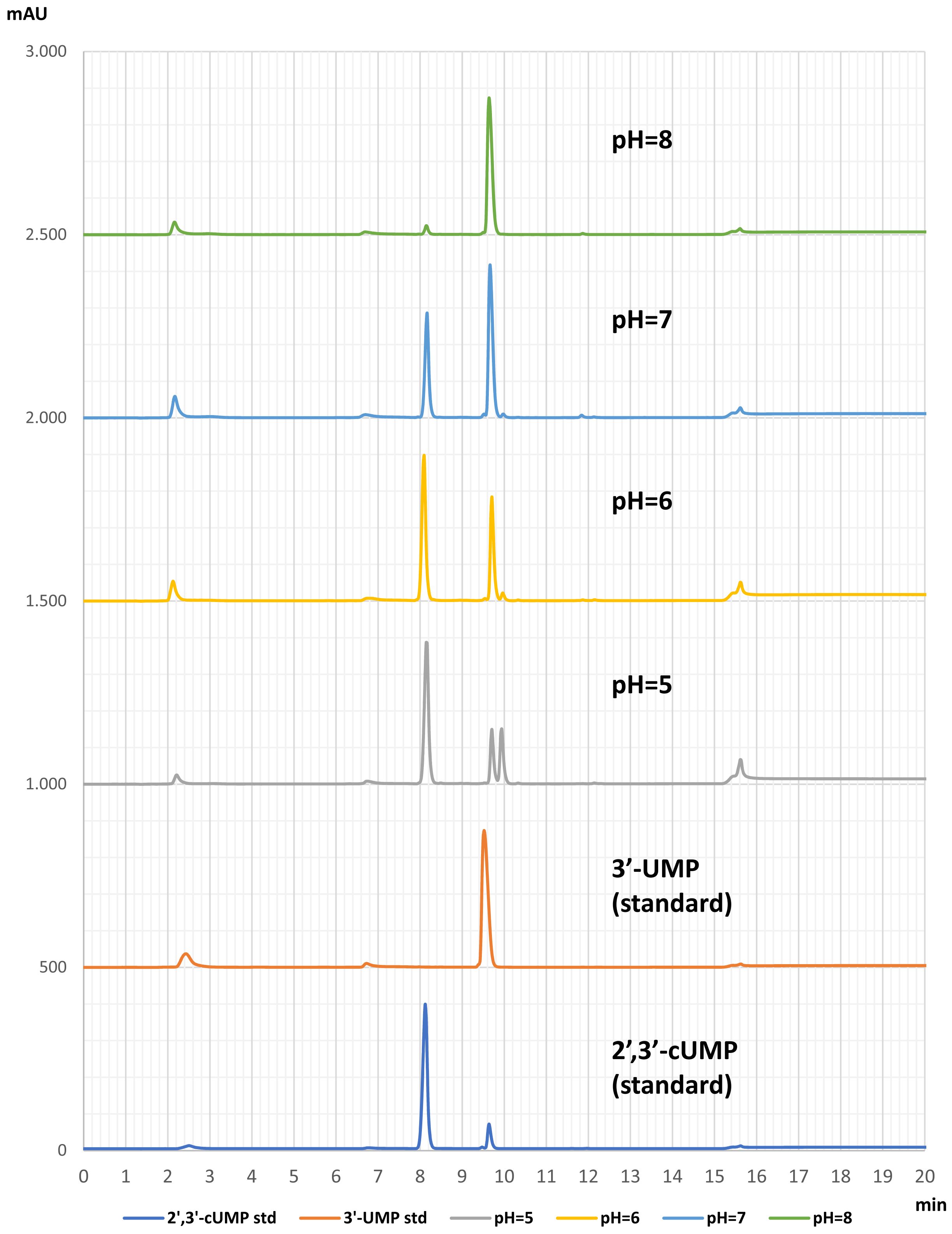

Figure S29. Ion exchange chromatograms of the effect of the pH of a reaction mixture of 3'-UMP (1 µmol, 1eq), DAP (5eq), $MgCl_2$ (5eq) and **imidazole** (5eq) in 1mL of water at -20°C (*week 3*).

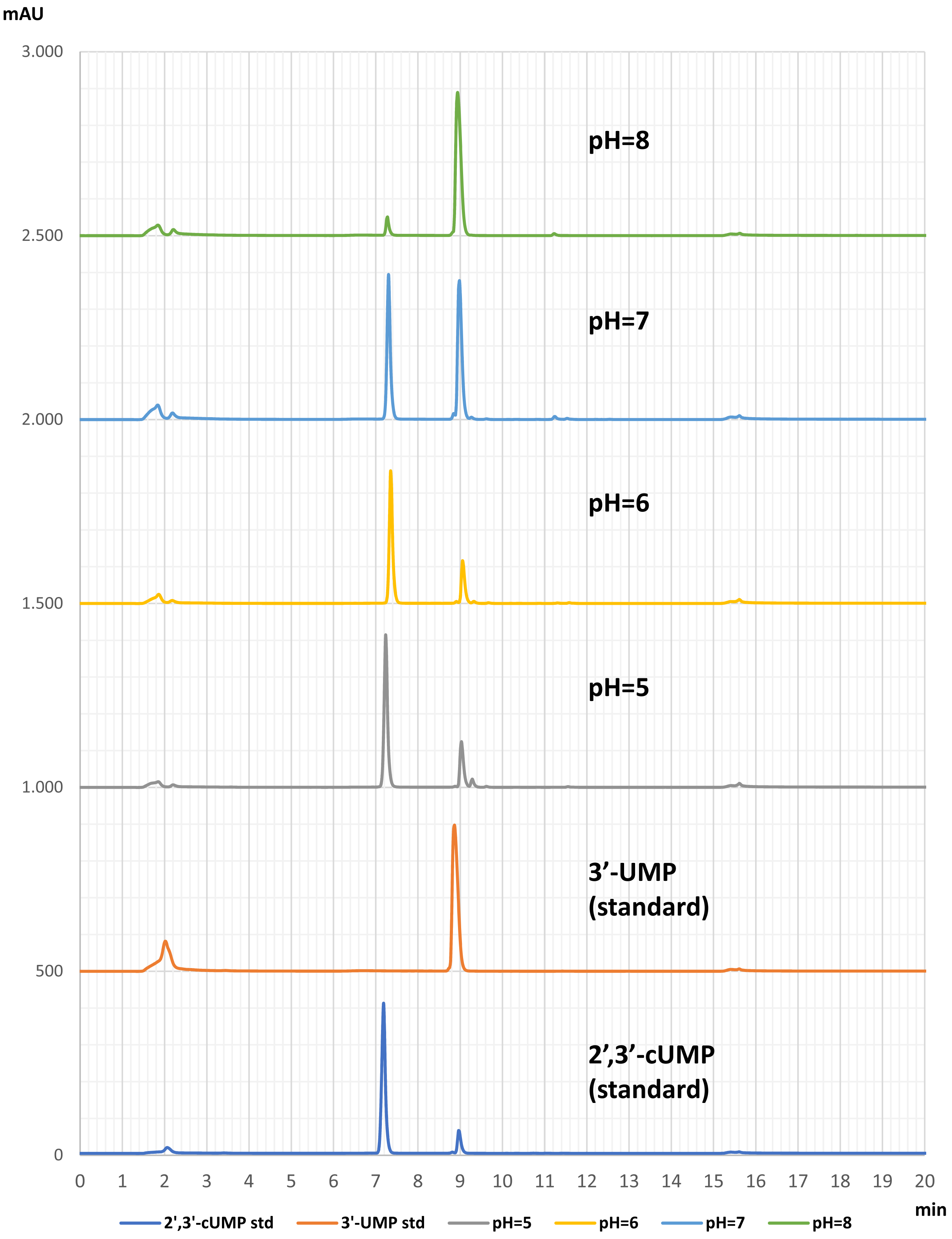

Figure S30. Ion exchange chromatograms of the effect of the pH of a reaction mixture of 3’-UMP (1 µmol, 1eq), DAP (5eq), $MgCl_2$ (5eq) and **imidazole** (5eq) in 1mL of water at -20°C (*week 4*).

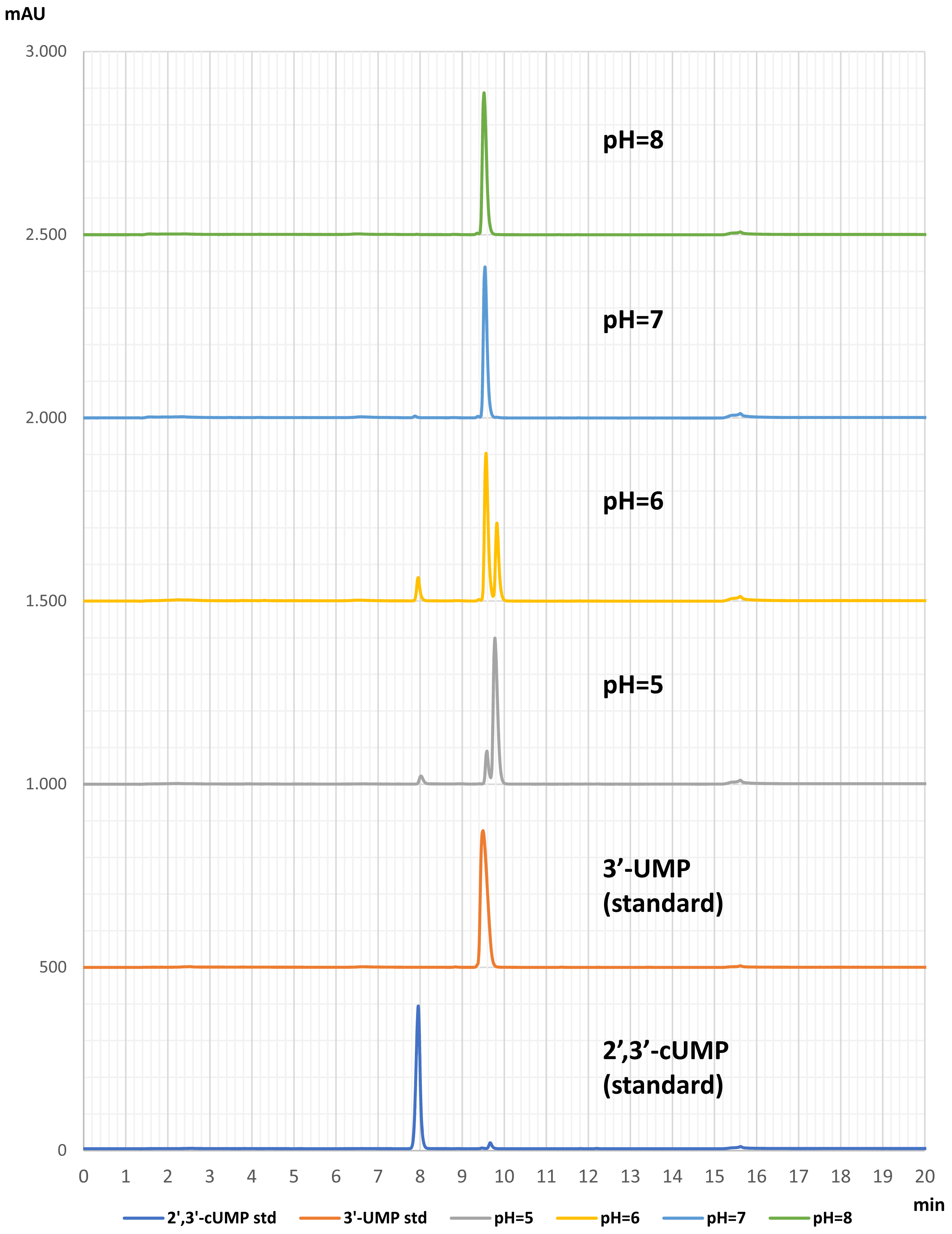

Figure S31. Ion exchange chromatograms of the effect of the pH of a reaction mixture of 3’-UMP (1 µmol, 1eq), DAP (5eq), $MgCl_2$ (5eq) and **2-methylimidazole** (5eq) in 1mL of water at -20°C (*week 1*).

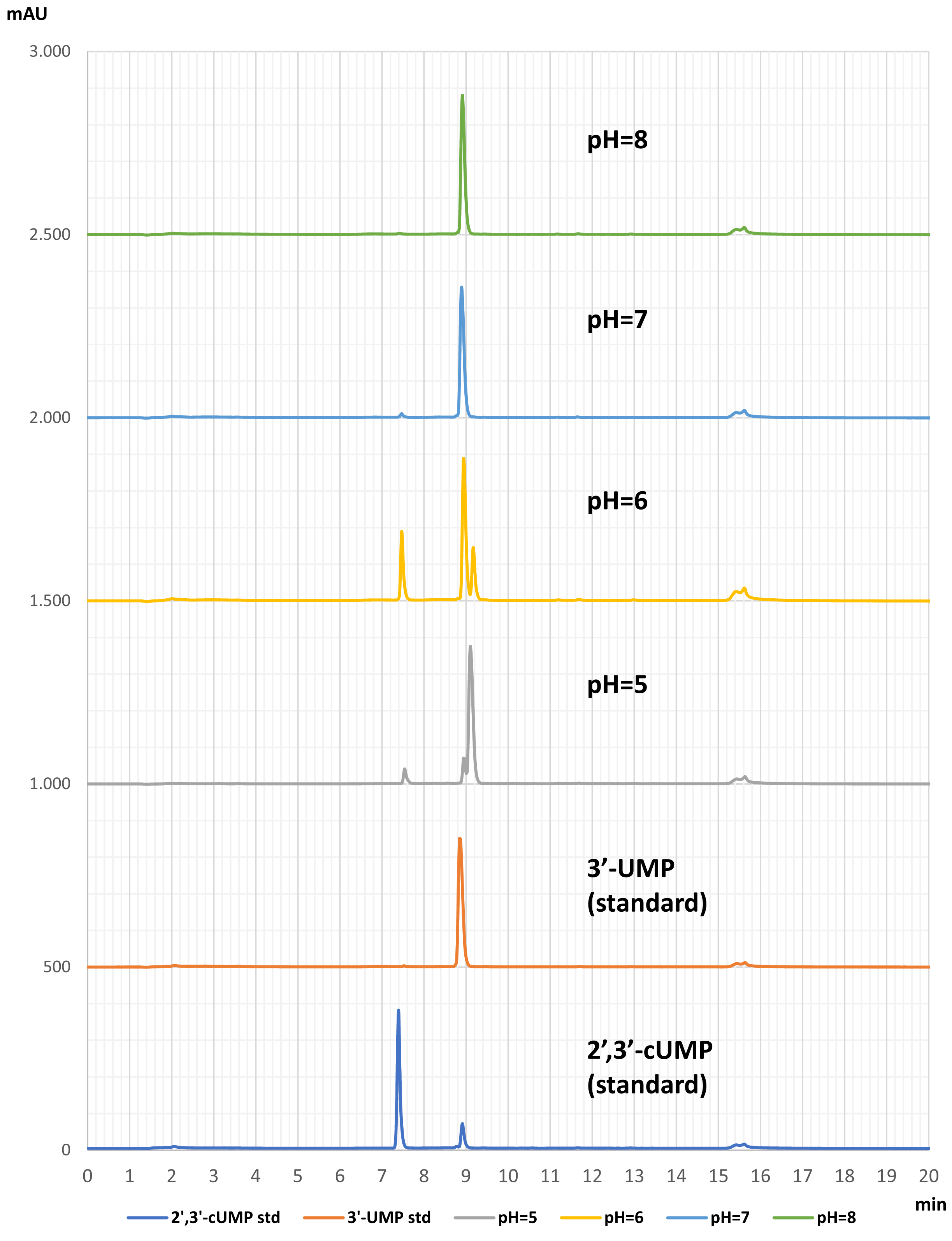

Figure S32. Ion exchange chromatograms of the effect of the pH of a reaction mixture of 3’-UMP (1 µmol, 1eq), DAP (5eq), $MgCl_2$ (5eq) and **2-methylimidazole** (5eq) in 1mL of water at -20°C (*week 2*).

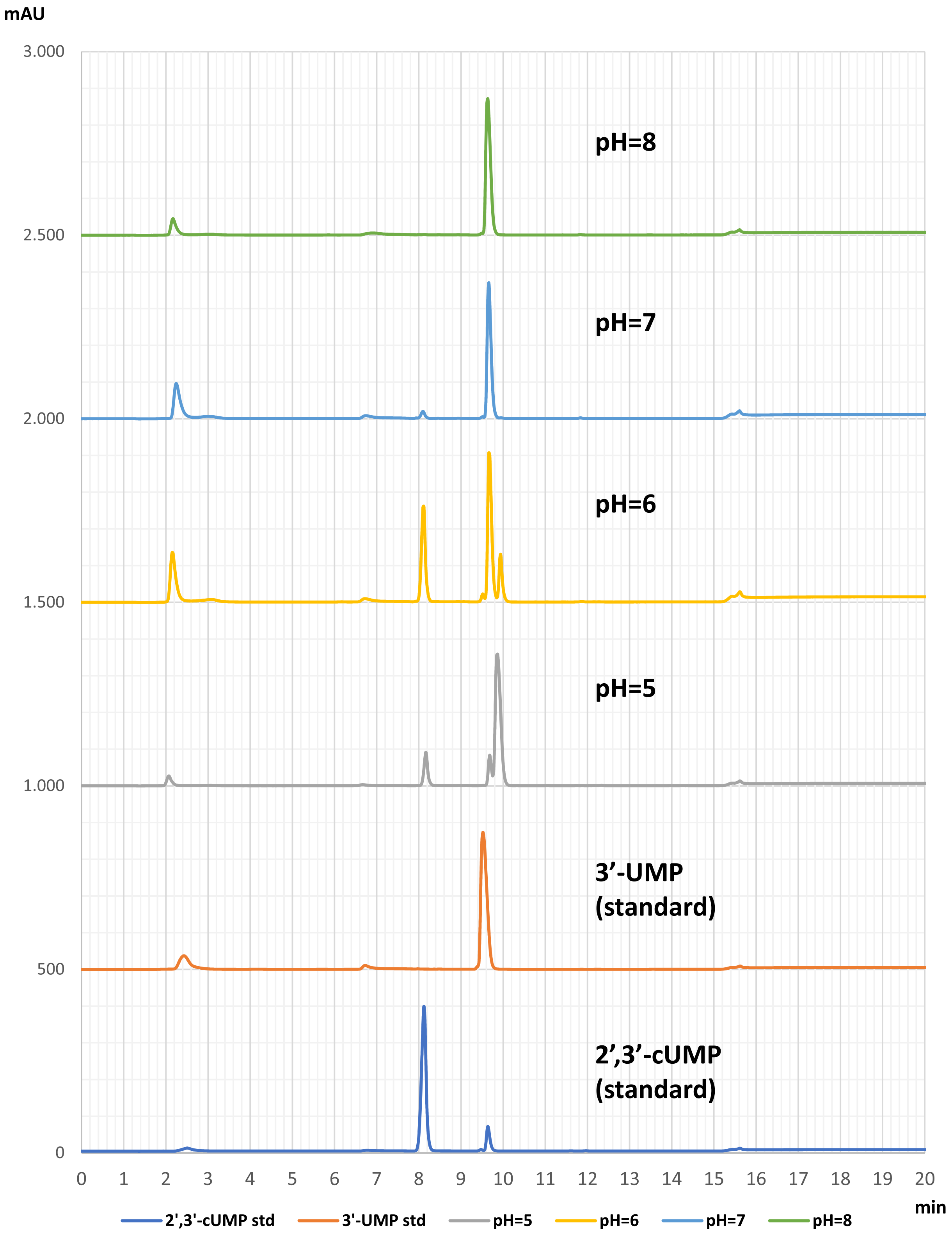

Figure S33. Ion exchange chromatograms of the effect of the pH of a reaction mixture of 3’-UMP (1 µmol, 1eq), DAP (5eq), $MgCl_2$ (5eq) and **2-methylimidazole** (5eq) in 1mL of water at -20°C (*week 3*).

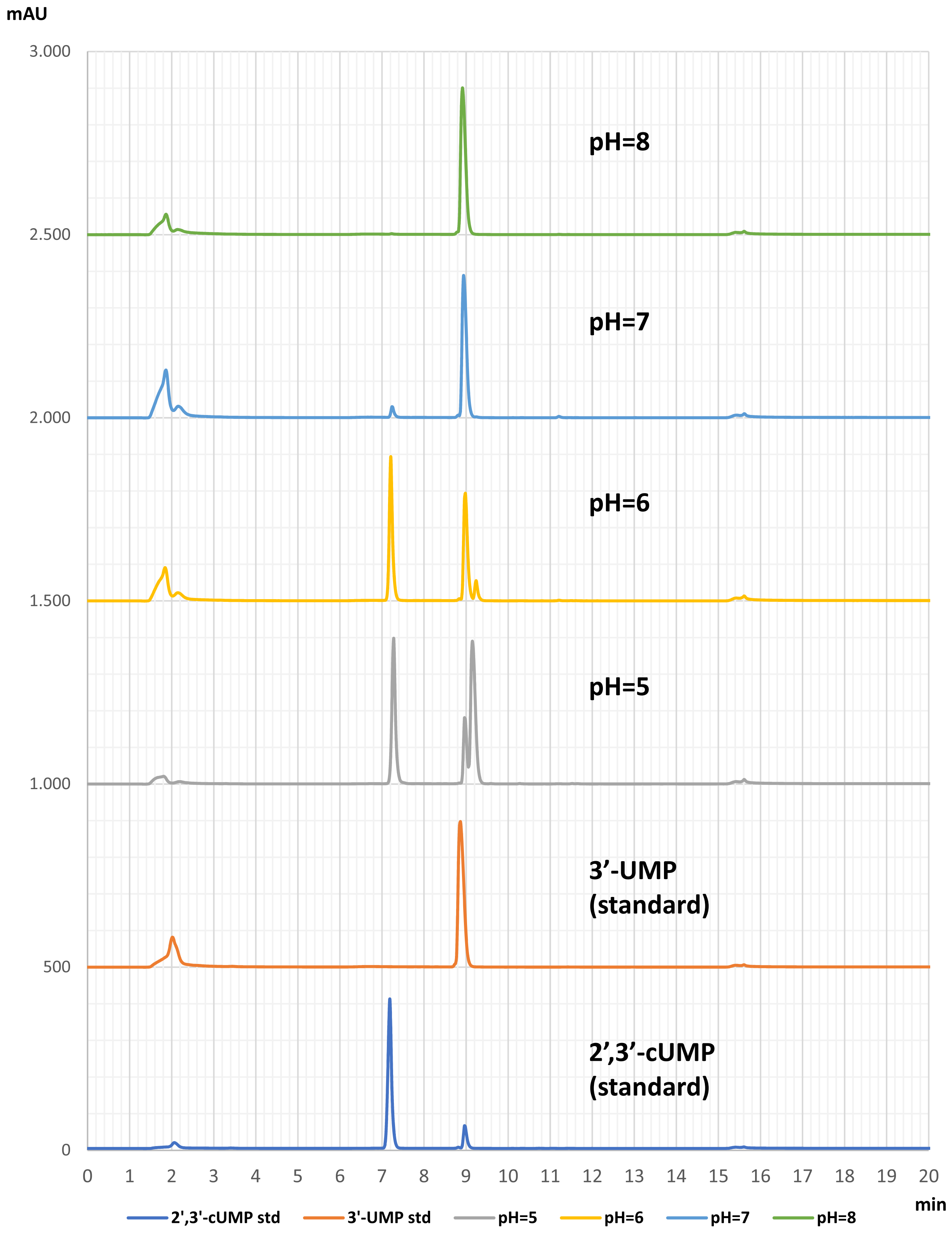

Figure S34. Ion exchange chromatograms of the effect of the pH of a reaction mixture of 3’-UMP (1 µmol, 1eq), DAP (5eq), $MgCl_2$ (5eq) and **2-methylimidazole** (5eq) in 1mL of water at -20°C (*week 4*).

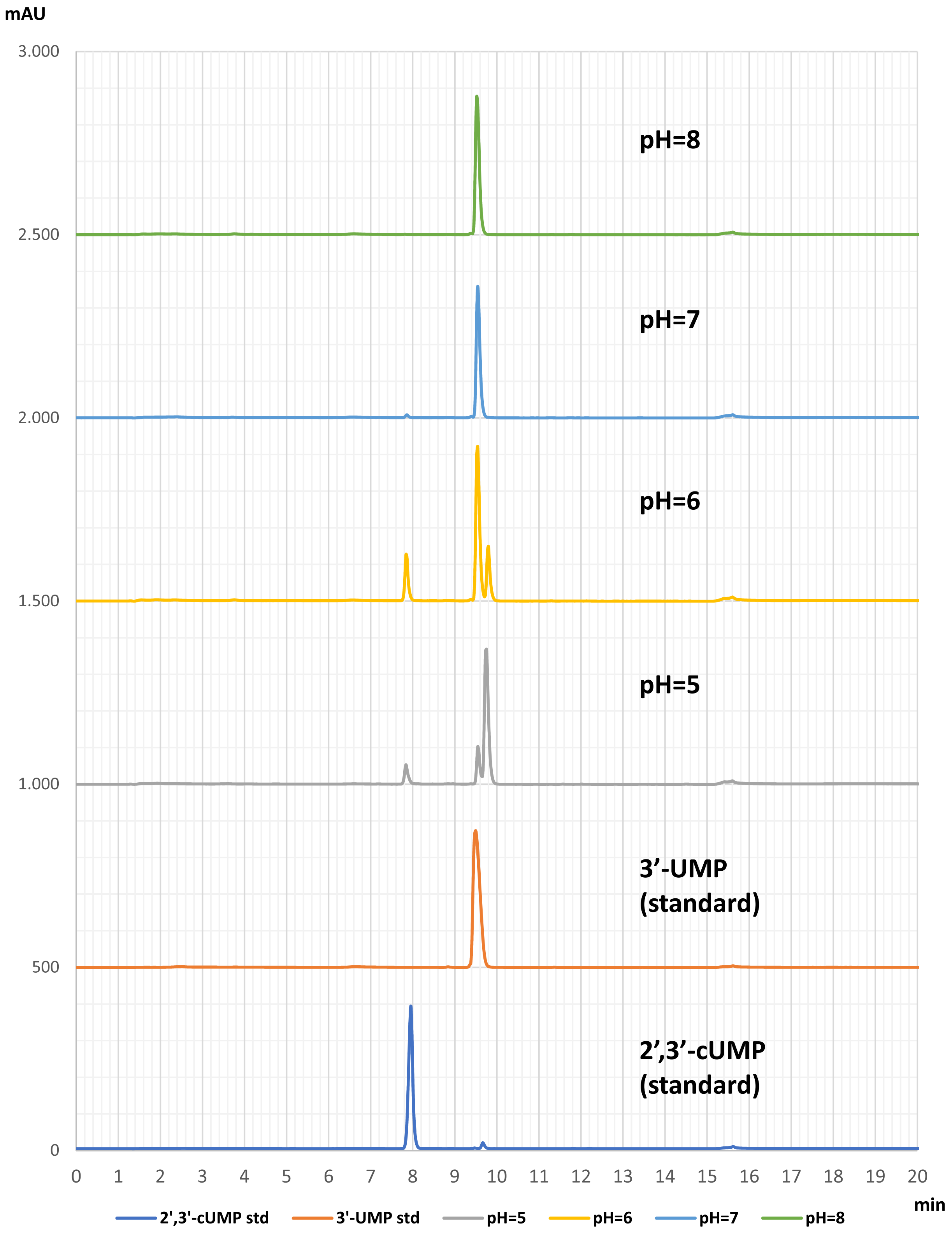

Figure S35. Ion exchange chromatograms of the effect of the pH of a reaction mixture of 3’-UMP (1 µmol, 1eq), DAP (5eq), $MgCl_2$ (5eq) and **2-aminoimidazole** (5eq) in 1mL of water at -20°C (*week 1*).

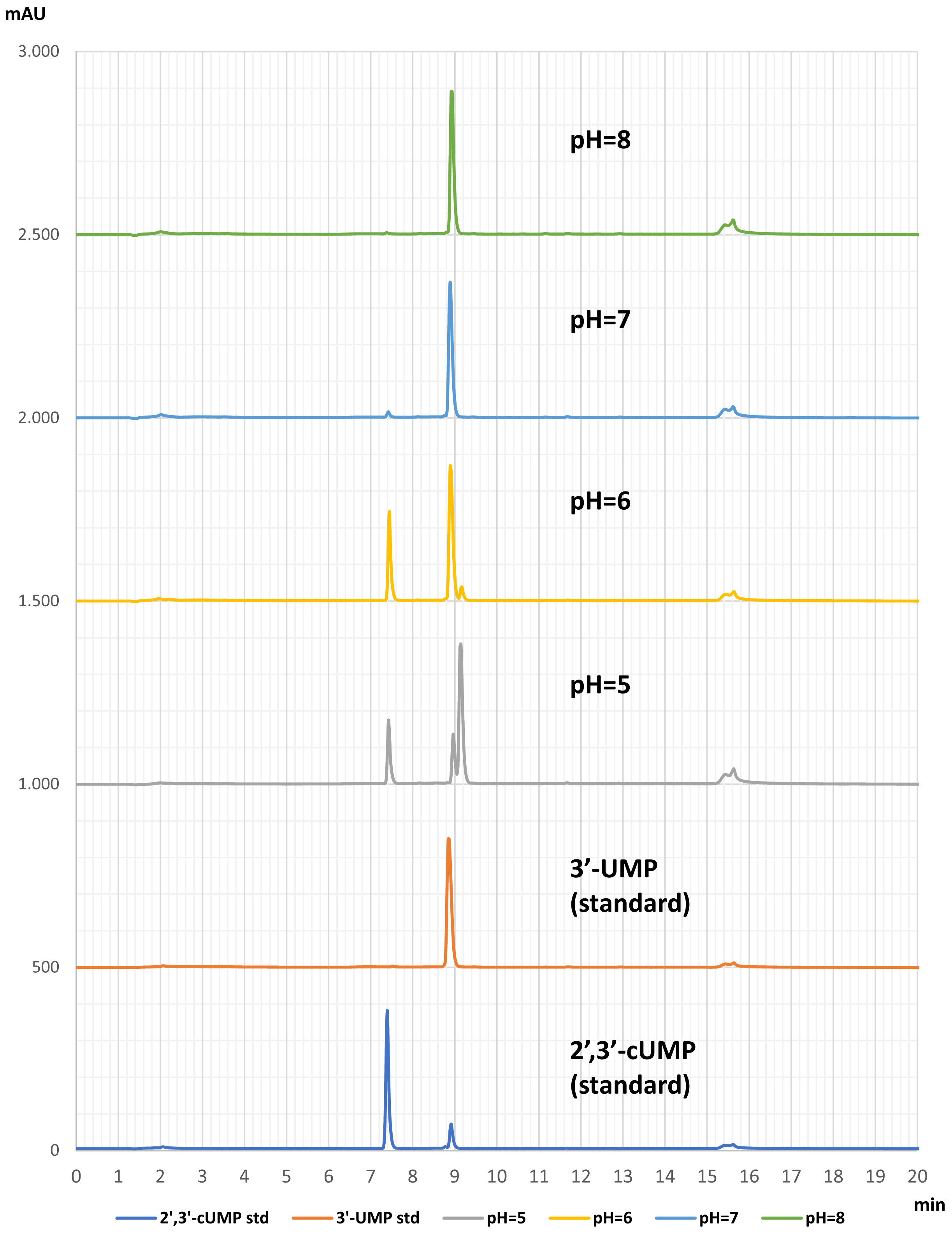

Figure S36. Ion exchange chromatograms of the effect of the pH of a reaction mixture of 3’-UMP (1 µmol, 1eq), DAP (5eq), $MgCl_2$ (5eq) and **2-aminoimidazole** (5eq) in 1mL of water at -20°C (*week 2*).

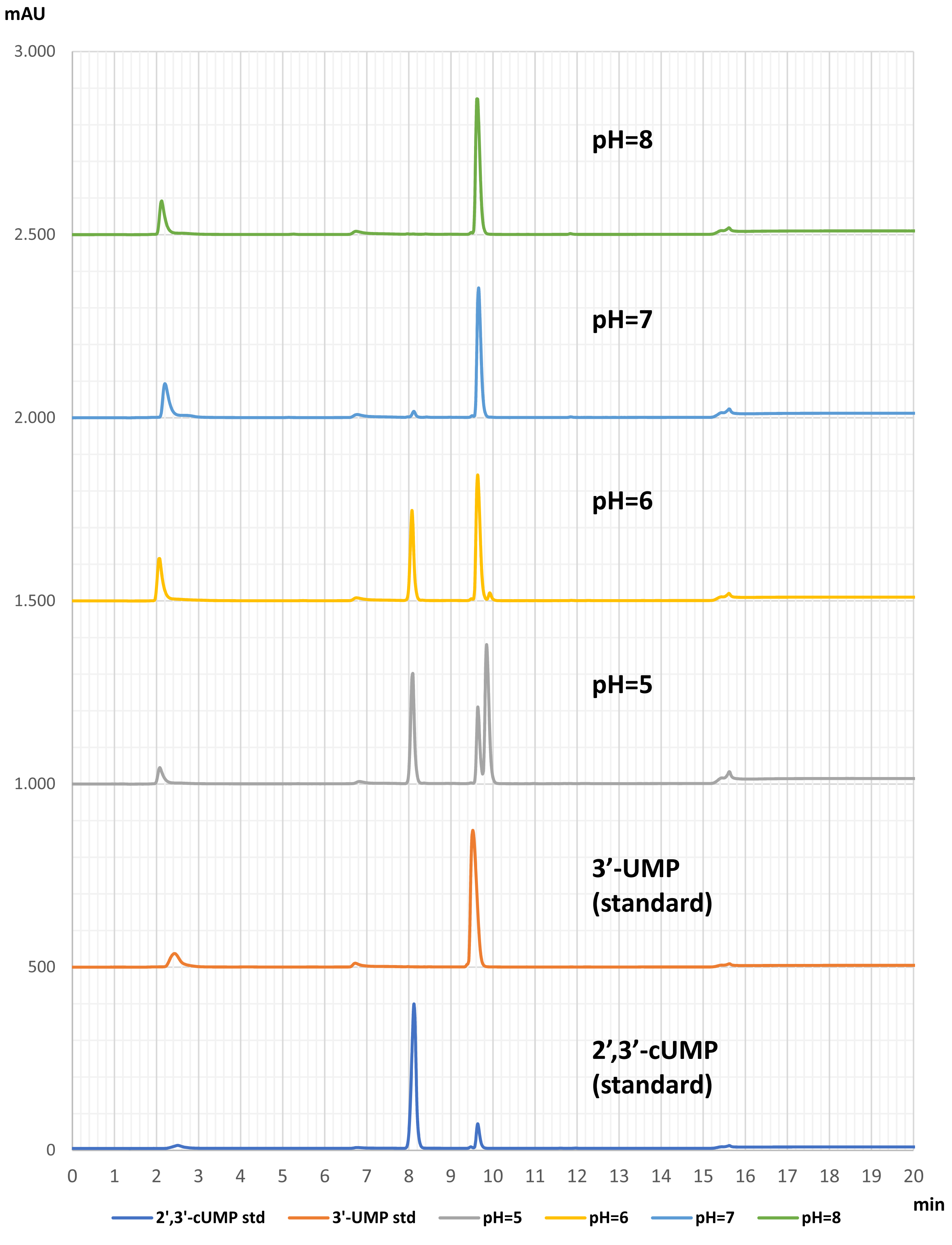

Figure S37. Ion exchange chromatograms of the effect of the pH of a reaction mixture of 3’-UMP (1 µmol, 1eq), DAP (5eq), $MgCl_2$ (5eq) and **2-aminoimidazole** (5eq) in 1mL of water at -20°C (*week 3*).

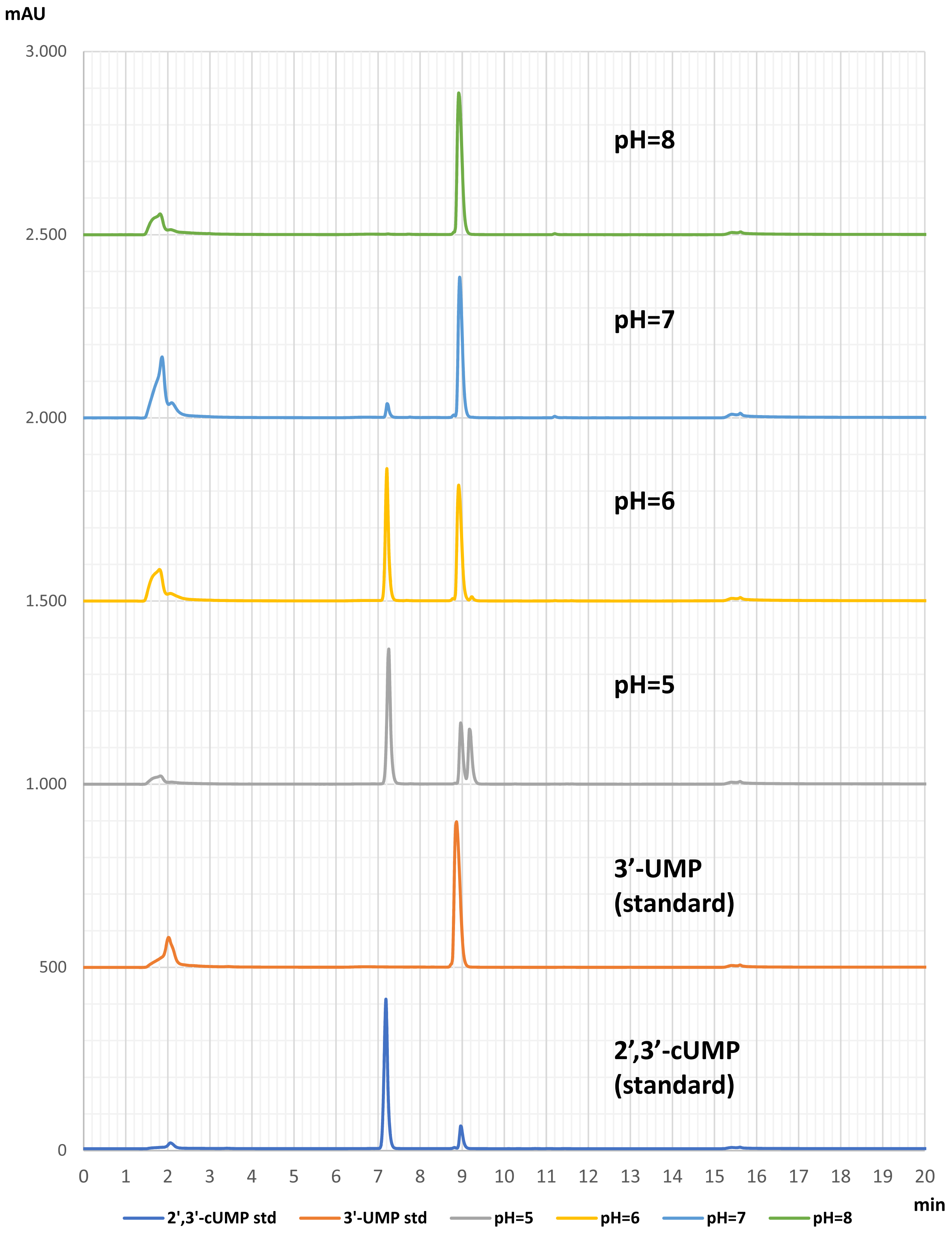

Figure S38. Ion exchange chromatograms of the effect of the pH of a reaction mixture of 3’-UMP (1 µmol, 1eq), DAP (5eq), $MgCl_2$ (5eq) and **2-aminoimidazole** (5eq) in 1mL of water at -20°C (*week 4*).

7.2 Chromatograms 3’-CMP → 2’,3’-cCMP with different activators (weeks 1 to 4)

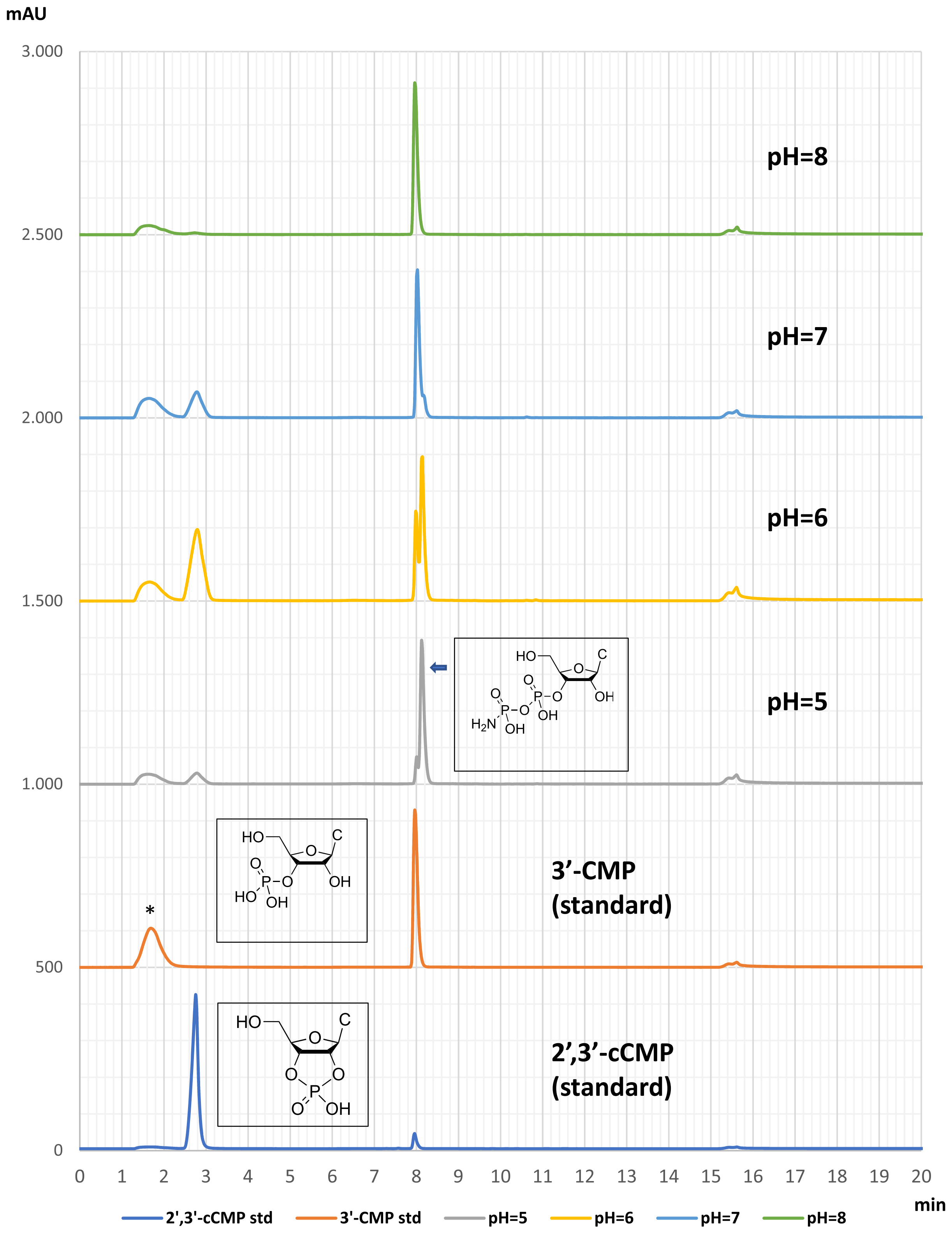

Figure S39. Ion exchange chromatograms of the effect of the pH of a reaction mixture of 3’-CMP (1 µmol, 1eq), DAP (5eq), $MgCl_2$ (5eq) and **imidazole** (5eq) in 1mL of water at -20°C (*week 1*). * unknown peak present in SM.

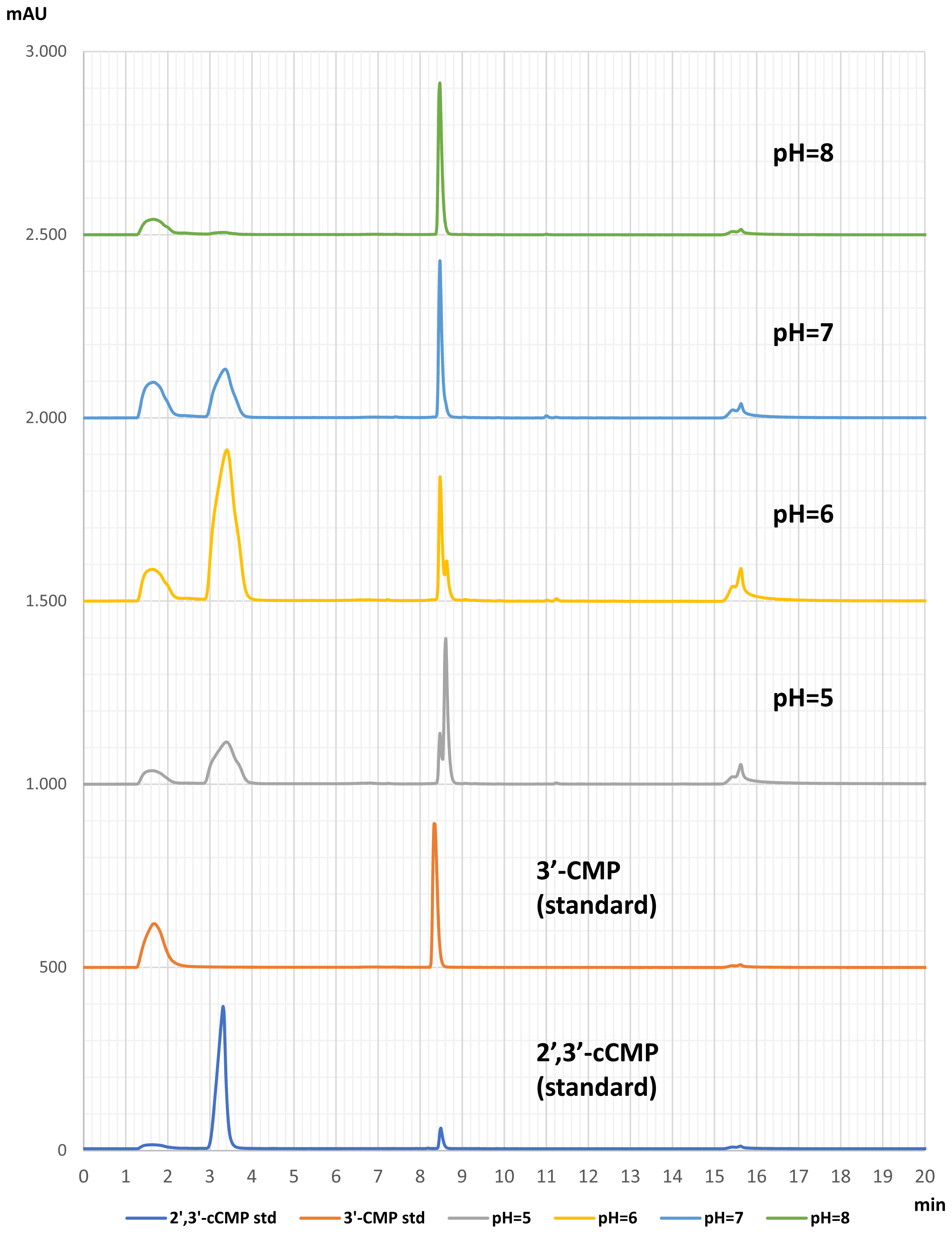

Figure S40. Ion exchange chromatograms of the effect of the pH of a reaction mixture of 3’-CMP (1 µmol, 1eq), DAP (5eq), $MgCl_2$ (5eq) and **imidazole** (5eq) in 1mL of water at -20°C (*week 2*).

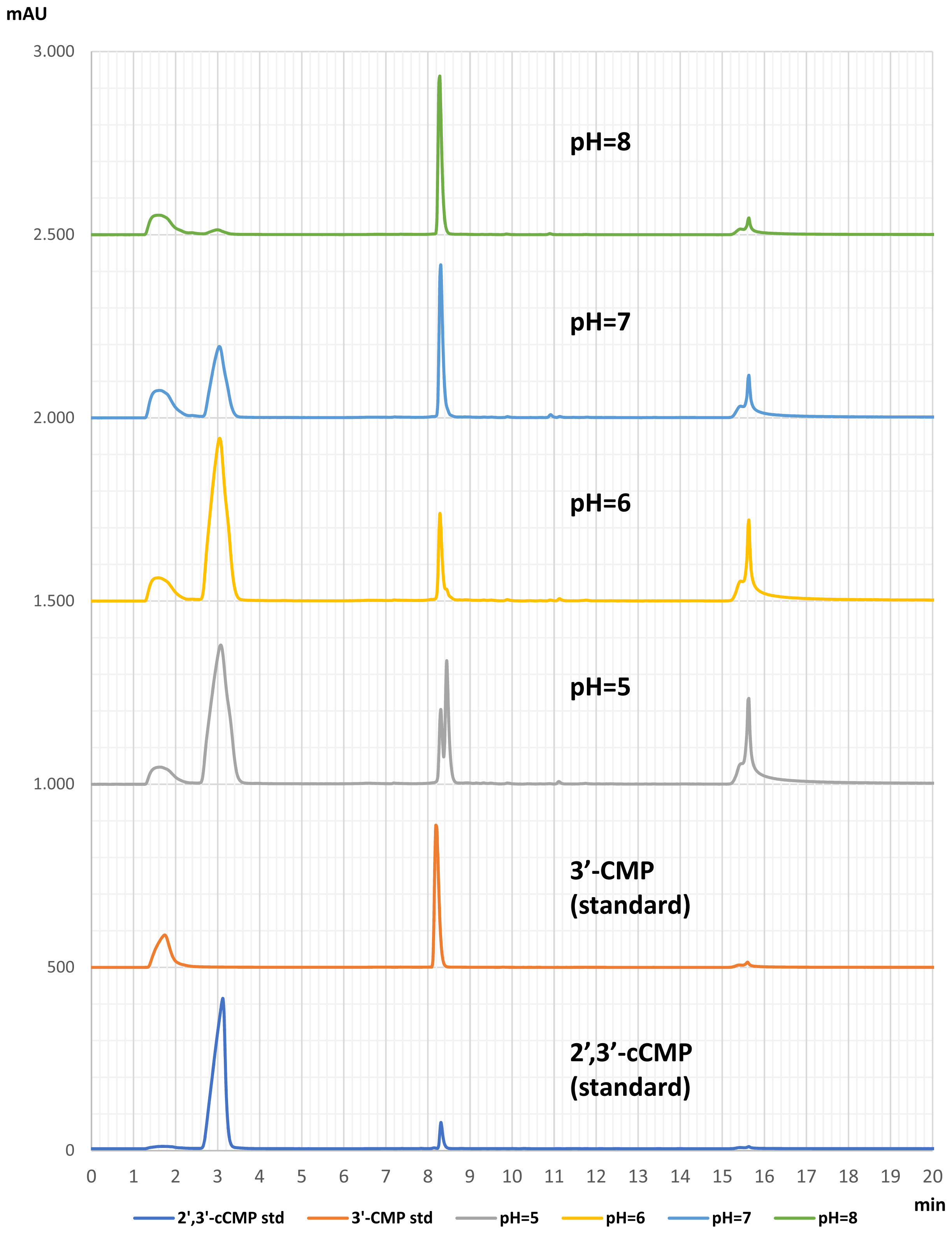

Figure S41. Ion exchange chromatograms of the effect of the pH of a reaction mixture of 3’-CMP (1 µmol, 1eq), DAP (5eq), $MgCl_2$ (5eq) and **imidazole** (5eq) in 1mL of water at -20°C (*week 3*).

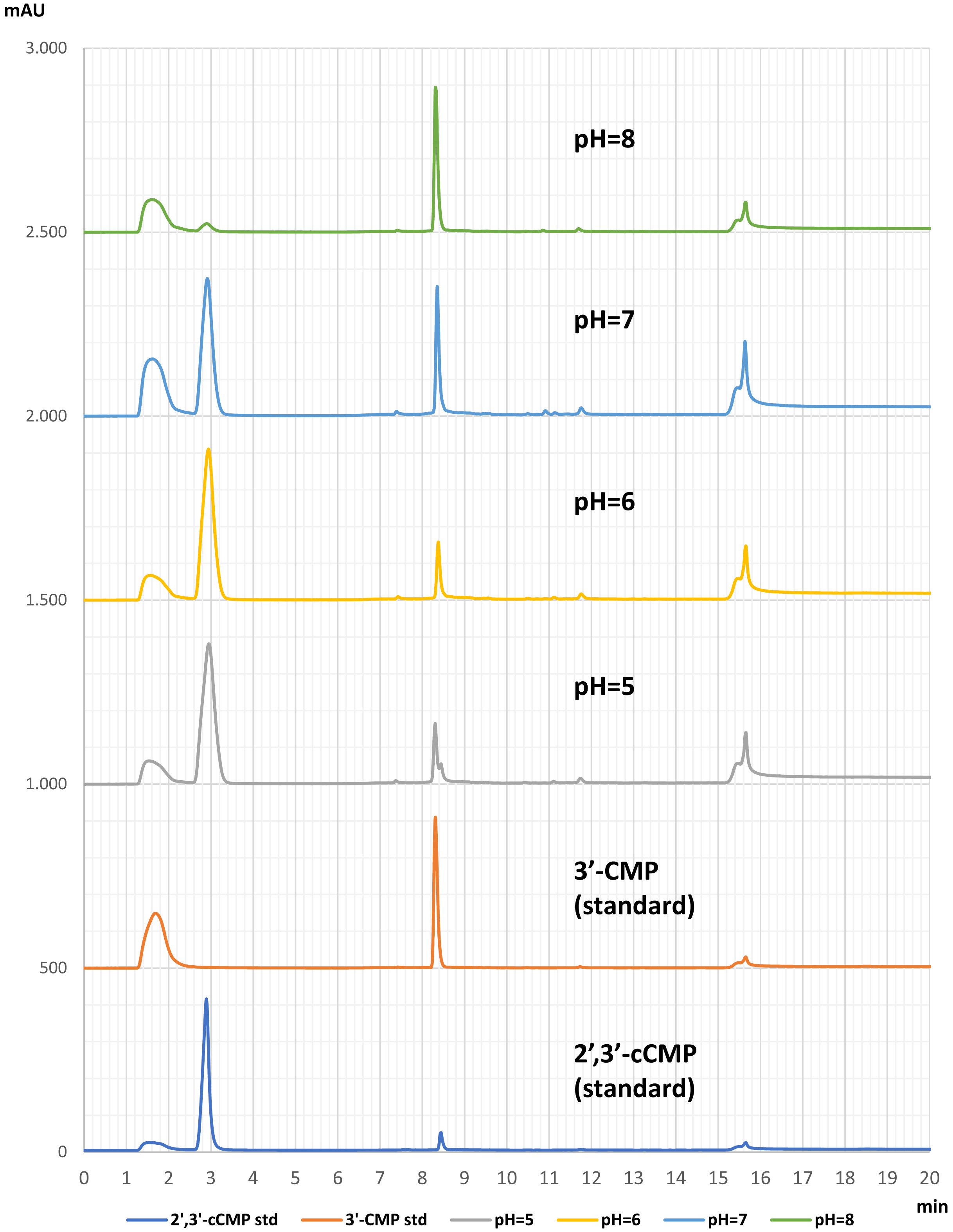

Figure S42. Ion exchange chromatograms of the effect of the pH of a reaction mixture of 3’-CMP (1 µmol, 1eq), DAP (5eq), $MgCl_2$ (5eq) and **imidazole** (5eq) in 1mL of water at -20°C (*week 4*).

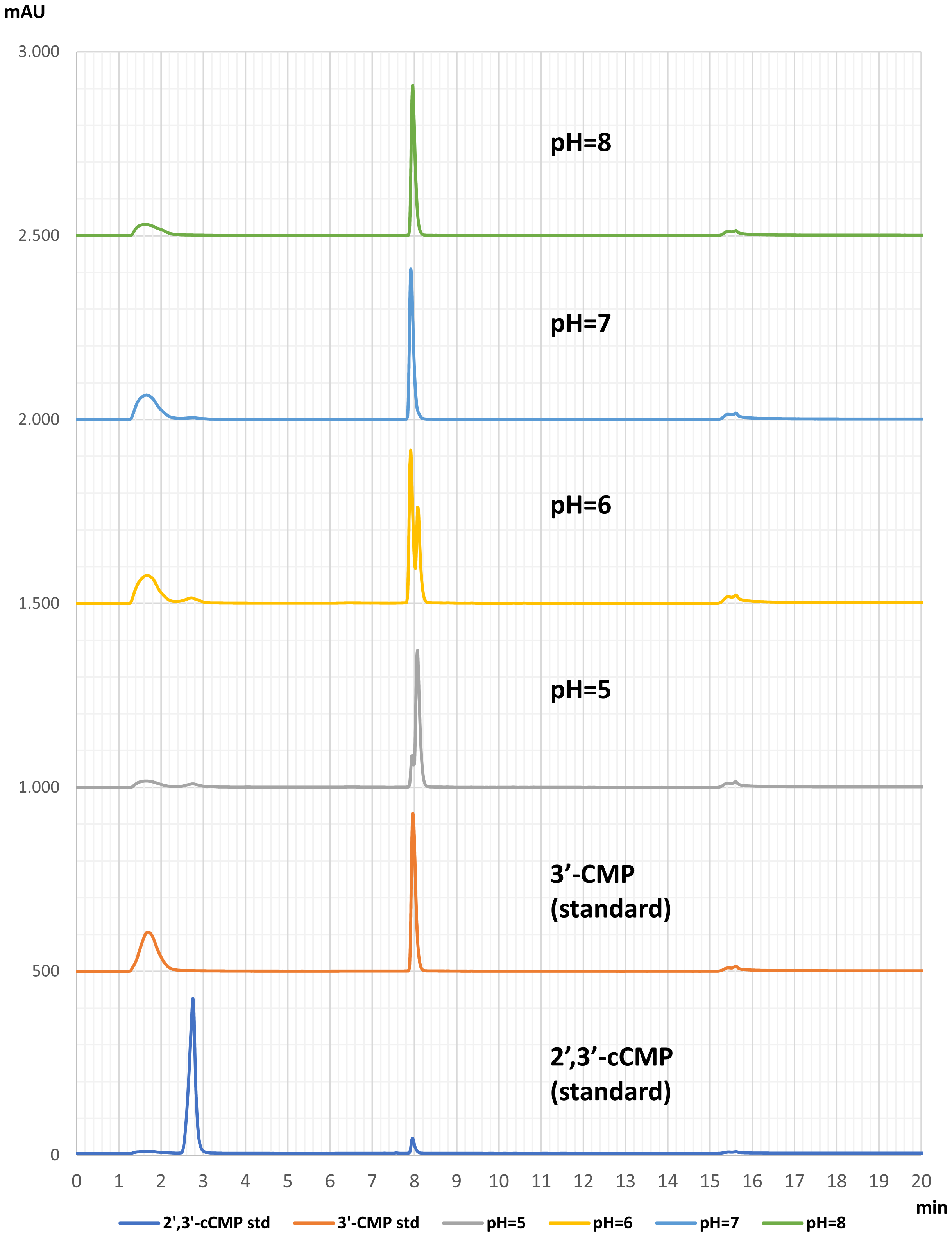

Figure S43. Ion exchange chromatograms of the effect of the pH of a reaction mixture of 3’-CMP (1 µmol, 1eq), DAP (5eq), $MgCl_2$ (5eq) and **2-methylimidazole** (5eq) in 1mL of water at -20°C (*week 1*).

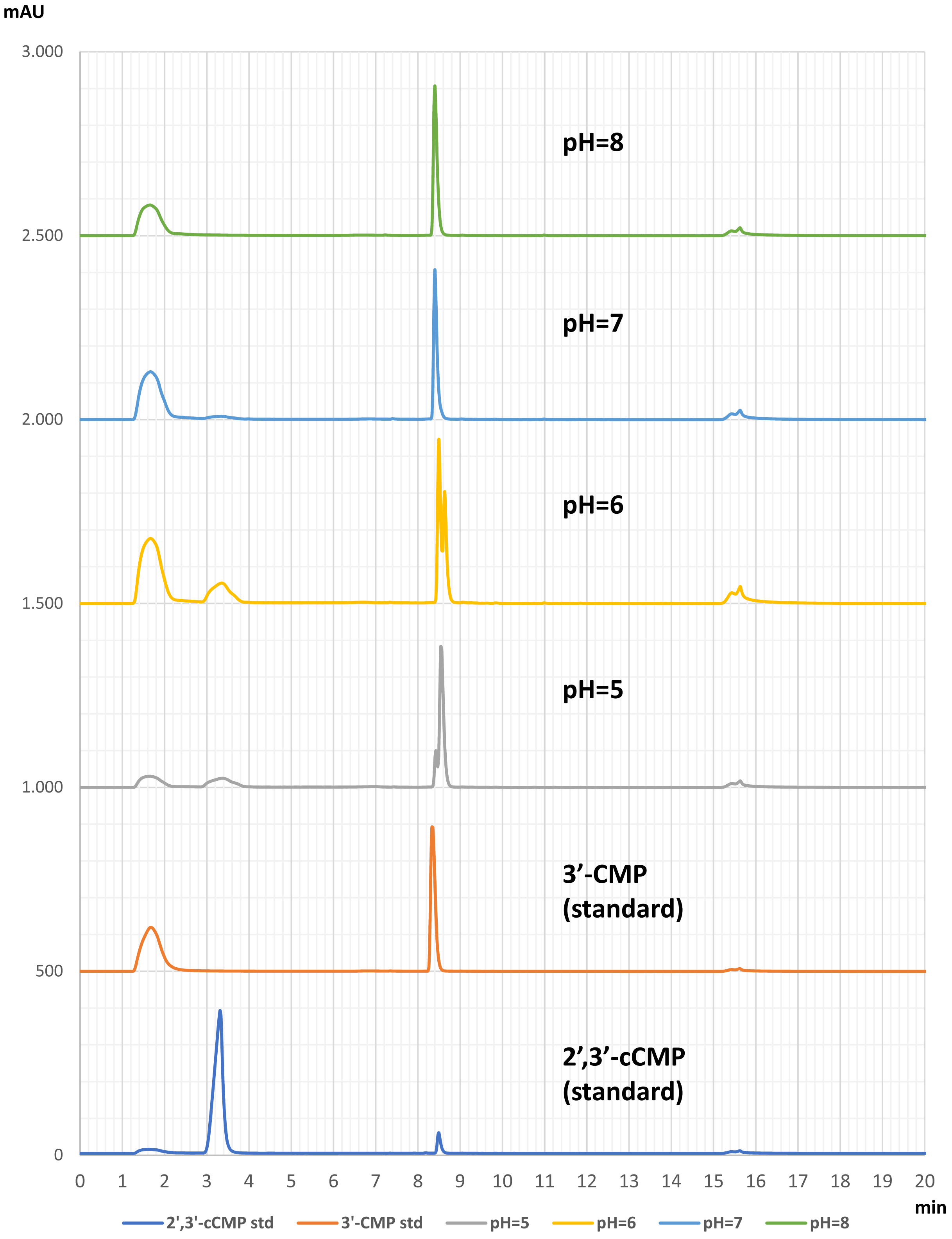

Figure S44. Ion exchange chromatograms of the effect of the pH of a reaction mixture of 3’-CMP (1 µmol, 1eq), DAP (5eq), $MgCl_2$ (5eq) and **2-methylimidazole** (5eq) in 1mL of water at -20°C (*week 2*).

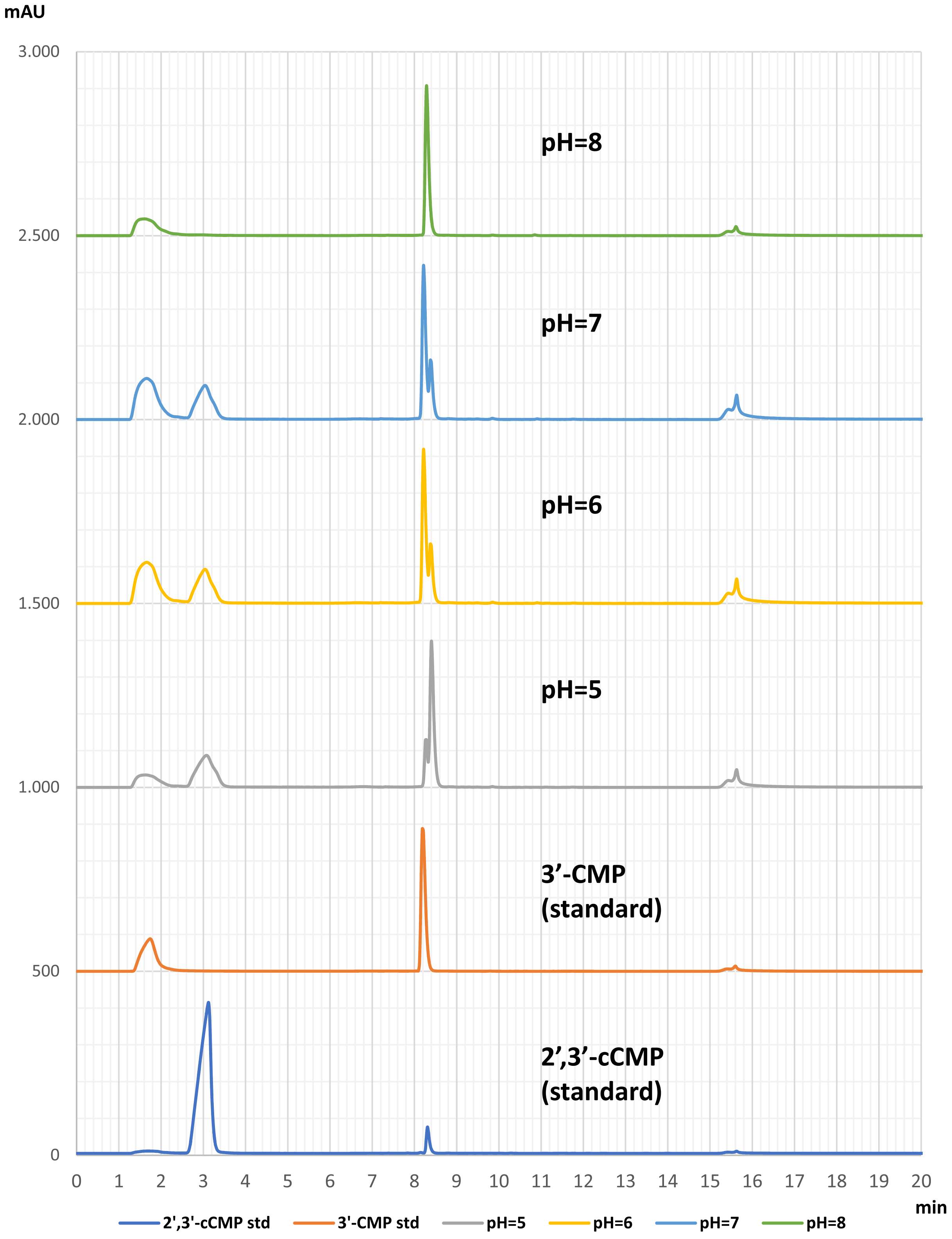

Figure S45. Ion exchange chromatograms of the effect of the pH of a reaction mixture of 3’-CMP (1 µmol, 1eq), DAP (5eq), $MgCl_2$ (5eq) and **2-methylimidazole** (5eq) in 1mL of water at -20°C (*week 3*).

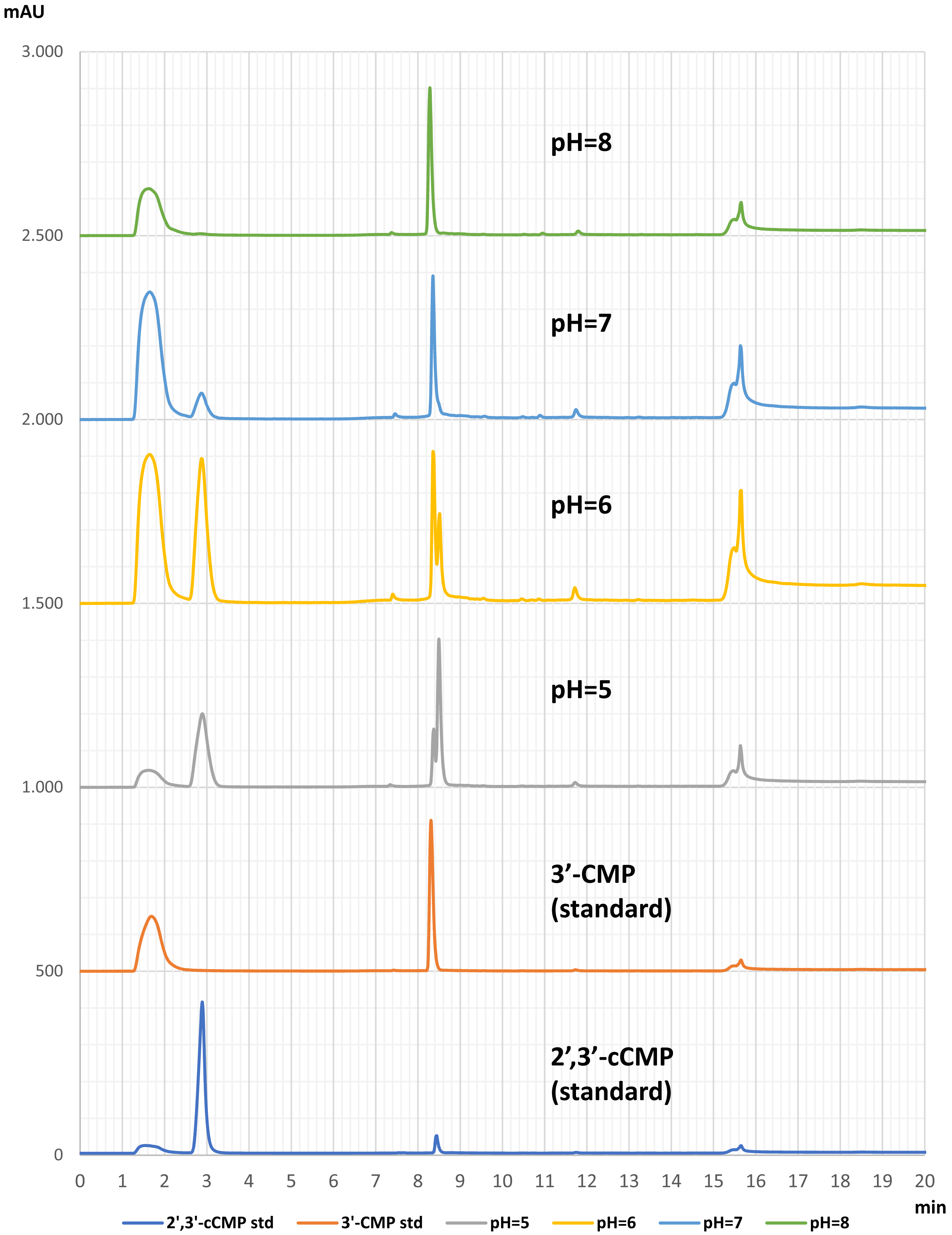

Figure S46. Ion exchange chromatograms of the effect of the pH of a reaction mixture of 3’-CMP (1 µmol, 1eq), DAP (5eq), $MgCl_2$ (5eq) and **2-methylimidazole** (5eq) in 1mL of water at -20°C (*week 4*).

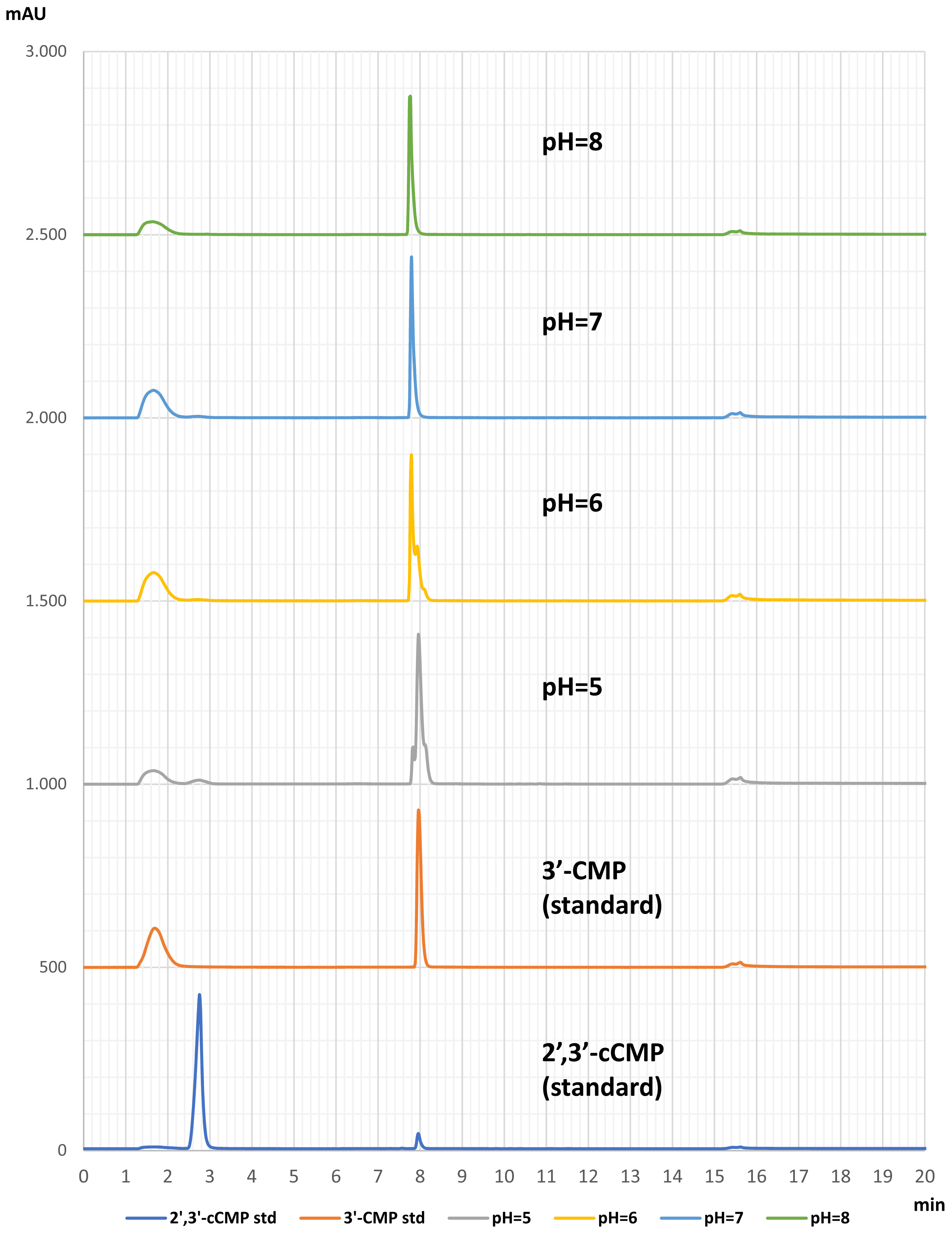

Figure S47. Ion exchange chromatograms of the effect of the pH of a reaction mixture of 3’-CMP (1 µmol, 1eq), DAP (5eq), $MgCl_2$ (5eq) and **2-aminoimidazole** (5eq) in 1mL of water at -20°C (*week 1*).

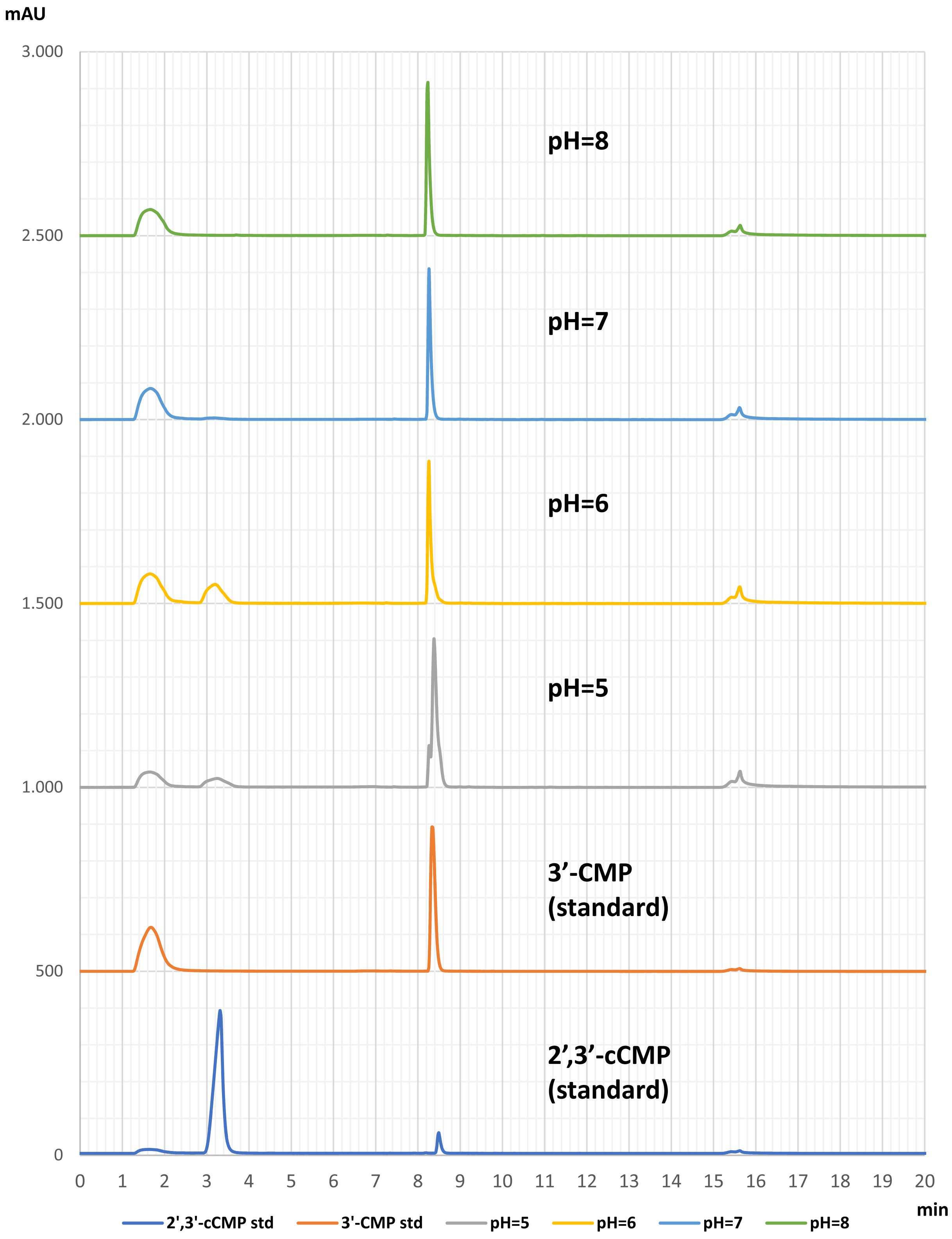

Figure S48. Ion exchange chromatograms of the effect of the pH of a reaction mixture of 3'-CMP (1 µmol, 1eq), DAP (5eq), $MgCl_2$ (5eq) and **2-aminoimidazole** (5eq) in 1mL of water at -20°C (*week 2*).

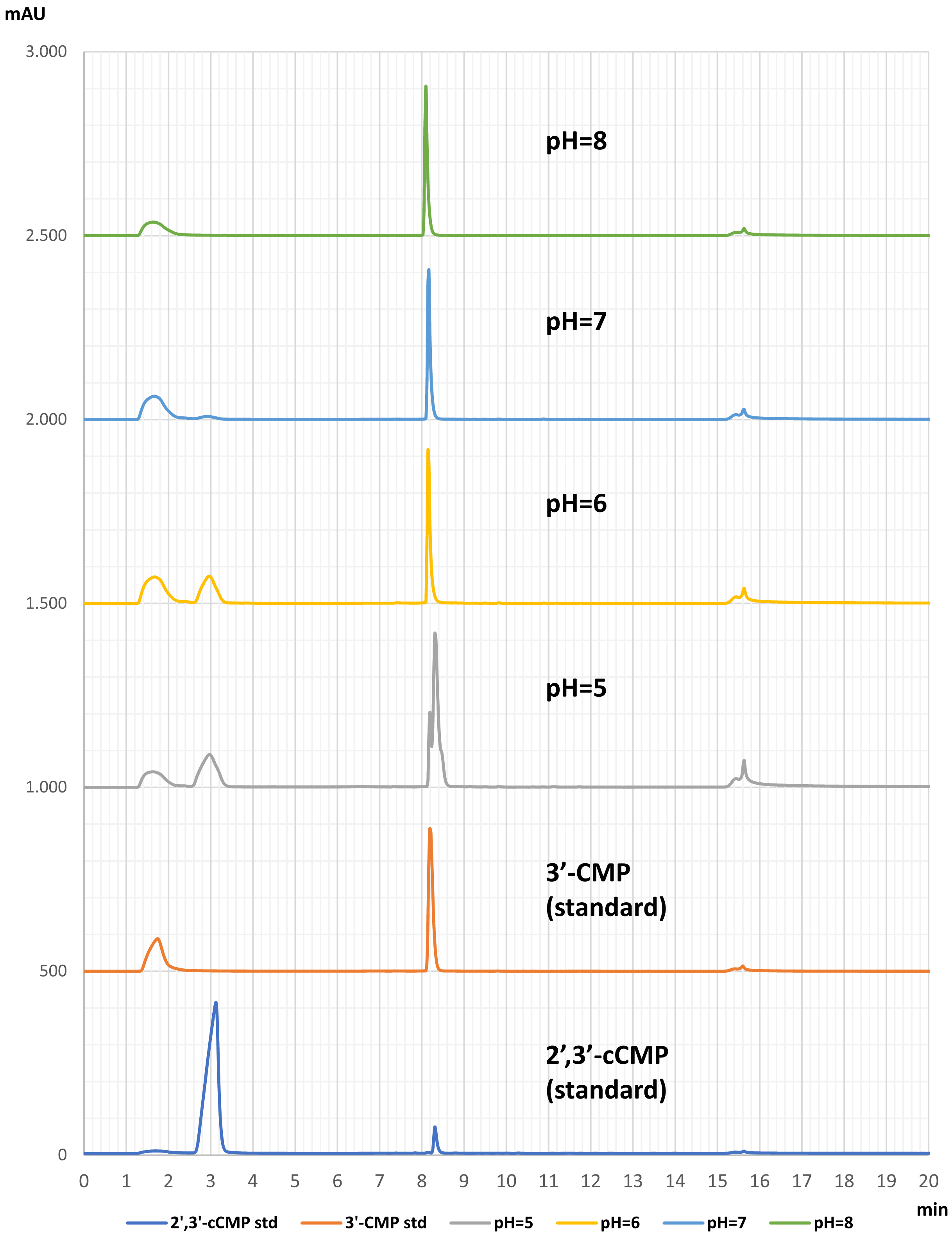

Figure S49. Ion exchange chromatograms of the effect of the pH of a reaction mixture of 3’-CMP (1 µmol, 1eq), DAP (5eq), $MgCl_2$ (5eq) and **2-aminoimidazole** (5eq) in 1mL of water at -20°C (*week 3*).

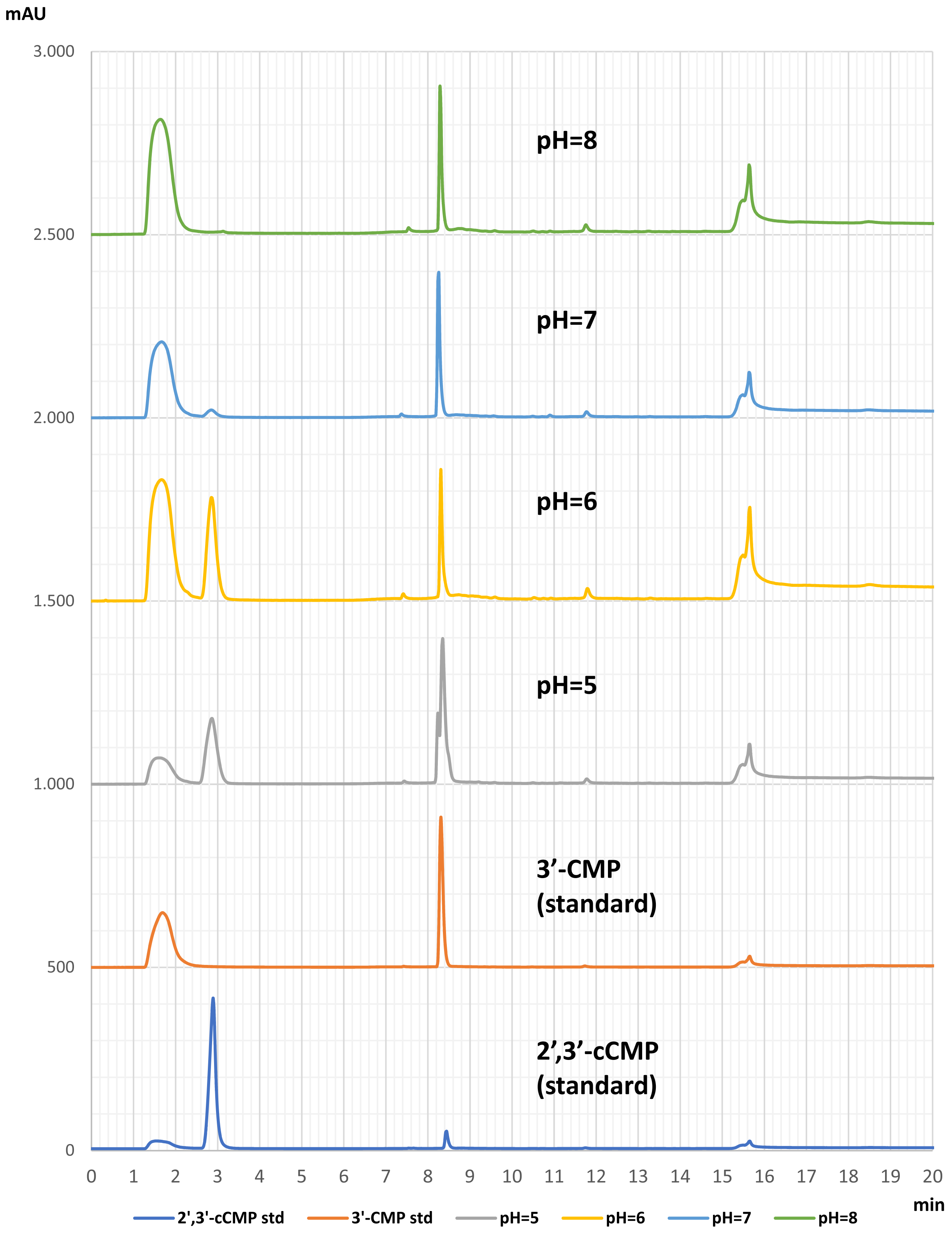

Figure S50. Ion exchange chromatograms of the effect of the pH of a reaction mixture of 3'-CMP (1 µmol, 1eq), DAP (5eq), $MgCl_2$ (5eq) and **2-aminoimidazole** (5eq) in 1mL of water at -20°C (*week 4*).

7.3 Chromatograms 3'-AMP → 2',3'-cAMP with different activators (weeks 1 to 4)

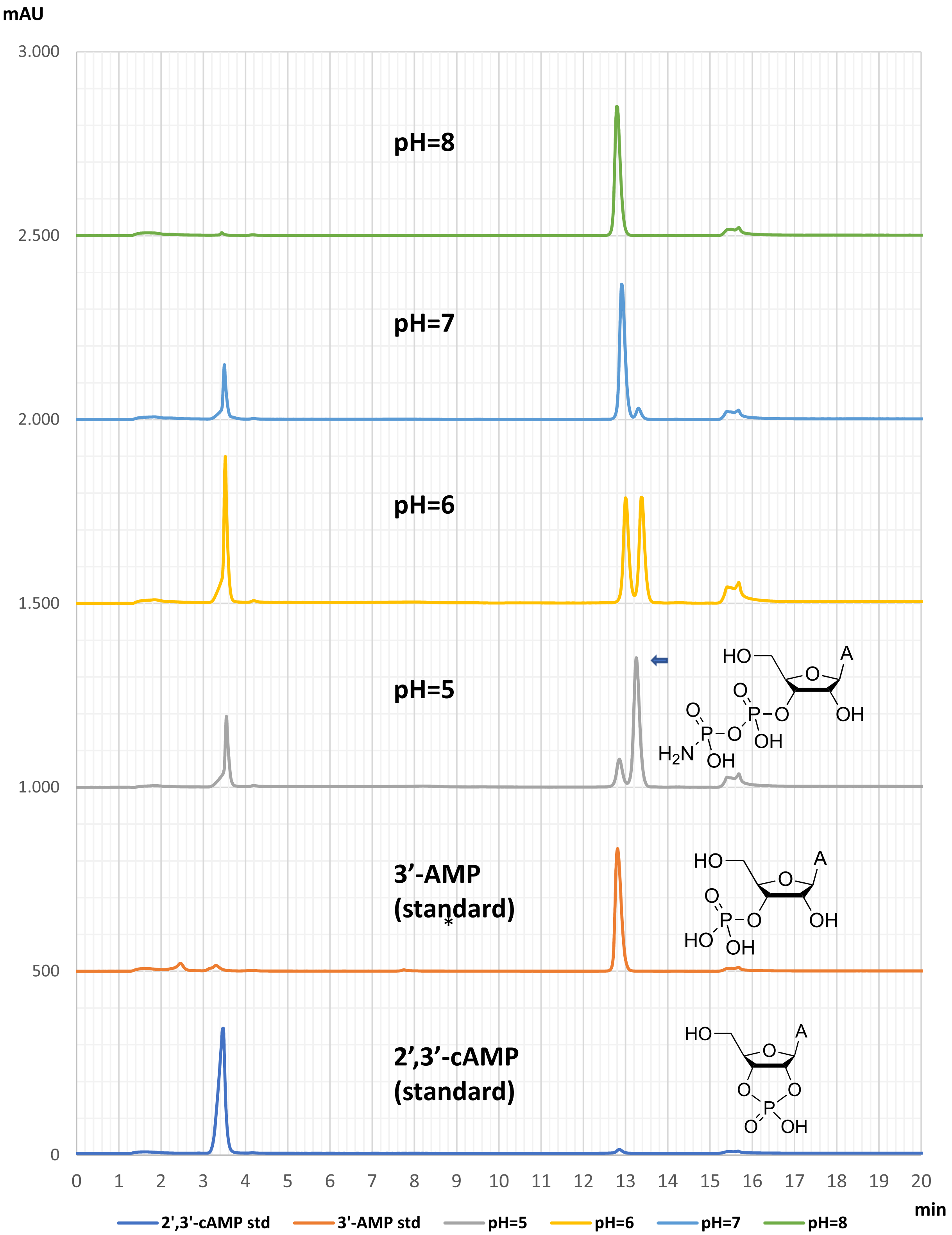

Figure S51. Ion exchange chromatograms of the effect of the pH of a reaction mixture of 3’-AMP (1 µmol, 1eq), DAP (5eq), $MgCl_2$ (5eq) and **imidazole** (5eq) in 1mL of water at -20°C (*week 1*).

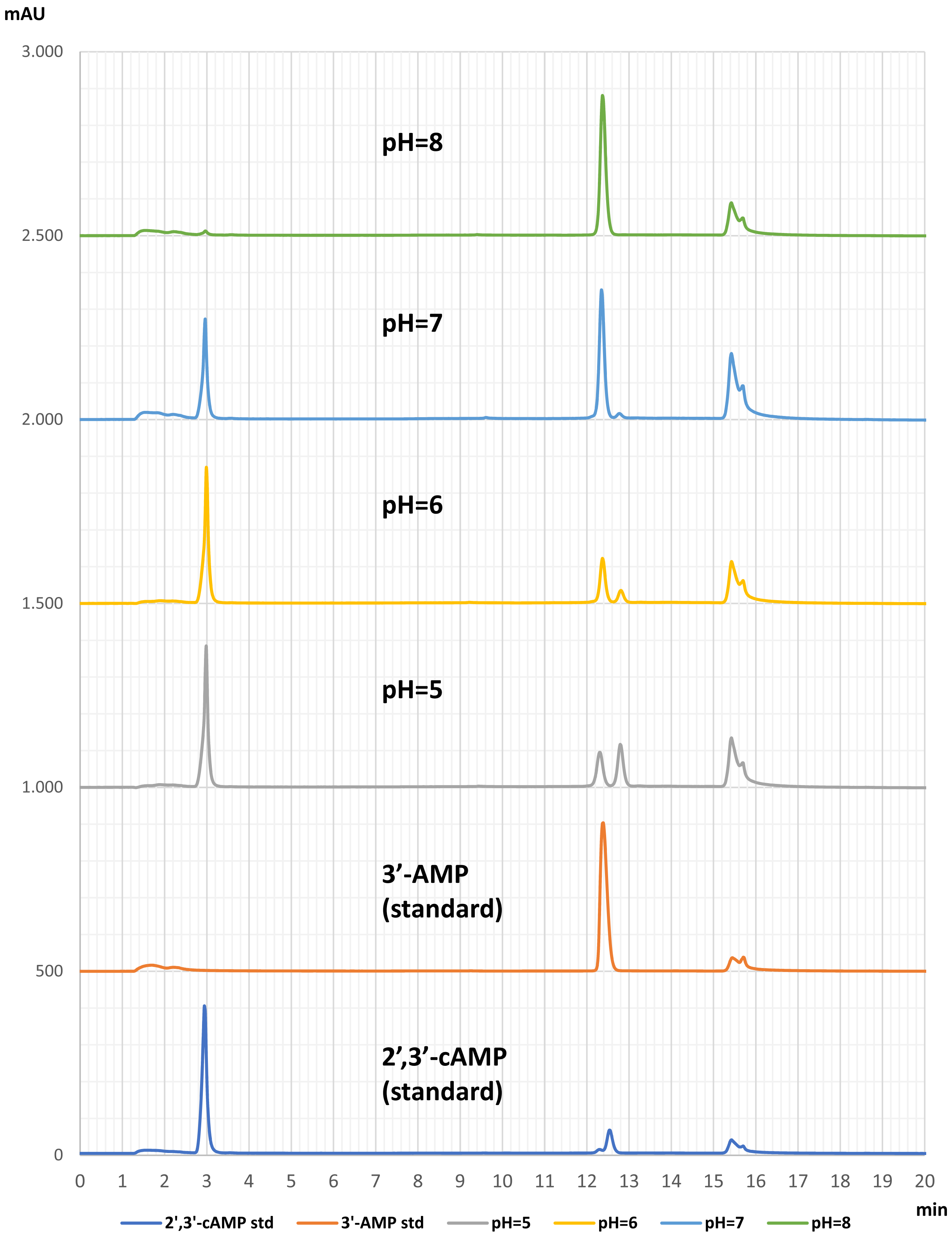

Figure S52. Ion exchange chromatograms of the effect of the pH of a reaction mixture of 3’-AMP (1 µmol, 1eq), DAP (5eq), $MgCl_2$ (5eq) and **imidazole** (5eq) in 1mL of water at -20°C (*week 2*).

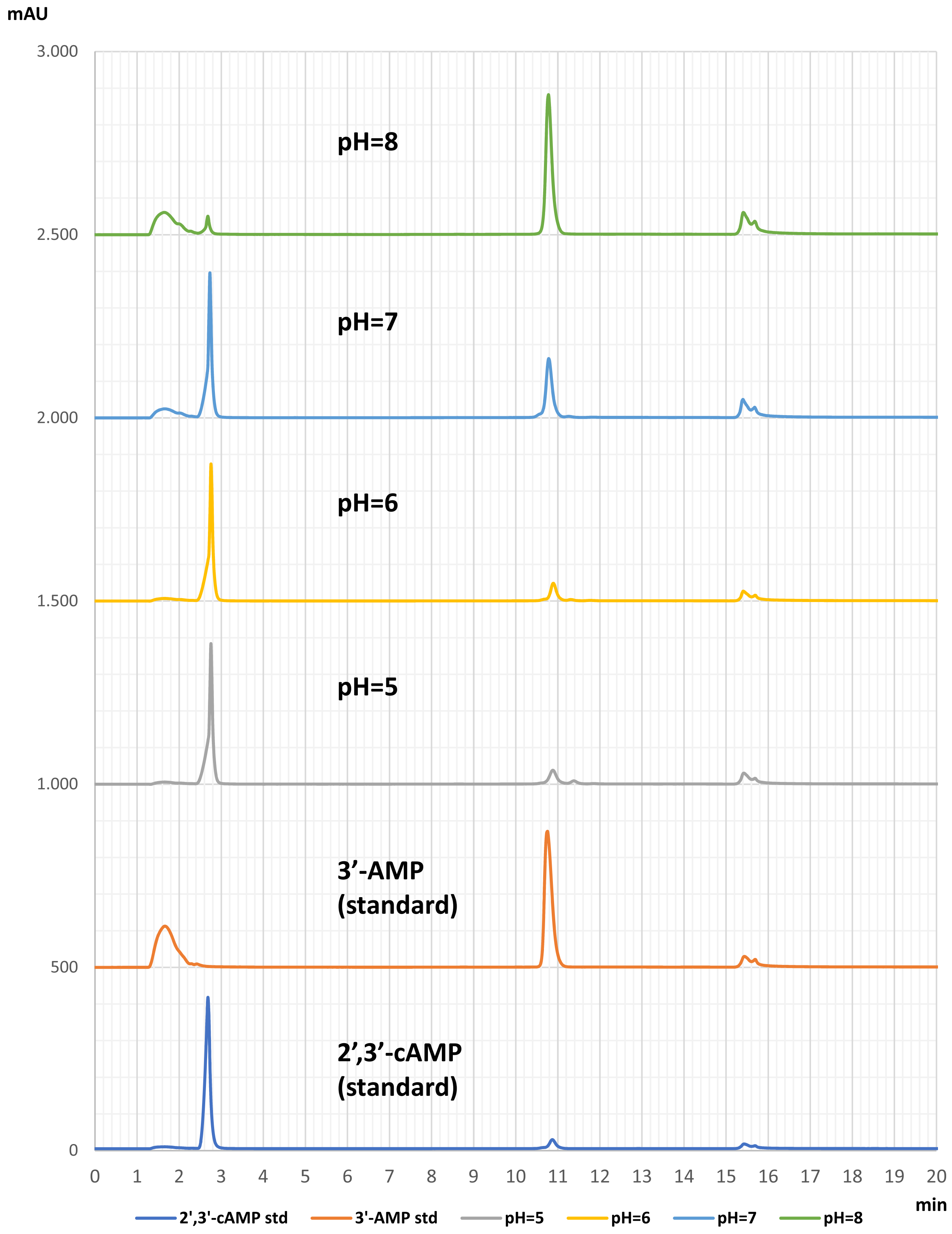

Figure S53. Ion exchange chromatograms of the effect of the pH of a reaction mixture of 3’-AMP (1 µmol, 1eq), DAP (5eq), $MgCl_2$ (5eq) and **imidazole** (5eq) in 1mL of water at -20°C (*week 3*).

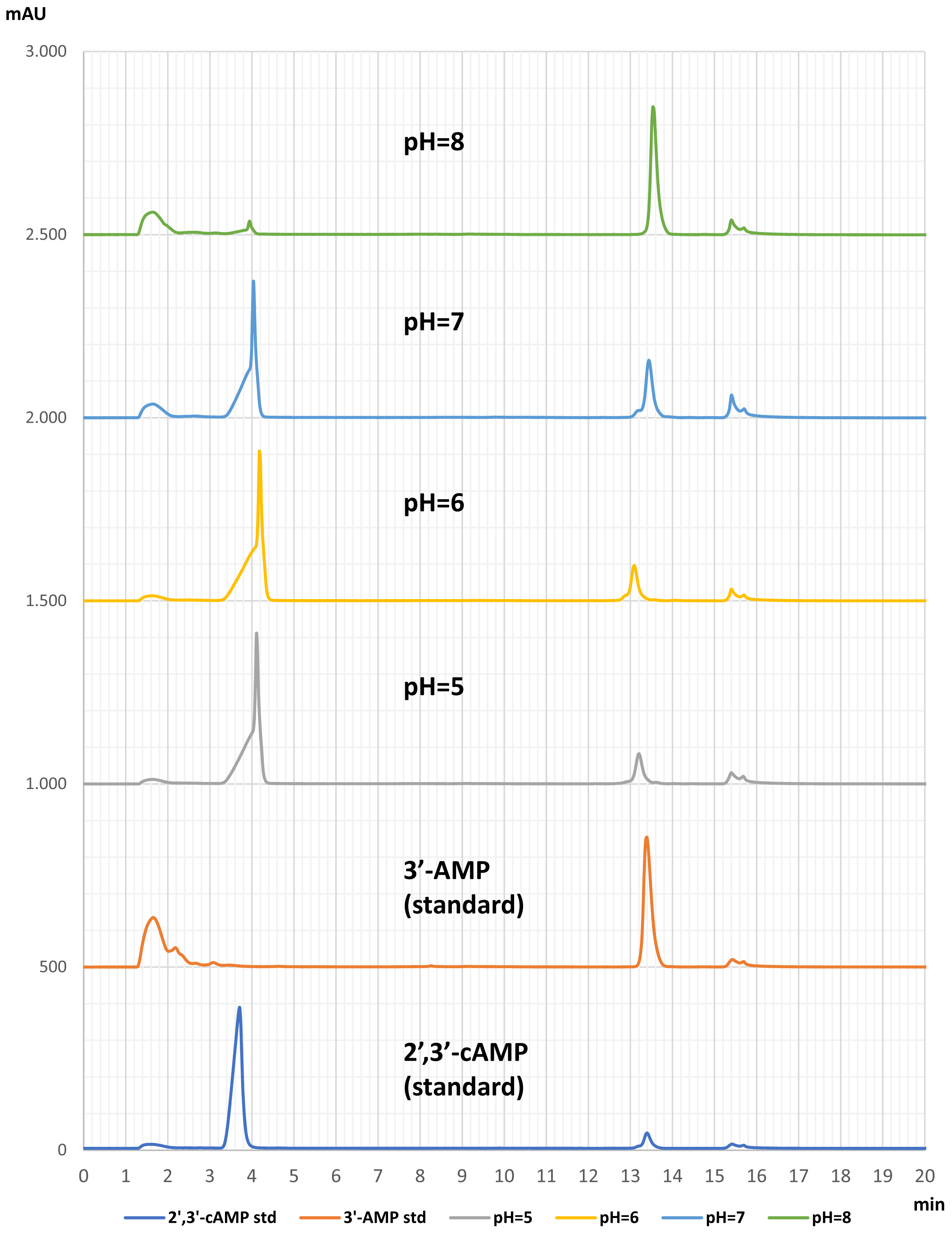

Figure S54. Ion exchange chromatograms of the effect of the pH of a reaction mixture of 3’-AMP (1 µmol, 1eq), DAP (5eq), $MgCl_2$ (5eq) and **imidazole** (5eq) in 1mL of water at -20°C (*week 4*).

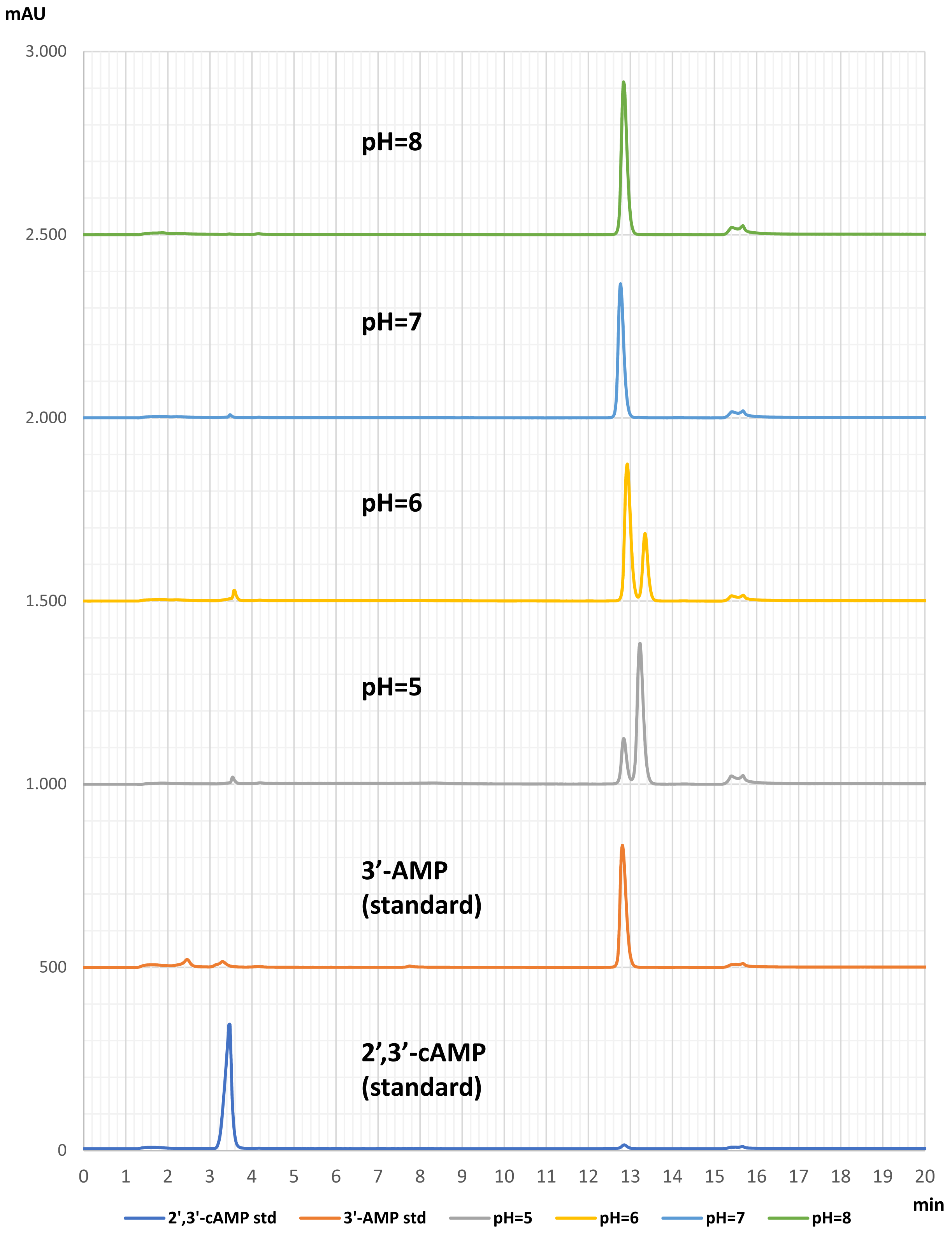

Figure S55. Ion exchange chromatograms of the effect of the pH of a reaction mixture of 3’-AMP (1 µmol, 1eq), DAP (5eq), $MgCl_2$ (5eq) and **2-methylimidazole** (5eq) in 1mL of water at -20°C (*week 1*).

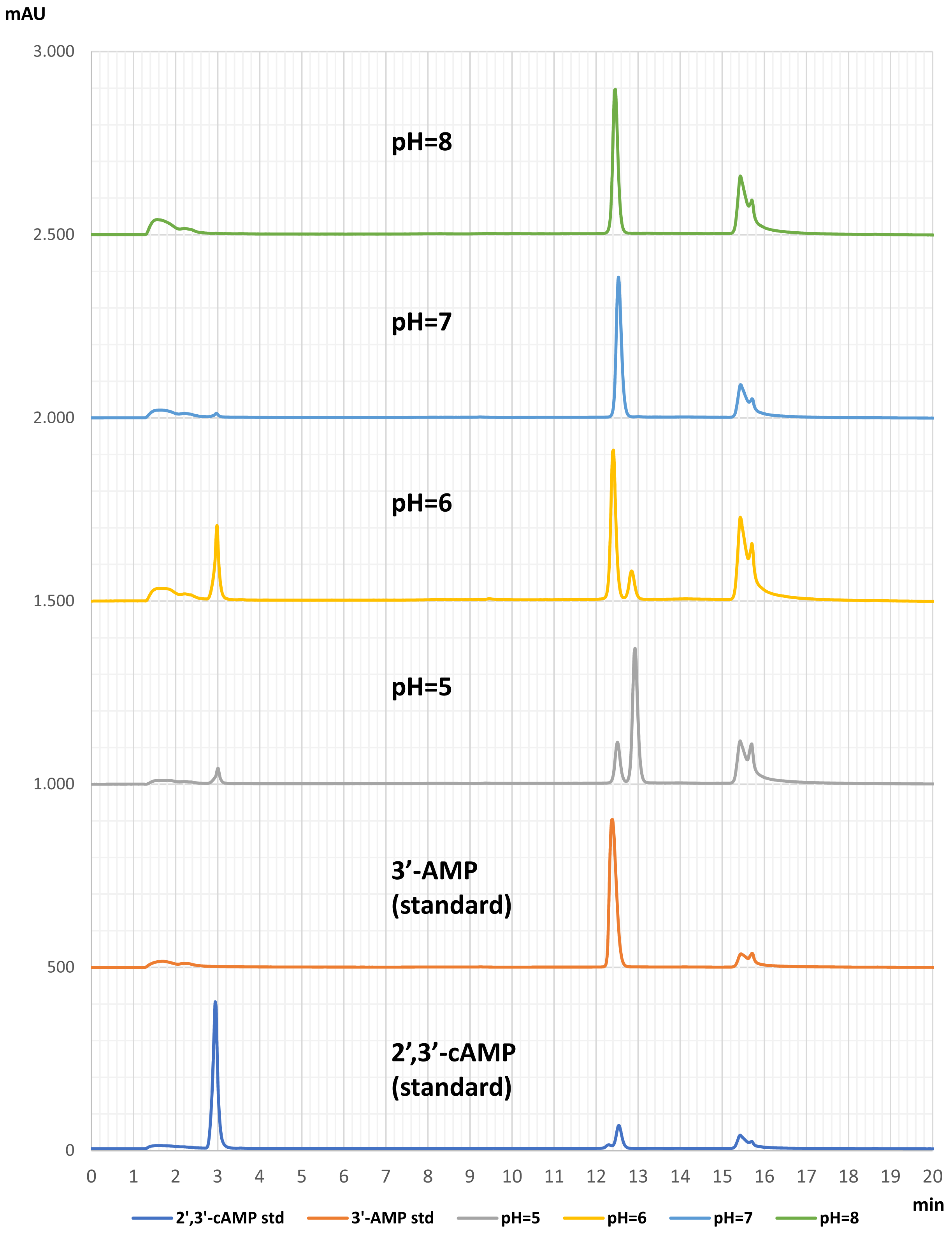

Figure S56. Ion exchange chromatograms of the effect of the pH of a reaction mixture of 3’-AMP (1 µmol, 1eq), DAP (5eq), $MgCl_2$ (5eq) and **2-methylimidazole** (5eq) in 1mL of water at -20°C (*week 2*).

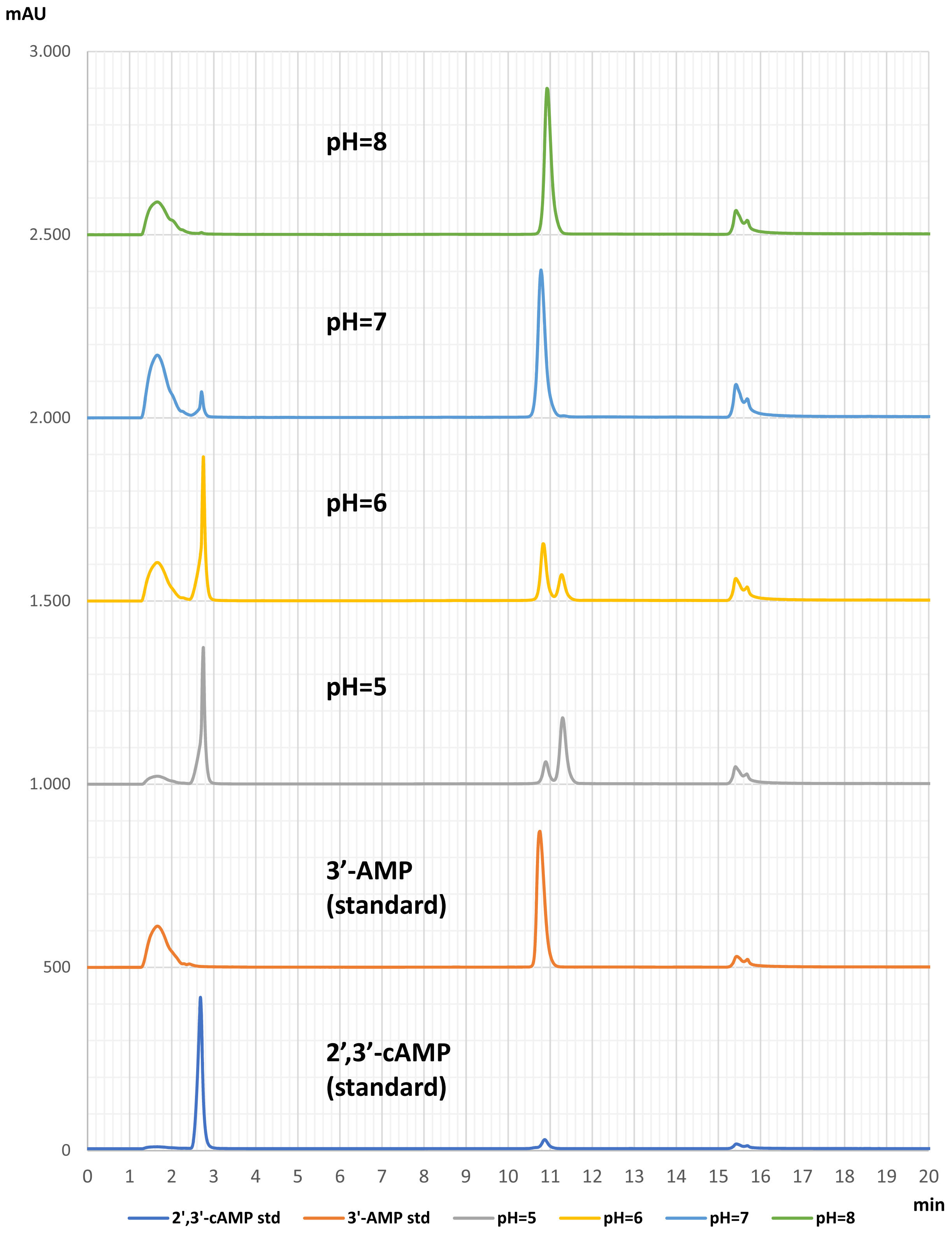

Figure S57. Ion exchange chromatograms of the effect of the pH of a reaction mixture of 3’-AMP (1 µmol, 1eq), DAP (5eq), $MgCl_2$ (5eq) and **2-methylimidazole** (5eq) in 1mL of water at -20°C (*week 3*).

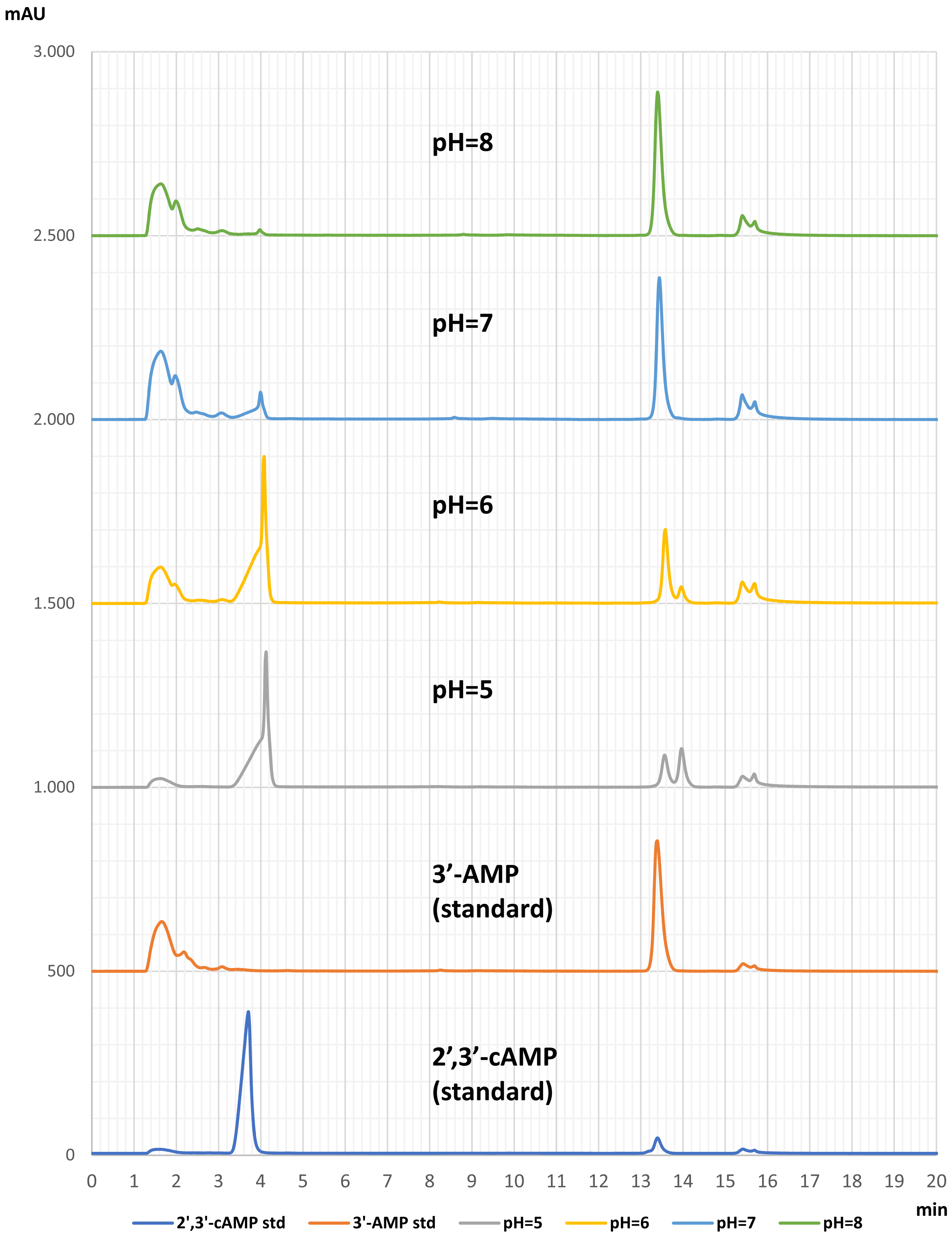

Figure S58. Ion exchange chromatograms of the effect of the pH of a reaction mixture of 3’-AMP (1 µmol, 1eq), DAP (5eq), $MgCl_2$ (5eq) and **2-methylimidazole** (5eq) in 1mL of water at -20°C (*week 4*).

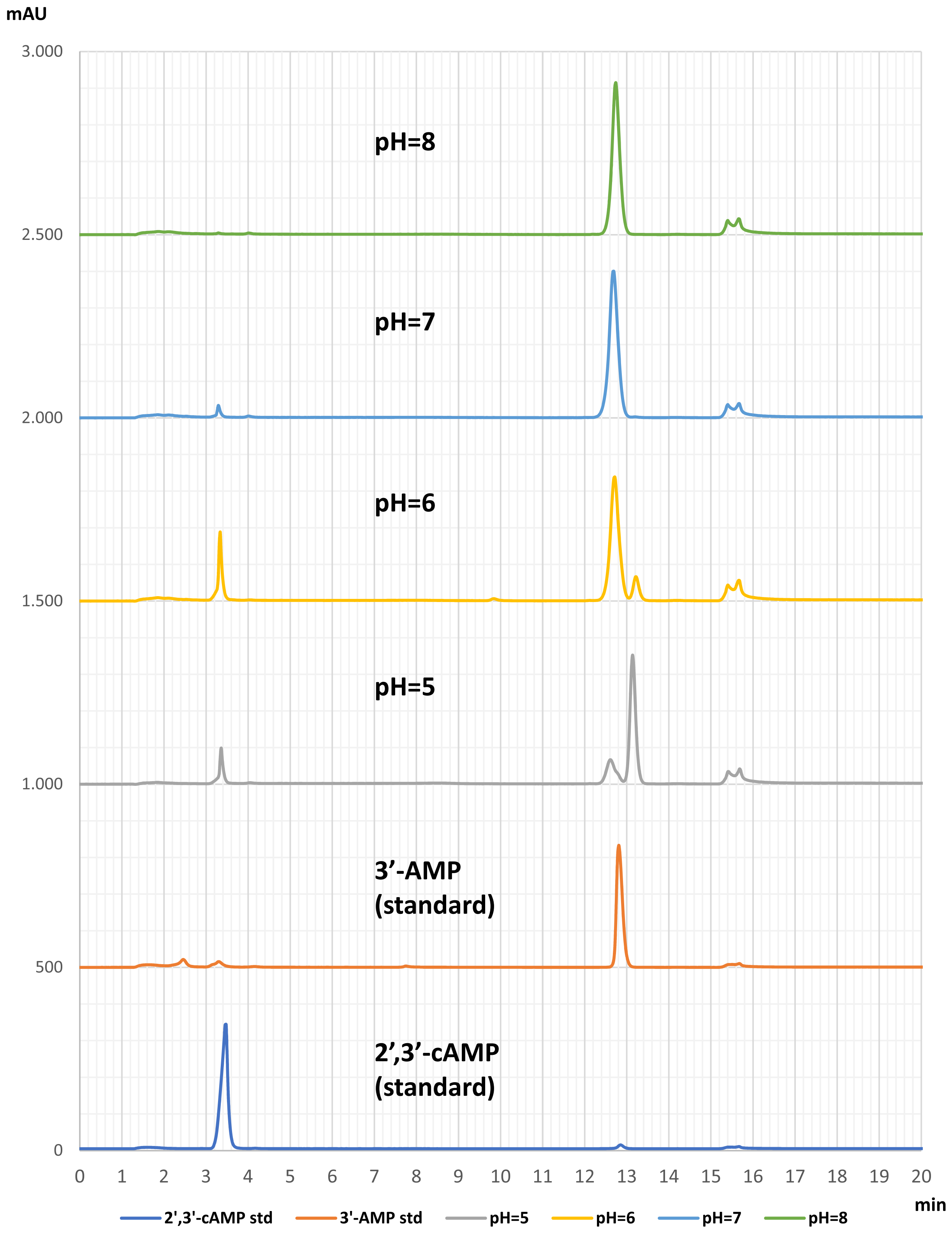

Figure S59. Ion exchange chromatograms of the effect of the pH of a reaction mixture of 3’-AMP (1 µmol, 1eq), DAP (5eq), $MgCl_2$ (5eq) and **2-aminoimidazole** (5eq) in 1mL of water at -20°C (*week 1*).

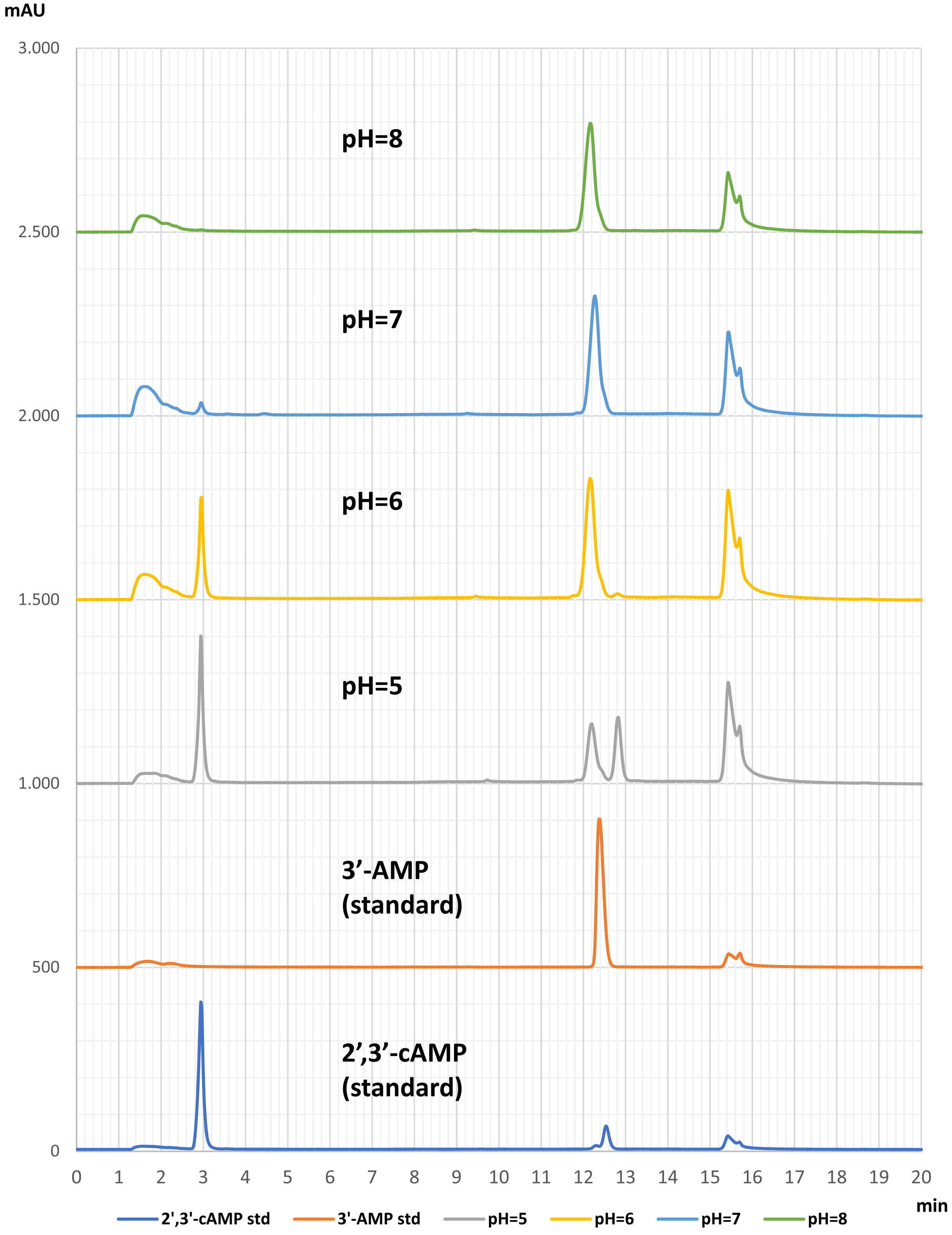

Figure S60. Ion exchange chromatograms of the effect of the pH of a reaction mixture of 3’-AMP (1 µmol, 1eq), DAP (5eq), $MgCl_2$ (5eq) and **2-aminoimidazole** (5eq) in 1mL of water at -20°C (*week 2*).

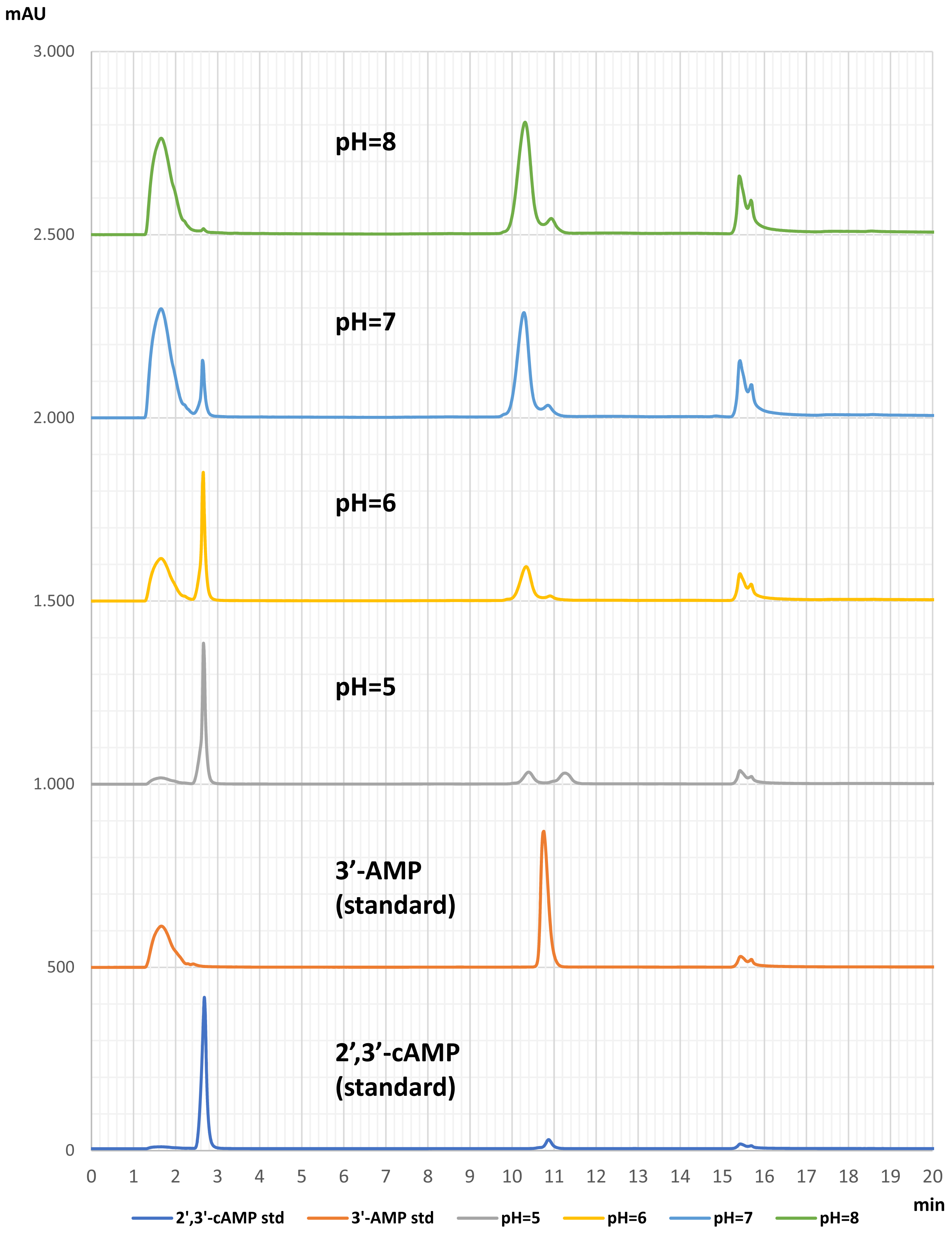

Figure S61. Ion exchange chromatograms of the effect of the pH of a reaction mixture of 3’-AMP (1 µmol, 1eq), DAP (5eq), $MgCl_2$ (5eq) and **2-aminoimidazole** (5eq) in 1mL of water at -20°C (*week 3*).

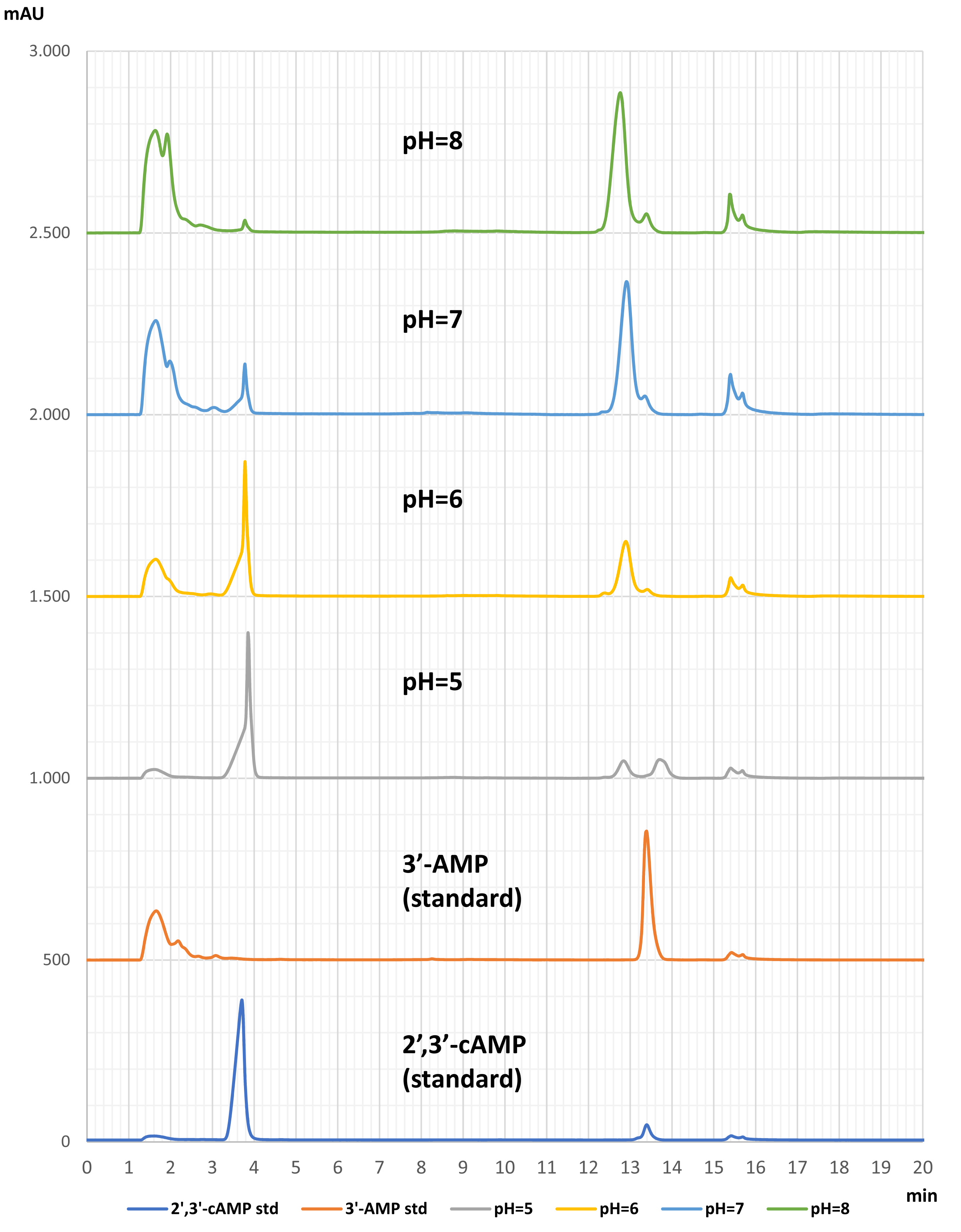

Figure S62. Ion exchange chromatograms of the effect of the pH of a reaction mixture of 3'-AMP (1 µmol, 1eq), DAP (5eq), $MgCl_2$ (5eq) and **2-aminoimidazole** (5eq) in 1mL of water at -20°C (*week 4*).

7.4 Chromatograms 3’-GMP → 2’,3’-cGMP with different activators (weeks 1 to 4)

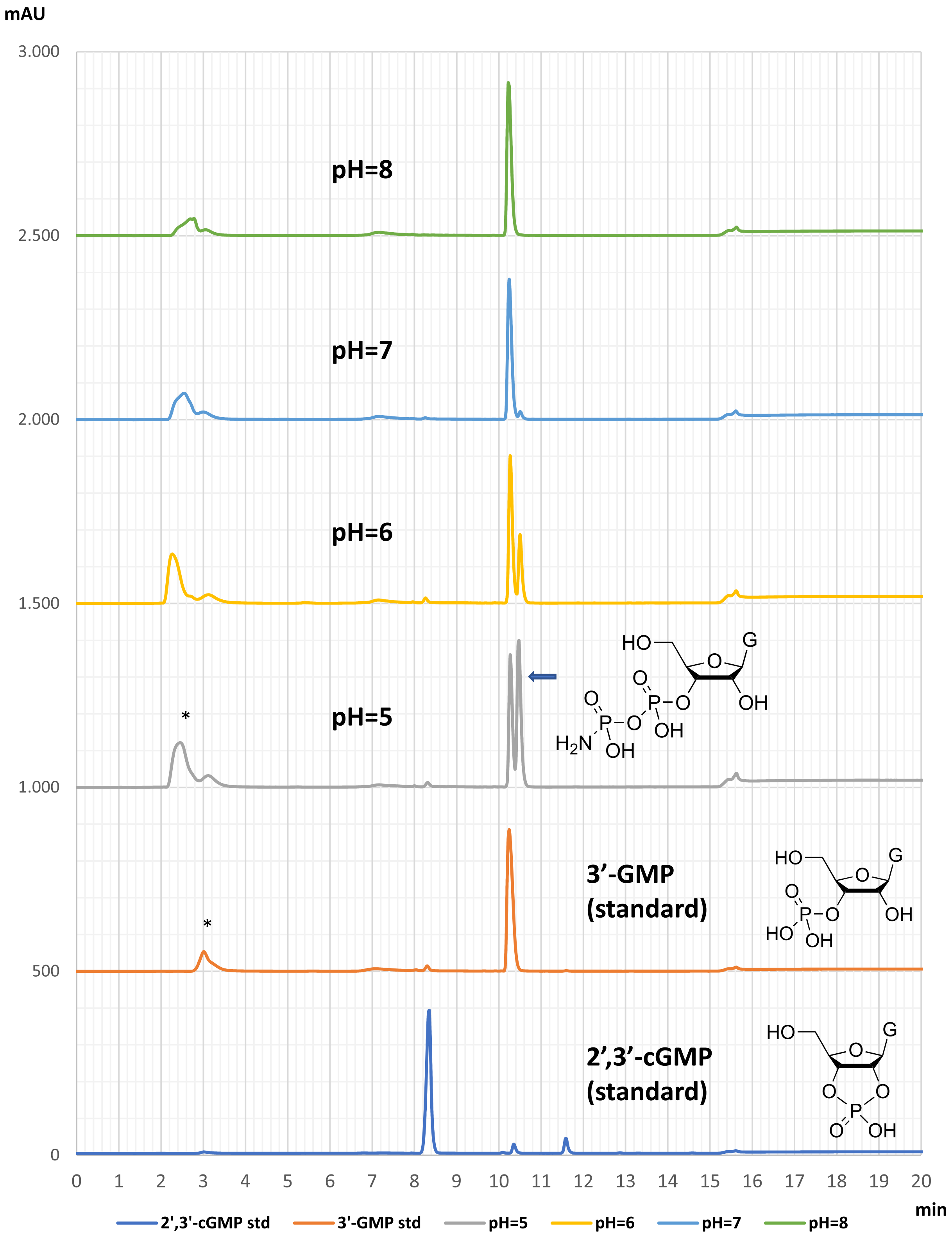

Figure S63. Ion exchange chromatograms of the effect of the pH of a reaction mixture of 3’-GMP (1 µmol, 1eq), DAP (5eq), $MgCl_2$ (5eq) and **imidazole** (5eq) in 1mL of water at -20°C (*week 1*).
* unknown peaks – could be the parent nucleoside.

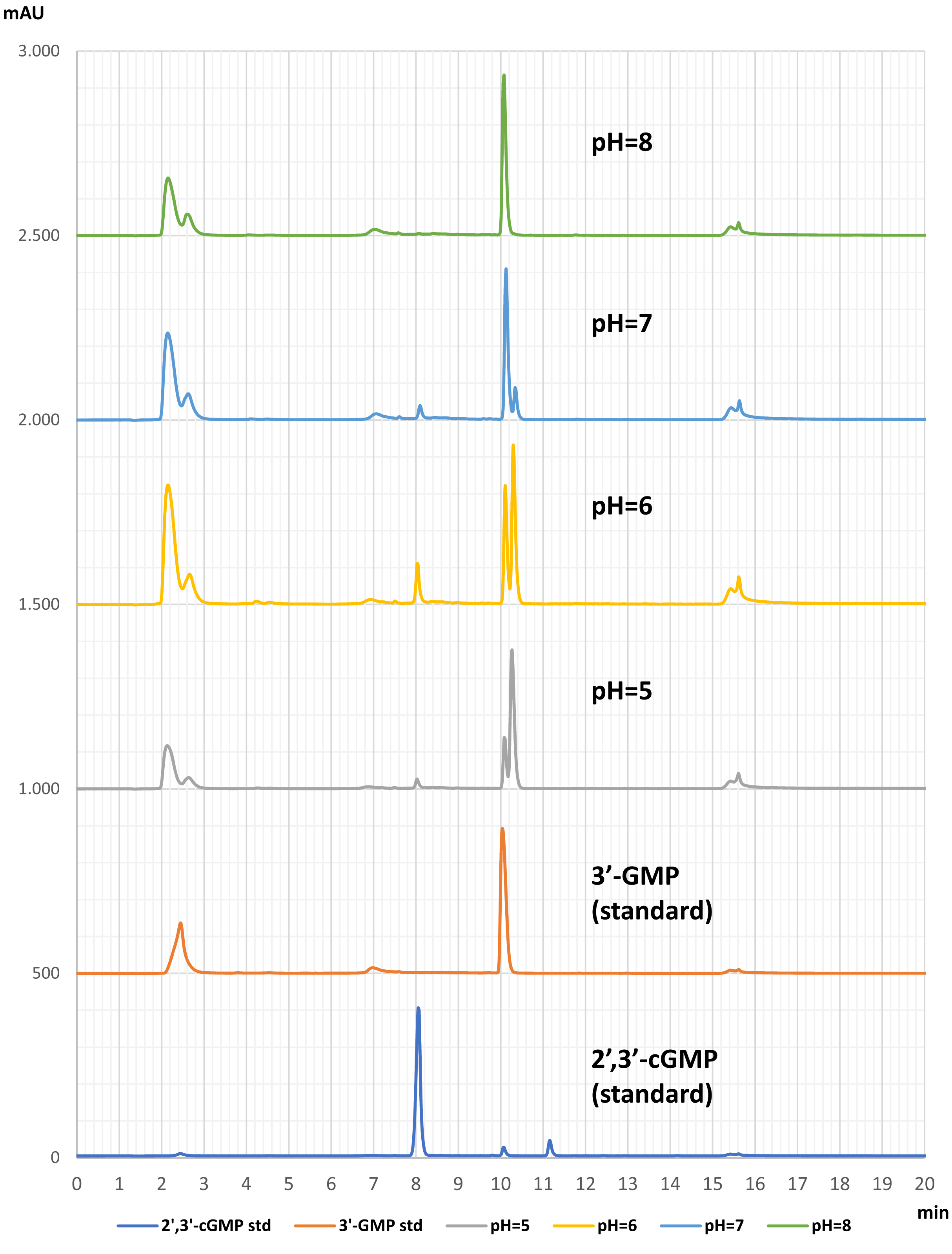

Figure S64. on exchange chromatograms of the effect of the pH of a reaction mixture of 3’-GMP (1 µmol, 1eq), DAP (5eq), $MgCl_2$ (5eq) and **imidazole** (5eq) in 1mL of water at -20°C (*week 2*).

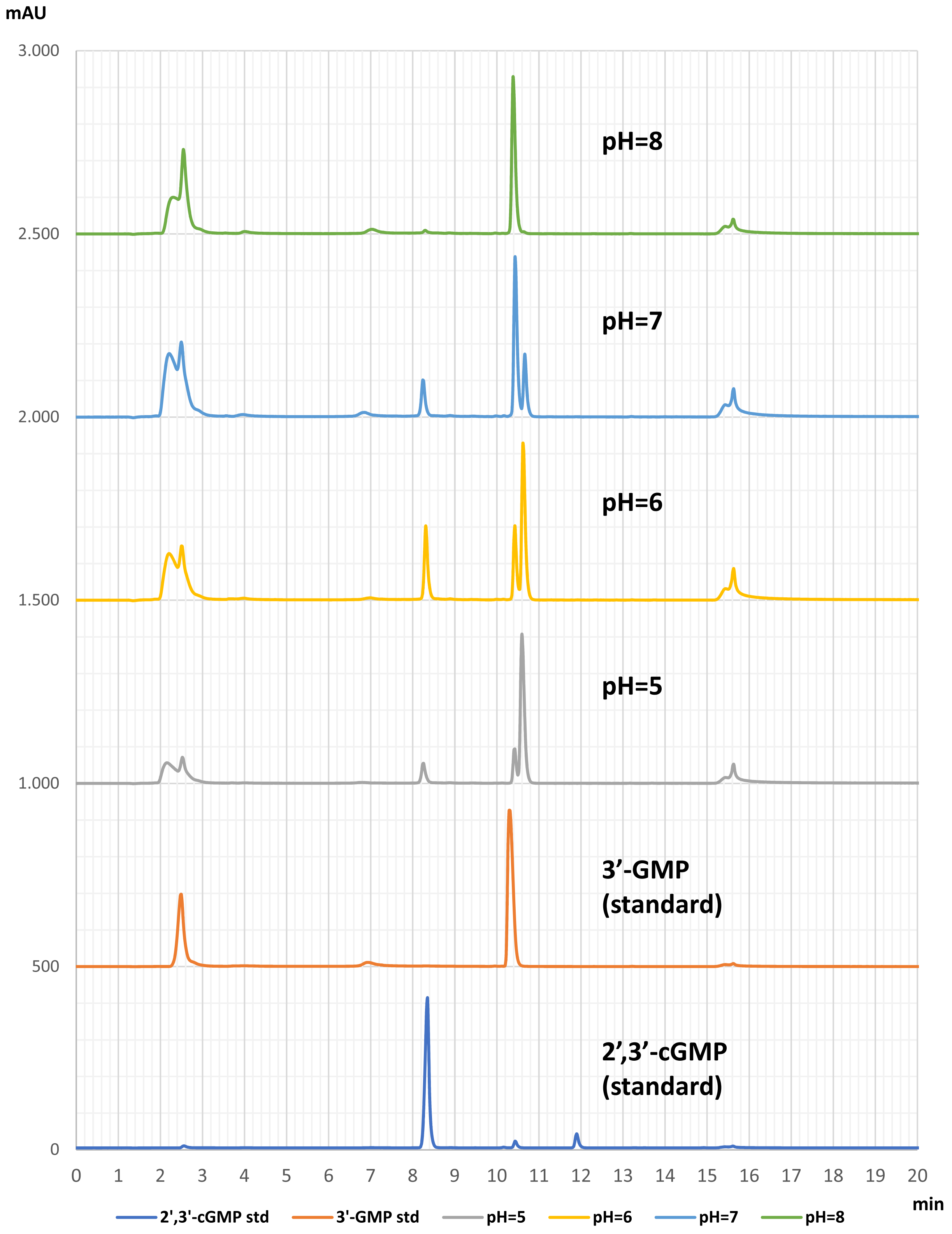

Figure S65. Ion exchange chromatograms of the effect of the pH of a reaction mixture of 3’-GMP (1 µmol, 1eq), DAP (5eq), $MgCl_2$ (5eq) and **imidazole** (5eq) in 1mL of water at -20°C (*week 3*).

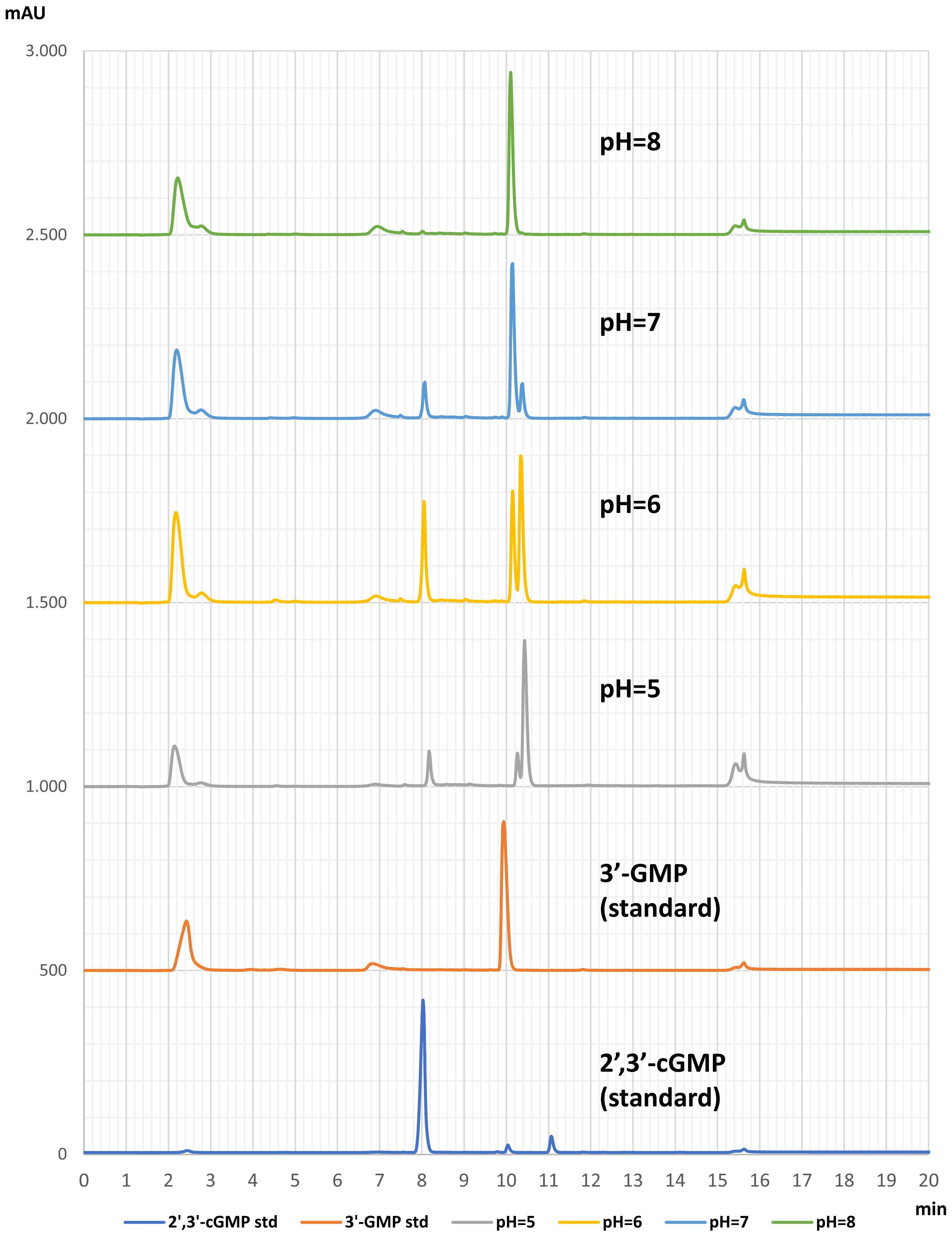

Figure S66. Ion exchange chromatograms of the effect of the pH of a reaction mixture of 3’-GMP (1 µmol, 1eq), DAP (5eq), $MgCl_2$ (5eq) and **imidazole** (5eq) in 1mL of water at -20°C (*week 4*).

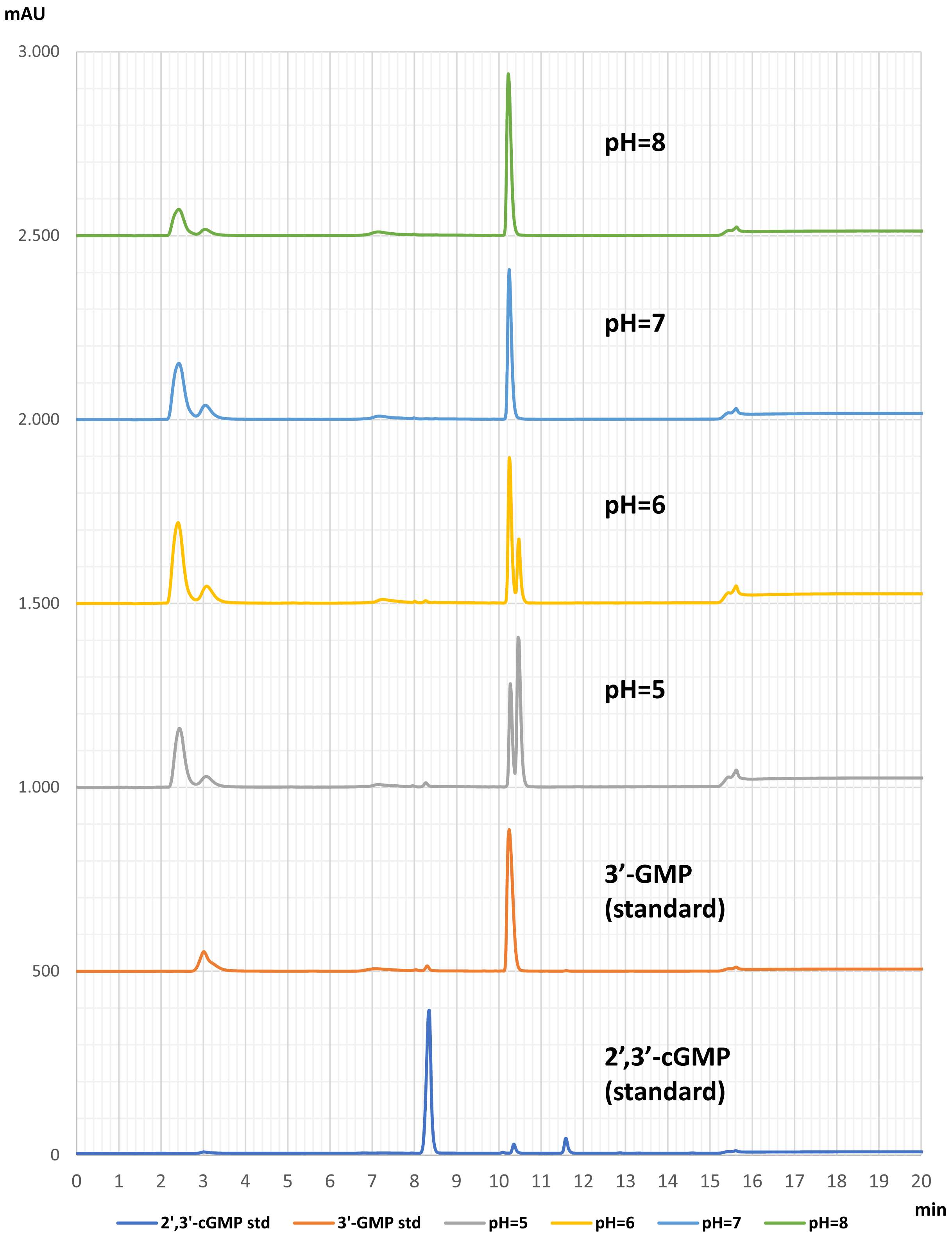

Figure S67. Ion exchange chromatograms of the effect of the pH of a reaction mixture of 3’-GMP (1 µmol, 1eq), DAP (5eq), $MgCl_2$ (5eq) and **2-methylimidazole** (5eq) in 1mL of water at -20°C (*week 1).*

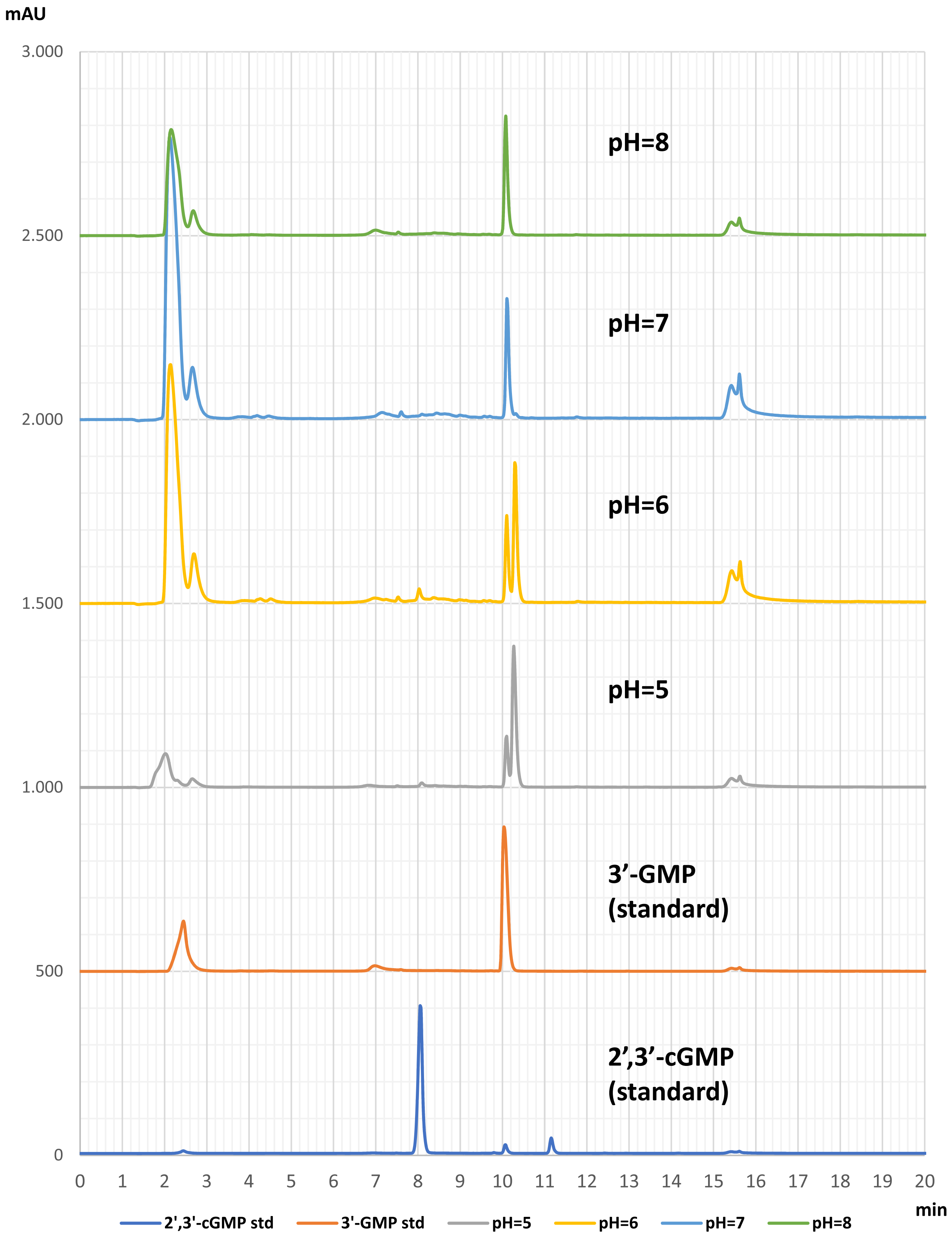

Figure S68. Ion exchange chromatograms of the effect of the pH of a reaction mixture of 3’-GMP (1 µmol, 1eq), DAP (5eq), $MgCl_2$ (5eq) and **2-methylimidazole** (5eq) in 1mL of water at -20°C (*week 2*).

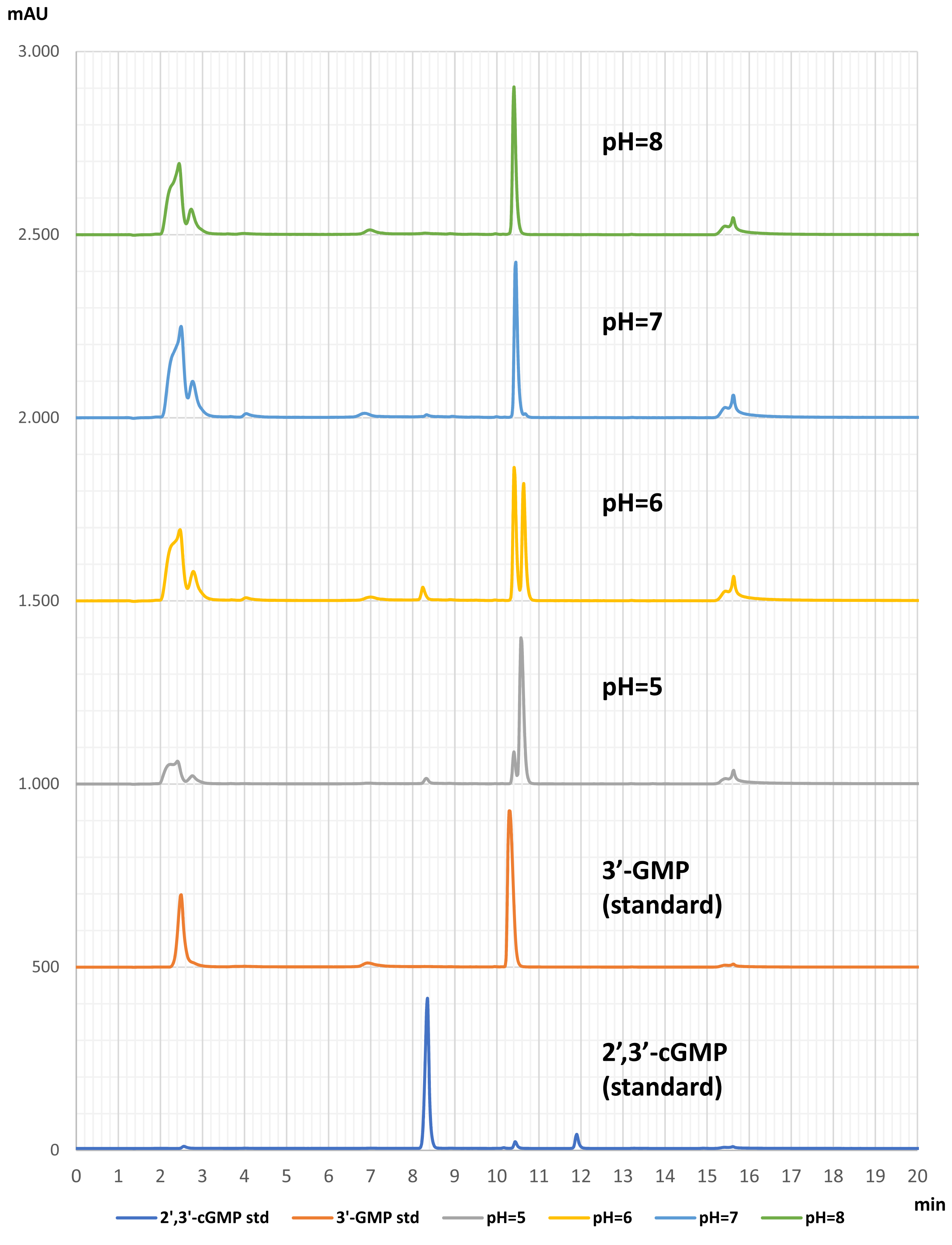

Figure S69. Ion exchange chromatograms of the effect of the pH of a reaction mixture of 3’-GMP (1 µmol, 1eq), DAP (5eq), $MgCl_2$ (5eq) and **2-methylimidazole** (5eq) in 1mL of water at -20°C (*week 3*).

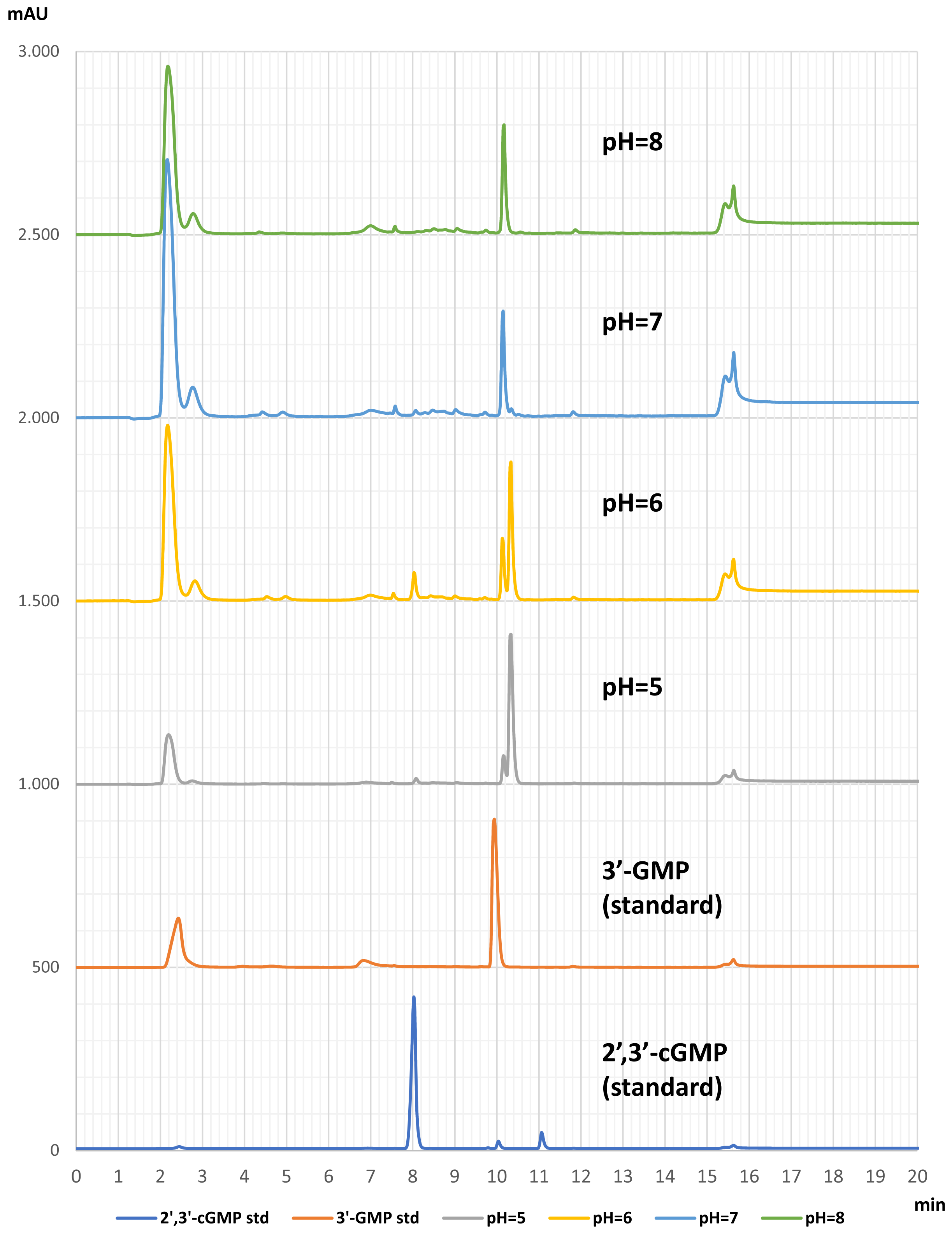

Figure S70. Ion exchange chromatograms of the effect of the pH of a reaction mixture of 3’-GMP (1 µmol, 1eq), DAP (5eq), $MgCl_2$ (5eq) and **2-methylimidazole** (5eq) in 1mL of water at -20°C (*week 4*).

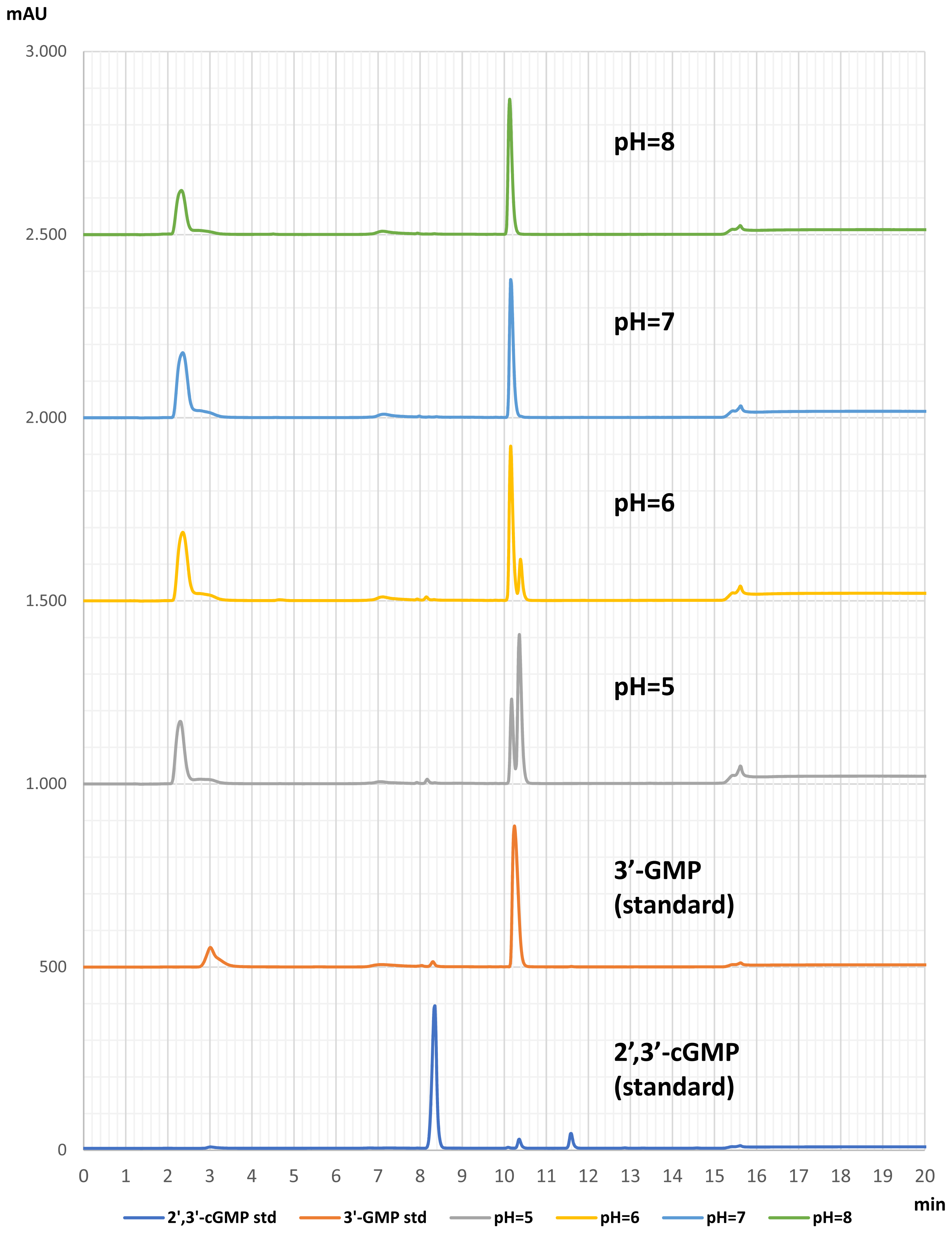

Figure S71. Ion exchange chromatograms of the effect of the pH of a reaction mixture of 3’-GMP (1 µmol, 1eq), DAP (5eq), $MgCl_2$ (5eq) and **2-aminoimidazole** (5eq) in 1mL of water at -20°C (*week 1*).

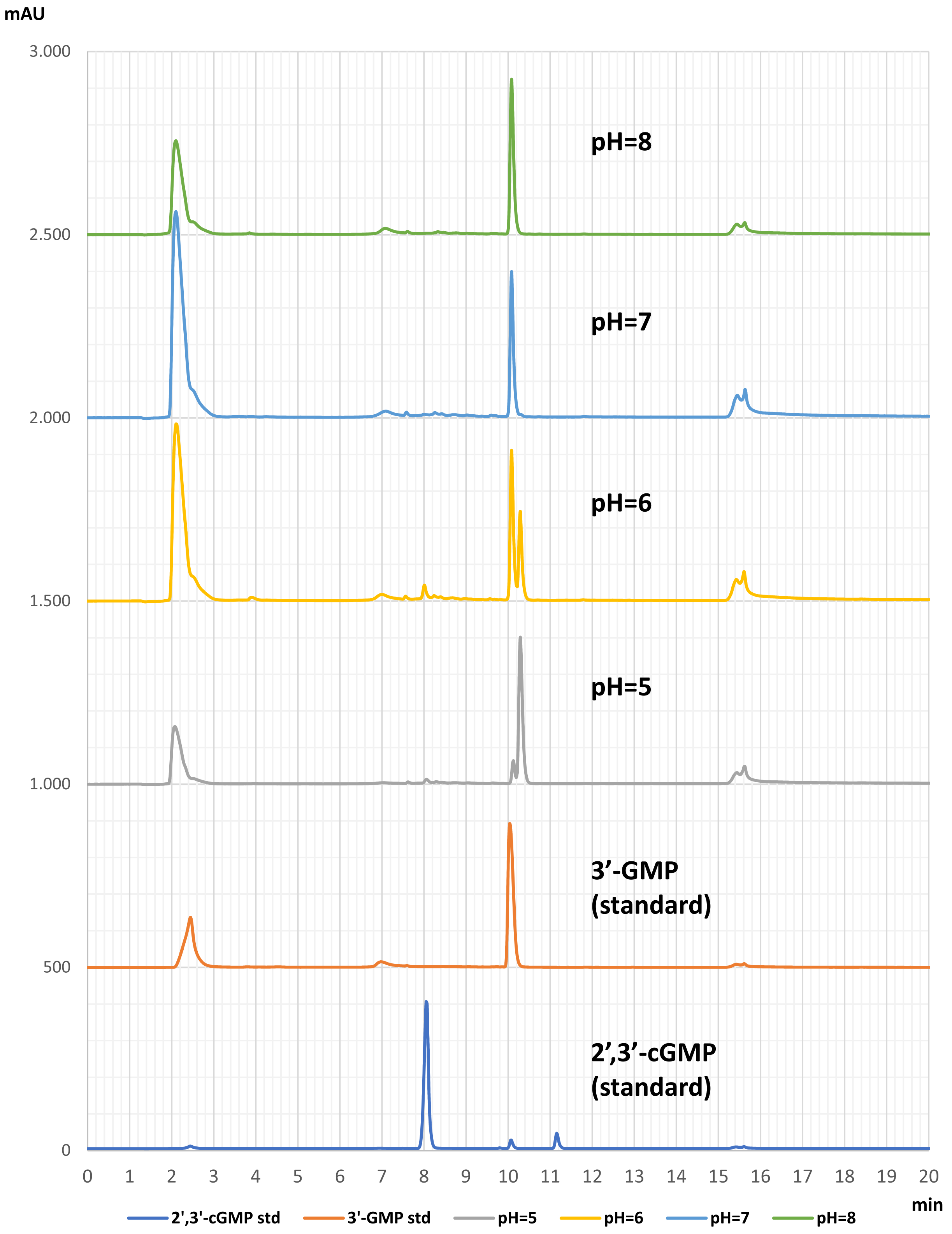

Figure S72. Ion exchange chromatograms of the effect of the pH of a reaction mixture of 3’-GMP (1 µmol, 1eq), DAP (5eq), $MgCl_2$ (5eq) and **2-aminoimidazole** (5eq) in 1mL of water at -20°C (*week 2*).

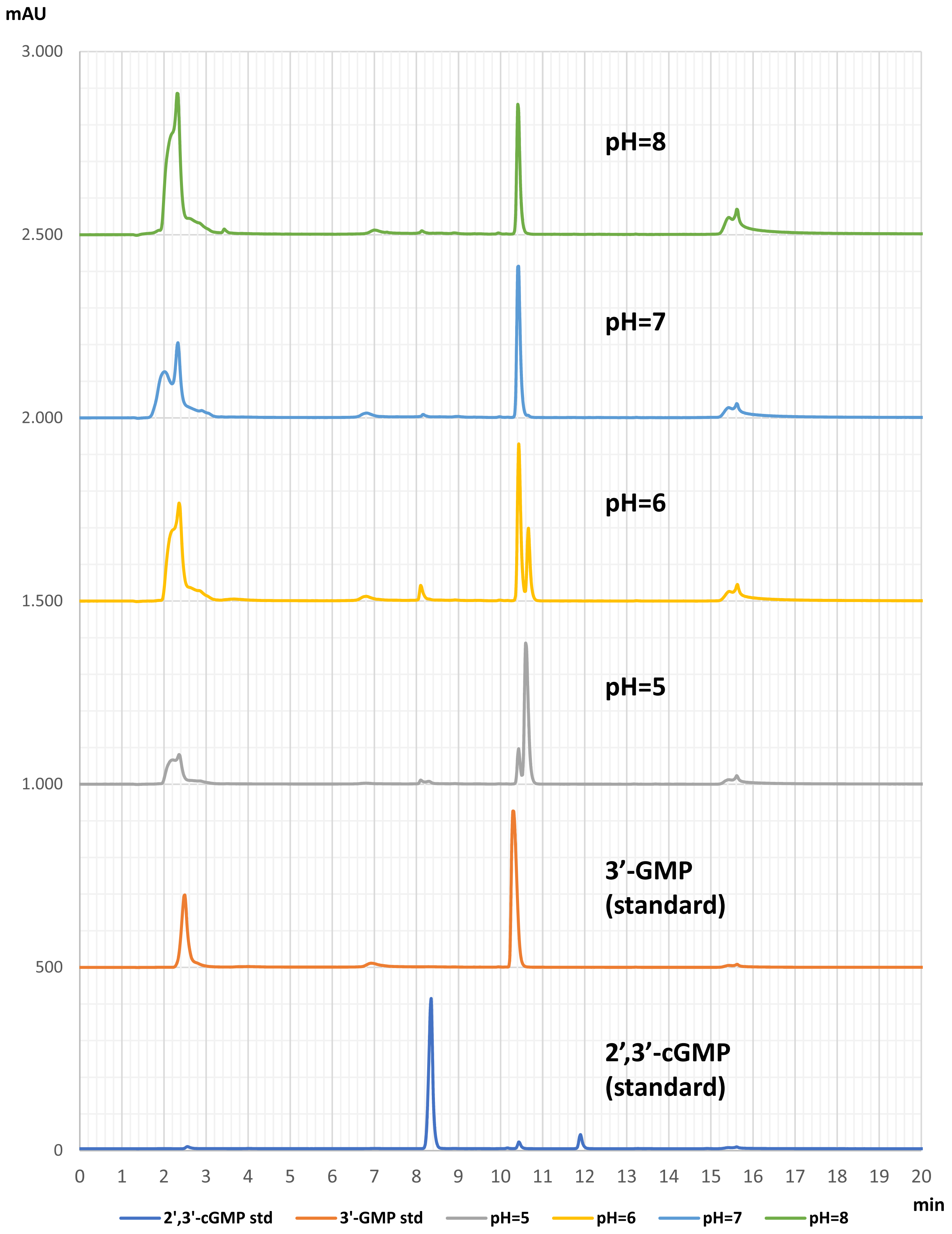

Figure S73. Ion exchange chromatograms of the effect of the pH of a reaction mixture of 3’-GMP (1 µmol, 1eq), DAP (5eq), $MgCl_2$ (5eq) and **2-aminoimidazole** (5eq) in 1mL of water at -20°C (*week 3*).

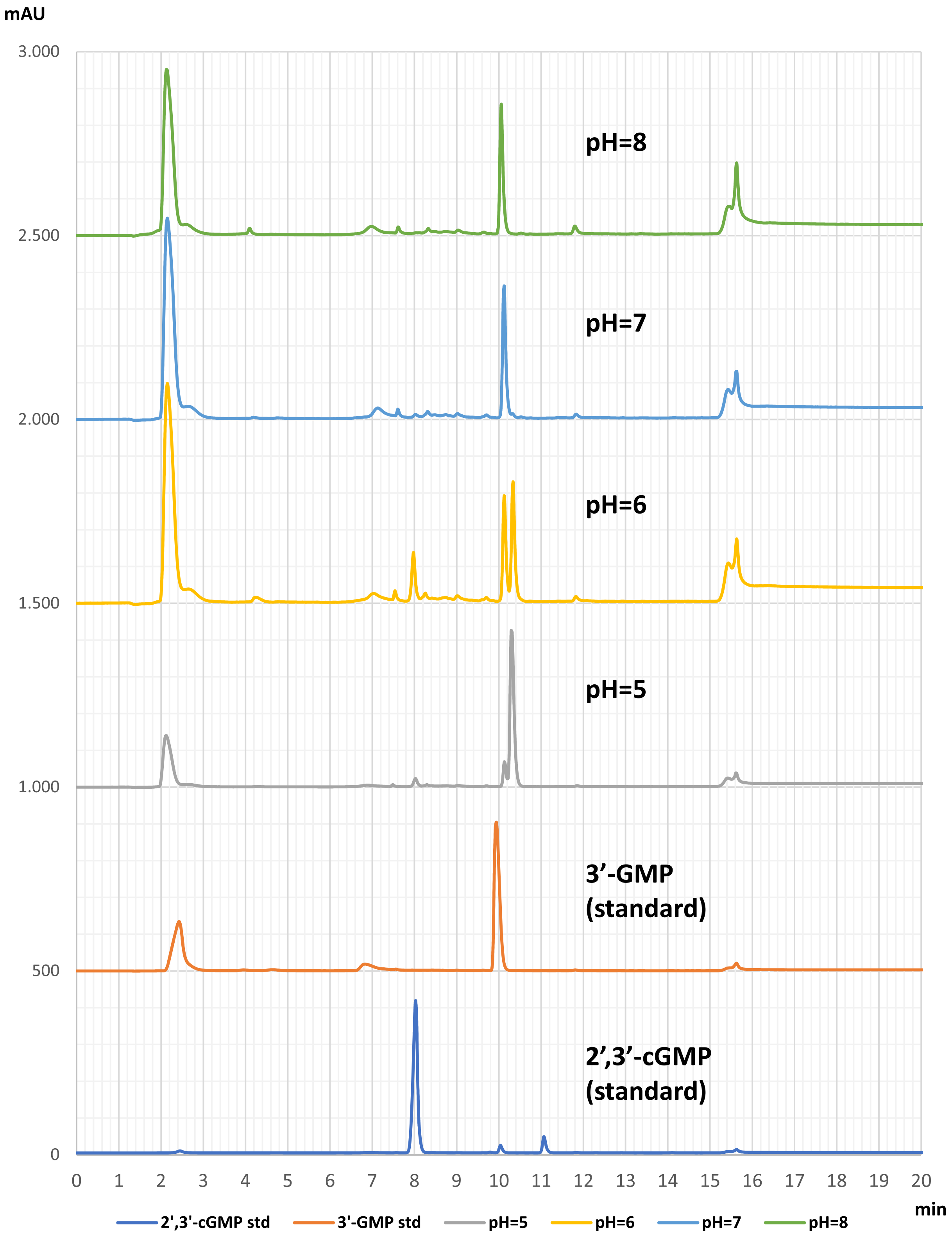

Figure S74. Ion exchange chromatograms of the effect of the pH of a reaction mixture of 3’-GMP (1 µmol, 1eq), DAP (5eq), $MgCl_2$ (5eq) and **2-aminoimidazole** (5eq) in 1mL of water at -20°C (*week 4*).

8. ^{31}P NMR

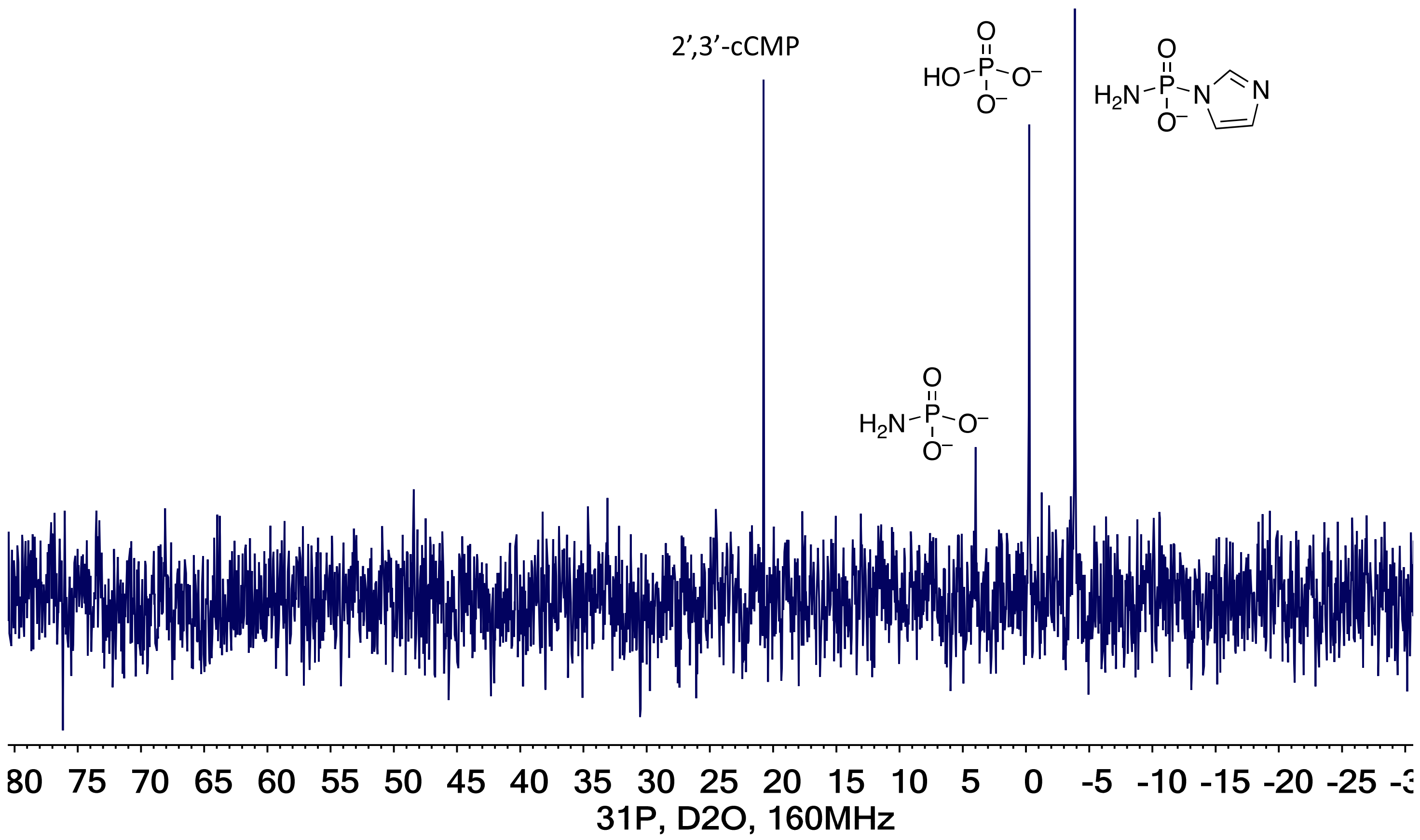

Figure S75. {H-decoupled} ^{31}P NMR spectrum of the reaction mixture of 3’-CMP (1 µmol, 1eq), DAP (5eq), $MgCl_2$ (5eq) and imidazole (5eq) in 1mL of water at -20°C (week 4). The signal at 21 ppm is consistent with the formation of 2’,3’-cCMP. Assignments are based on comparison with Gibard et al (2018).[3]

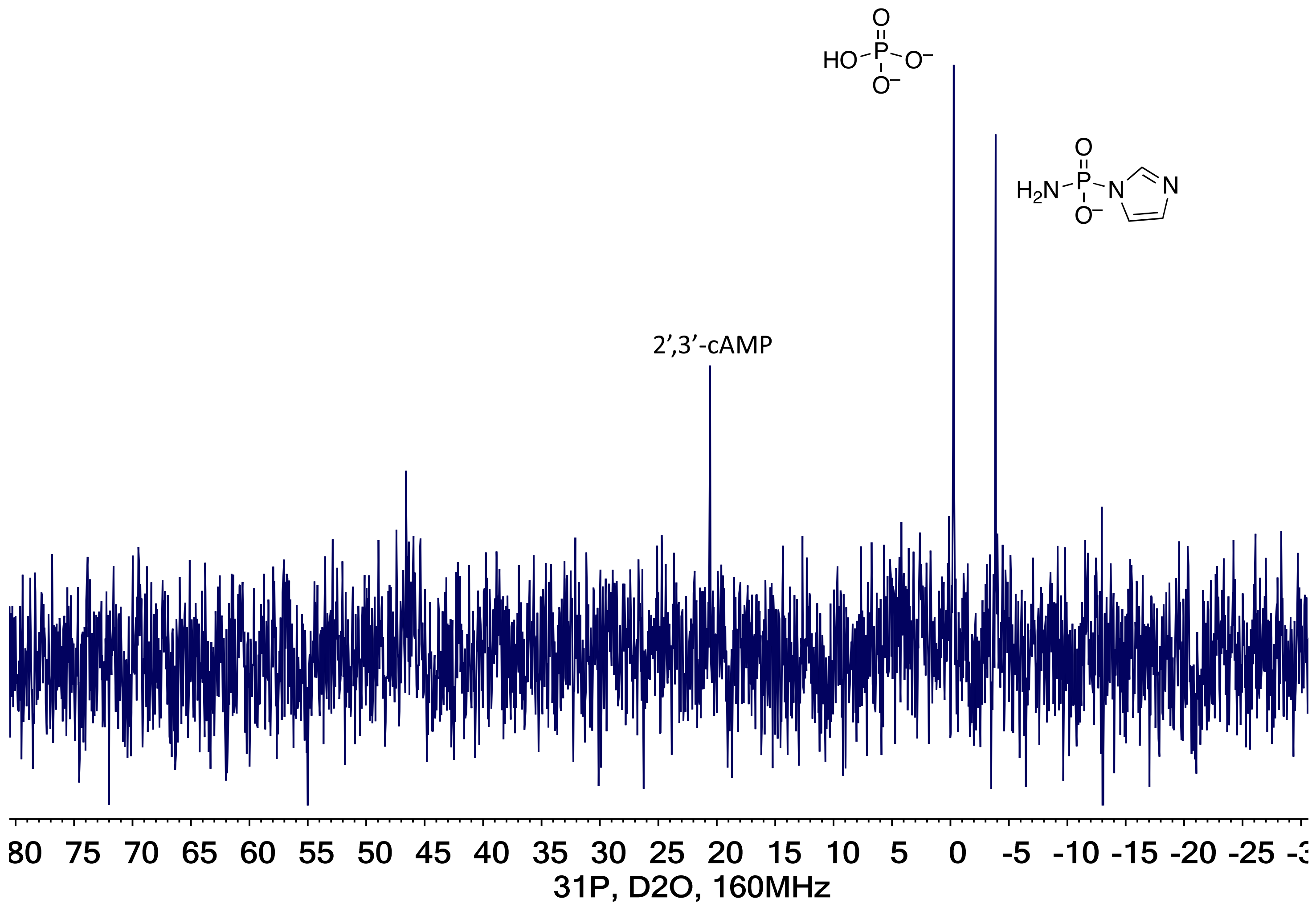

Figure S76. {H-decoupled} ^{31}P NMR spectrum of the reaction mixture of 3’-AMP (1 µmol, 1eq), DAP (5eq), $MgCl_2$ (5eq) and imidazole (5eq) in 1mL of water at -20°C (week 4). The signal at 21 ppm is consistent the formation of 2’,3’-cAMP. Assignments are based on comparison with Gibard et al (2018).[3]

9. Supplementary references

[1] H. Mutschler, A. Wochner, P. Holliger, *Nat. Chem.* **2015**, *7*, 502–508.
[2] J. Attwater, A. Wochner, V. B. Pinheiro, A. Coulson, P. Holliger, *Nat. Commun.* **2010**, *1*, 1–9.
[3] C. Gibard, S. Bhowmik, M. Karki, E. K. Kim, R. Krishnamurthy, *Nat. Chem.* **2018**, *10*, 212–217.

10. Author Contributions

RK and HM conceived the project. All authors designed the experiments. EYS performed and analyzed all experiments involving oligoribonucleotides, with the exception of concatenation and RPR7 assembly experiments which were performed by KLV and analyzed by EYS. EIJ and HL performed and analyzed all monoribonucleotide-involving experiments. EYS, RK, and HM wrote the manuscript with input from EIJ and HL. EYS, EIJ, HL, KLV, RK, and HM discussed the results and commented on the manuscript.
